
JOURNAL INFORMATION
==============================
NLM Title Abbreviation: J Orthop Traumatol
Journal ID: J Orthop Traumatol
NLM Journal ID: 101090931

Journal of Orthopaedics and Traumatology : Official Journal of the Italian Society of Orthopaedics and Traumatology

ISSN: 1590-9921
EISSN: 1590-9999
Publisher: Springer

ARTICLE INFORMATION
==============================
PMCID: PMC4417794
DOI: 10.1007/s10195-013-0258-7
Article ID: 258
Article version: 1
Subjects: Events

In-Depth Oral Presentations and Oral Communications

Electronic publication date: 2013 Sep 18
Print publication date: 2013 Oct
Volume: 14
Issue: Suppl 1
First page: 13
Last page: 46
Copyright: © The Author(s) 2013
License: Open AccessThis article is distributed under the terms of the Creative Commons Attribution 2.0 International License (https://creativecommons.org/licenses/by/2.0), which permits unrestricted use, distribution, and reproduction in any medium, provided the original work is properly cited.
License URL: https://creativecommons.org/licenses/by/2.0/

Keywords: Rotator Cuff, Mesenchymal Stromal Cell, Radial Head, Direct Anterior Approach, Pelvic Ring Injury
issue-copyright-statement: © The Author(s) 2013

==============================
J Orthopaed Traumatol (2013) 14 (Suppl 1):S13–S46
DOI 10.1007/s10195-013-0258-7

27 OCTOBER 2013

### In-Depth Oral Presentations and Oral Communications

### IN-DEPTH ORAL PRESENTATIONS

### *A01–ARTHROSCOPY*

### Comparative analysis between clinical tests and arthroscopic investigation in meniscal tears associated with anterior cruciate ligament injury

A. Speziali*, M. De Vita, F. Manfreda, G. Placella, E. Sebastiani, M.M. Tei, P. Antinolfi, G. Cerulli

Let People Move Research Institute (Perugia-Arezzo, IT)

**Introduction** The combined meniscus and anterior cruciate ligament (ACL) tears are commonly reported sport-contact injuries of the knee. The clinical maneuvers more adopted for identifying the meniscus injuries are McMurray test, Apley test, medial and lateral joint lines tenderness (JLT). The purpose of our study is that to evaluate the correlation between clinical examination and arthroscopic findings in associated acute meniscus and ACL injuries.
**Materials and methods** One hundred and thirty seven patients with a mean age of 28.5 years (from 12 to 55), out of one hundred and seventy initially included, were retrospectively examined for acute ACL and meniscus traumatic injuries (from 6 to 8 weeks by the trauma), between March and November 2012 at our department. Clinical examination included McMurray test, Apley test and medial and lateral JLT. Then, the clinical tests were compared to the arthroscopic findings in order to estimate their sensitivity, specificity, accuracy, and lastly their positive and negative predictive value.
**Results** We had found that the Apley test achieved a sensitivity of 51 and 48 %, a specificity of 84 and 79 % for medial and lateral meniscus tears, respectively. The McMurray test reached a sensitivity of 75 and 68 % while the specificity was 76 and 61 % for medial and lateral meniscus tears, respectively. The medial JLT had a sensitivity of 77 and 58 % and a specificity of 66 and 52 % for medial and lateral meniscus tears, respectively. Lastly, the lateral JLT achieved a sensitivity of 39 and 40 % and a specificity of 84 and 83 % for medial and lateral meniscus tears, respectively. Focusing on the accuracy, for the McMurray test it was 76 and 64 %, while for the Apley test it was 70 and 68 % in medial and lateral meniscus tears, respectively.
**Discussion** We had demonstrated that the accuracy of the clinical examination is reduced in associated meniscus and ACL tears. However, the clinical tests achieved a sensitivity and a specificity as those reported by scientific evidence using MRI.
**Conclusions** The associated meniscus and ACL injuries decrease the accuracy of the clinical tests for identifying the meniscus tears. The clinical tests can provide diagnosis of high suspicious, bypassing second level investigation, such as MRI, which cannot guarantee a better accuracy. Arthroscopic investigation remains the gold standard for diagnosis of the meniscus tears.

### *A02–TRAUMATOLOGY*

### Intramedullary nail *versus* arthroplasty: a prospective study for the treatment of borderline fractures of the upper femur

S. Cerbasi*[1], G. Costa[1], P. Recano[1], E. Aitanti[1], M. Misasi[2], M. Mariconda[1]

[1]Dipartimento Ortopedia, Università Federico II (Naples, IT);
[2]I Divisione Ortopedia, AORN Cardarelli (Naples, IT)

**Introduction** The ideal treatment of borderline fractures of upper femur is matter of scientific debate. This multicenter prospective study was carried out to evaluate the outcome of intramedullary (IM) nail *versus* arthroplasty in patients with borderline fractures of the proximal femur.
**Materials and methods** We assessed 4- and 12-month mortality, walking ability, and activities of daily living (ADL scale) in 166 patients (42 males and 124 females; mean age 78.8 ± 10 years) who underwent IM nailing (100 cases) or arthroplasty (66 cases) for AO-ASIF 31B2.1 hip (52 cases) or AO-ASIF 31-A1.1 and 31-A1.2 trochanteric fracture (114 cases) between January 2010 and January 2012 at our departments. Models of linear and logistic multivariate analysis were constructed to evaluate the effect of the surgical treatment (IM nail or arthroplasty) on functional outcomes and mortality, adjusting the analysis for age, gender, type of fracture, co-morbidity (CIRS scale), cognitive functioning (MMSE), and pre-fracture functional level.
**Results** The overall mortality at 1 month, 4 months, 12 months was 2.4, 10.8 and 15.7 %, respectively. Fifty-three percent of patients treated with IM nail and 36 % of those treated with prosthesis walked independently without aids 12 months after the fracture ($p = 0.007$). Forty-five percent of patients in both therapeutic groups obtained the maximum score on the ADL scale12 months after the fracture. At the multivariate analysis, arthroplasty was associated with higher 4-month (O.R. = 11.9; 95 % C.I. = 2.7–52.0) and 12-month (O.R. = 5.3; 95 % C.I. = 1.7–16.5) mortality and with poorer 12-month walking ability ($c = -0.5; p = 0.006$) when compared to IM nail. Negative effect of the age and of a poor pre-fracture cognitive and functional status on mortality and functional outcomes was also detected. The incidence of local complications and revisions was slightly higher in the IM nail compared to the arthroplasty group.
**Discussion** This is the first prospective comparative study evaluating IM nailing versus arthroplasty for the treatment of borderline fractures of the proximal femur with the use of an expanded set of validated explanatory and outcome variables.
**Conclusions** IM nail was superior to arthroplasty on 12-month mortality and walking ability.

### Femoral cortical index: associations with comorbidities and risk of new fragility fractures

M. Feola*, C. Rao, V. Tempesta, M. Celi, E. Gasbarra, U. Tarantino

Università Tor Vergata (Rome, IT)

**Introduction** The femoral cortical index (FCI) assesses bone stock using the ratio between the diameter of the femoral shaft and the thickness of the cortical bone calculated 10 cm distal to the centre of the small trochanter in an AP view X-ray of the femur. Aim of our study is to evaluate a possible association among low values of FCI, risk factors, co-morbidities and serum 25 hydroxy-vitamin D levels and to establish the importance of FCI as a potential predictor of a new fracture.

**Materials and methods** We conducted a retrospective study on 160 consecutive patients (44 men and 116 women) (range 60 to 103 years) surgically treated for hip fractures in 2012. FCI has been calculated by routine clinical radiographs of the pelvis both on fractured femur and on the opposite side. For each patient, we analyzed the presence of co-morbidities, risk factors and blood levels of vitamin D.

**Results** Average values of FCI were 0.42 (range 0.18–0.58) at the fractured femur and 0.48 at the opposite side (range 0.25–0.66, $p = 0.002$). At the fractured side an average value of 0.45 was found in men, and of 0.40 in women. Patients with severe hypovitaminosis D (serum concentration <12 ng/ml) had a minor FCI compared to those with a moderate deficiency (0.41 vs 0.46, $p < 0.01$). The presence of co-morbidities or osteoporosis risk factors had a different influence on the values of FCI. Lower values of CIF were found on the first fractured femur in patients with bilateral femur fracture.

**Discussion** We found a correlation among low values of FCI, clinical factors related to bone fragility and severe hypovitaminosis D in elderly patients with hip fractures. The lowest levels of CIF found in patients with bilateral fracture of the femur may suggest that this parameter can be considered as a predictor of a new fracture. It is still discussed the association with the values provided by the DXA, because of the low specificity of densitometry.

**Conclusions** FCI could be useful to evaluate bone fragility and predict fracture risk. As osteoporosis causes a cortical bone trabecularization that leads to fracture, this index can therefore give a measure of specific cortical bone at low cost using a X-ray standard examination.

### Medial tibial plateau fractures: treatment, functional assessment and morphological study of the medial fragment

S. Bianco*, F. Tonelli, R. Buzzi

AOUC (Florence, IT)

**Introduction** Medial tibial plateau fractures are complex lesions due to various mechanisms of trauma. These fractures are characterized by different morphologies of the medial fragment. Associated lesions include depression of the centre-lateral part of the plateau, knee subluxation, lesions of lateral ligaments and neuro-vascular structures. These kind of fracture require open reduction, stable fixation and other associated surgery procedures. The aim of this study is to evaluate the morphological type of the medial fragment and the clinical and functional results of operative treatment of medial tibial plateau fractures.

**Materials and methods** Thirty-two patients with medial tibial plateau fracture were included in the present study. All the patients were treated with open reduction and stable fixation from 2001 to 2011. The extent of the medial plateau involvement, the centre-lateral comminution, the height of the medial fragment, the distal dislocation of the tibial articular surface, the extent of subluxation, the widening of tibial condyles and lateral ligamentous lesions were evaluated with X-rays and CT scan. Twenty patients were clinically and functionally evaluated at a minimum follow-up of 13 months (13–132) with Knee Score System and Tegner Activity Score. Signs of post-traumatic arthritis were evaluated analyzing knee weight-bearing X-rays.

**Results** The average articular surface of the medial fragment involved was 35 % on average (range 21–55 %), the centre-lateral comminution was 15 % on average (range 3–25 %), the height of the fragment was 59 mm on average (range 43.4–86.9 mm), the distal dislocation of tibial articular surface was 3.5° on average (range 0°–10°), the widening of tibial condyles was 7 % on average (range 0–17 %), subluxation was 8 % on average (range 0–35 %). Clinical results were excellent in 84 %, good in 11 %, fairly good in 4 %. Radiographic signs of post-traumatic arthritis were evaluated in 58 % of cases.

**Discussion** Two different traumatic mechanisms are identified in medial tibial plateau fractures. A varus axial-compression trauma determinates distal dislocation of the tibial articular surface, widening of tibial condyles, femoral subluxation and centre-lateral comminution. A varus-iperextension trauma determinates lateral ligamentous lesions with anteromedial comminution of the medial tibial plateau. Longer surgery times in the presence of a centre-lateral comminution are frequent in order to obtain anatomical reduction of the fracture. This leads to worse prognosis.

**Conclusions** Morphological study of the medial fragment is essential to obtain anatomic reduction intraoperatively. Despite satisfactory functional results in most cases we have to be aware of developing of post-traumatic arthritis with persistent pain and functional limitation.

### Treatment of long bone defects with the induced membrane technique

S. Gudipati*, P. Harwood, V. Ciriello, N. Kanakaris, P. Giannoudis

Academic Department of Trauma & Orthopaedic Surgery, University of Leeds, Teaching Hospitals NHS Trust (Leeds, UK)

**Introduction** We present our institutional results in a cohort of patients with bone defects who were treated with the use of the induced membrane technique.

**Materials and methods** This is a prospective study under taken at a regional tertiary referral centre from January 2008 to December 2012. Inclusion criteria: septic non-union, acute fracture with bone loss and chronic osteomyelitis. Pathological fractures with bone loss were excluded. Data collection included demographics, pathology, previous surgical intervention, complications, size of bone defect and time-to-union. The cement spacer was removed after a minimum of 8 weeks in situ and the defect area following appropriate selection of implant stabilisation was grafted as per the principles of the diamond concept. The minimum follow-up was 12 months.

**Results** Fifteen patients (12 males) with bone loss after debridement of a septic non-union or an acute fracture were eligible to participate in the study. The mean age was 50 years of age (range 18–80 years). The mean length of the defect was 5 cm (range 2–12 cm). In 7 patients the above technique was applied following an acute fracture while in the remaining 8 cases it was for septic non-union. Radius was involved in 6 patients (in 4 patients for acute fracture, 1 with septic non-union and 1 with chronic osteomyelitis) followed by femoral involvement in 5 patients (septic non-union in 3 patients, for acute fracture management in 2 patients). Tibia was involved in 3 patients all with septic non-union. In one patient metatarsal was involved with acute bone loss following a compound fracture. All patients except one had previous grade 2 or 3 open fractures. One had a chronic osteomyelitis following fracture fixation 20 years previously. All of them healed clinically and radiologically by 8 months (4–20 months). Functionally most of them were able to perform their daily living activities with upper limb injuries recovering to almost near normal range of motion. Two patients had 1–1.5 cm leg length discrepancy, 1 had damage to superficial radial nerve.

**Discussion** The induced membrane technique appears to be an alternative good option for the treatment of large bone defects secondary to acute bone loss or as a result of chronic infected non-unions as seen in this series of patients.
**Conclusions** The induced membrane technique should be considered in the surgeon's armamentarium as it is effective and is associated with a low incidence of complications.

### Pelvic ring injuries in children: long term results

A. Valassina, V. Ciriello*, G. Mazzitelli

Università Cattolica del Sacro Cuore (Rome, IT)

**Introduction** Pediatric pelvic ring injuries are rare lesions (incidence: 1/100000 people per year). Mortality can reach 25 %, even though bony lesion is rarely the direct cause of death. Due to the rarity of these lesions only few published reports exist in the literature with long term results and there is no general consensus about treatment strategies. Aim of the study is to report the long-term results about the treatment of pelvic ring injuries and to suggest new treatment strategies based on biomechanical-developmental criteria.
**Materials and methods** All the patients with an age from 0 to 14 years and pelvic ring injury were included in the study. Type of fracture (modified Torode and Zieg classification system), associated organ lesions, type of treatment and complications were recorded. The outcome measures adopted were: Majeed score, radiographic evaluation (Keshishyan's method) and the measure of leg length discrepancy.
**Results** Twelve patients were included in the study (median age: 9.9 years, range: 1–14). The types of fracture were: 1 patient with type I, 1 with type II (gunshot injury), 4 with type IIIA (out of which 2 with quadrant lesions), 2 with type IIIB and 4 with type IV. Five (42 %) patients presented hemodynamic instability. Associated injuries were: traumatic brain injury (34 %); thoracic injuries (25 %); abdominal and urogenital lesions (25 %). No arterial embolisation was needed. All type IV fractures and the gunshot injury were treated surgically. The mean follow-up time was 6.8 years (range: 1–9 years). At the last follow-up the pelvic asymmetry improved of 51.4 % and the median Majeed score was 80/80. Only one patient with significant leg length discrepancy (3 cm) showed poor result in terms of pain and function. No other complications were recorded. No patient died.
**Discussion** Pelvic ring injuries in children are rarely associated with life-threatening haemorrhages. The biomechanical characteristics of the paediatric pelvis make the quadrant dislocations and stable ring lesions more frequent until 7–8 years of age. After this age pelvic lesions become even more similar to the adult types. The potential of residual growth and remodelling process allow to compensate even high grade of dislocations. Nevertheless, the characteristics of the growth process don't allow to compensate vertical displacement.
**Conclusions** Unstable pelvic ring injuries have to be managed surgically by ORIF even in the children. Stable lesions and quadrant dislocations may be treated conservatively, even if the fractures with vertical displacement >1 cm have little chance of remodelling and need surgical treatment.

### *A03–HIP*

### Comparison of clinical outcome and patient satisfaction between direct anterior and lateral approach in hip arthroplasty

C. Trevisan*, W. Recalcati, R. Klumpp, V. Nava, D. Riccardi

UOC di Ortopedia e Traumatologia, Ospedale SS. Capitanio e Gerosa, AO Bolognini (Seriate, IT)

**Introduction** The minimally invasive direct anterior approach (DAA) has recently been rediscovered as it allows the implantation of a hip prosthesis through a inter-nerve interval not requiring the section of any muscle. Limited data are available on clinical outcomes at follow-up and on patient satisfaction.
**Materials and methods** A consecutive series of patients with primary osteoarthritis was recruited: 39 were operated with modified Hardinge approach (group 1) and 30 with DAA (group 2). Peri- and post-operative complications, the Harris Hip Score and component placement were evaluated at a mean follow-up of 20 months (6–36). The subjective experience of pain during hospitalization and patient satisfaction with regard to intervention were also assessed with a specific questionnaires.
**Results** There was 4 peri-operative complications: a fracture of the calcar in group 1 and 2, 1 cortical perforation and 1 paresis of NFCL in group 2. None of these complications delayed functional recovery. There was no hematoma, wound dehiscence, infection or dislocation. The Harris Hip Score (HHS) at follow-up was significantly better in group 2 ($92.2 \pm 11.9$ *vs* $97.2 \pm 4.5$ $p = 0.04$, calculated by Student's *t* test). Femoral stem positioning was no significantly different between group 1 and 2 while cup inclination was significantly better in the group 2 ($40.6° \pm 6.6°$ *vs* $44.3° \pm 7.9°$, $p = 0.04$, calculated by Student's *t* test). As regards the subjective recall of the pain experienced, the overall pain remembered by the patient and the pain during movement were significantly lower in group 2 ($2.4 \pm 1.2$ *vs* $1.9 \pm 0.9$ $p = 0.04$ and $2.6 \pm 2.3$ *vs* $1.7 \pm 2$ $p = 0.05$). In the questionnaire on patient satisfaction, the percentage of subjects with the best results was greater in group 2 for all the questions.
**Discussion** The 69 patients in this study are part of a consecutive series of patients operated by the same surgeon who followed the same clinical and rehabilitative protocol. In both groups, peri-operative complications were few and didn't affect the functional recovery. Components positioning proved adequate in both groups. At almost 2 years follow-up, functional outcome expressed by HHS was significantly better in group 2 and the recall of experienced pain and patient satisfaction resulted better in DAA operated patients.
**Conclusions** In this study, DAA has proven reliable and has determined a better functional recovery and greater patient satisfaction.

### Efficacy and tolerability of PR oxycodone-naloxone combination in the management of post-operative pain in patients undergoing hip replacement: a new therapeutic standard?

M. Scardino*, F. Martorell, F. Astore, A. Gurgone, G. Mazziotta, F. Traverso

Humanitas (Milan, IT)

**Introduction** Many patients undergoing hip replacement do not achieve adequate post-operative pain control, leading to suboptimal recovery and mobilization. Oxycodone is effective in controlling post-operative pain, but is associated with adverse events such as post-operative nausea and vomiting (PONV); on the contrary, oral prolonged-release oxycodone-naloxone combination (OXN; Targin, Mundipharma) has been found effective with less side effects.
**Materials and methods** Consecutive patients aged 18–80 years undergoing total hip replacement received peri-operative combined spinal-epidural single-shot anesthesia followed by oral ketoprofen 100 mg and oral OXN 10/5 mg every 12 h for the 4 days after surgery; pain rescue medications included paracetamol 1 g or morphine iv if pain refractory. Clinical assessments were performed at days 1–4 after surgery (T1, T2, T3 and T4). Efficacy endpoints included pain intensity at rest and on movement (NRS score), opioid dose, need of rescue analgesia, and patient satisfaction on a 0–4 scale; side effects and tolerability were also assessed on each post-operative day.
**Results** Pain intensity remained constant at mild severity throughout the observation, both at rest (mean NRS: 1.1, 1.1, 1.2 and 1.2 on T1–T4, respectively; NS) and during movement (2.1, 2.4, 2.1 and 2.0 respectively; NS); no patient reported severe pain. The proportion of patients using rescue analgesia was low (17.0 %, 18.4 %, 12.4 % and 12.1 % respectively, $p < 0.009$); no patient required iv morphine for rescue analgesia. There were 72 % patients "very satisfied" with post-operative pain therapy; no patient was "not satisfied at all". Over the 4 days, moderate (requiring iv metoclopramide 10 mg) or severe (despite metoclopramide) PONV was reported by 0.4/9.9 %, 0.4/4.4 %,0.4/1.1 % and 0/1.1 % of patients, respectively; few other AE were recorded, none of whom severe. Spontaneous bowel movement were recorded in 36.2 %, 38.7 %, 61.7 % and 68.1 % of patients during the hospital stay.
**Discussion** The proportion of patients using rescue analgesia was low (17.0 %, 18.4 %, 12.4 % and 12.1 % respectively, $p < 0.009$); no patient required iv morphine for rescue analgesia. There were 72 % patients "very satisfied" with post-operative pain therapy; no patient was "not satisfied at all". Over the 4 days, moderate (requiring iv metoclopramide 10 mg) or severe (despite metoclopramide) PONV was reported by 0.4/9.9 %, 0.4/4.4 %, 0.4/1.1 % and 0/1.1 % of patients, respectively; few other AE were recorded, none of whom severe. Spontaneous bowel movement were recorded in 36.2 %, 38.7 %, 61.7 % e 68.1 % of patients during the hospital stay.
**Conclusions** A simple OXN-based analgesic protocol has been found effective in patients undergoing total hip replacement and was well tolerated with a small number of mild-to-moderate.

### The use of constrained cups for recurrent hip dislocation. A mid and long-term analysis

D. Deledda[1], A. Bistolfi[2], L. Ravera[1], D. Malino[1], E.C. Cenna[2], M. Crova[2]

[1]Università degli Studi (Turin, IT);
[2]Città della Salute e della Scienza, CTO (Turin, IT)

**Introduction** Instability and prostheses dislocation after total hip replacement represent a common problem for the surgeon; that complication has not a solution yet. Retentive cups decrease the number of hip dislocations and they are a solution to solve it. The loosening of the acetabular component due to biomechanical stresses transferred from the prostheses to the bone surface is the principal complication. The aim of the study is to evaluate mid and long term clinical results of 73 total hip arthroplasty implanted using LEPINE constrained cup model.
**Materials and methods** Between November 1999 and December 2011, 73 total hip replacement using LEPINE cups have been implanted in a consecutive series of 68 patients. Fifty-two patients were women and 16 were men; the mean age was 74 years (range 49–81 years). Clinical evaluation was performed using the Harris Hip Scoring system (HHS), while the radiographic analysis was obtained evaluating the anatomical position, radiolucent lines and the offset of the prostheses. All cases were implanted with cement. The statistical analysis was performed using an evidence level of $p \leq 0.05$.
**Results** The end of the study was set on December 2011. The mean follow-up time is from 3 to 12 years. Sixteen patients (16 hips) were lost at the last clinical evaluation and 6 died without any problem concerning the hip arthroplasty. The mean HSS total score is 86.21 points (SD $\pm$ 6.32) after the surgery at the last clinical check. The radiographic analysis evaluated the mean abduction angle of the cup, the ante version angle, the mean radiographic difference between hip physiological position and the arthroplasty offset (0.8 cm, SD $\pm$ 0.2) and radiolucent lines surrounding the cup. In the group of the 56 prostheses there was a total of 9 revisions. The revisions were performed for deep infection in 5 hips, aseptic loosening of the acetabular component in 3, and periprosthetic fracture in 1.
**Discussion** Retentive cups has demonstrated good clinical results and survivorship after 2 years, but at the time of the long-term evaluation the survivorship is similar to the literature description. The principal limit of the study is the low number of patients, because retentive cup is used only in selected cases.
**Conclusions** Constrained cups represents nowadays the best suitable solution for patients with many episodes of hip arthroplasty dislocations but they might be used in selected cases due to the high percentage of aseptic loosening.

### *A04–MISCELLANEOUS*

### Comparison between magnetic resonance imaging and ultrasound evaluation of healthy joints in young subjects with severe haemophilia

C. Ruosi*[1], D. Rossi[1], S. Liccardo[1], F. Granata[1], A. Barbato[1], A. Coppola[2], M.N.D. Di Minno[2]

[1]Dipartimento di Ortopedia e Traumatologia, Università "Federico II" (Naples, IT);
[2]Centro di Riferimento Regionale per i Disordini della Coagulazione, Dipartimento di Medicina Clinica e Sperimentale, Università "Federico II" (Naples, IT)

**Introduction** In patients with haemophilia A, as a result of recurrent haemarthrosis (knees, elbows, ankles), occur concomitant inflammatory and degenerative processes, leading to a chronic and disabling arthropathy. In order to assess the presence and severity of subclinical arthropathy in patients with severe haemophilia A, we evaluated healthy joints (i.e., clinically asymptomatic, never affected by severe flooding bleeding obvious) by magnetic resonance imaging (MRI) and, at the same time, by ultrasonography. This has been done also with the aim to validate ultrasonography, in combination with the clinical evaluations, as an effective diagnostic method in those cases in which MRI, due to the high costs, the poor accessibility and the need of sedation for children, shows complex use and can't be used for an organized screening.
**Materials and methods** From May 2010 to May 2011, 20 patients of age with haemophilia were included in the study. On the same day were subjected to clinical evaluation, ultrasound and MRI in the same joint. By way of comparison, the same joints were evaluated in non-haemophiliacs subjects enrolled in the same period. 40 joints were evaluated in the 2 groups: 20 knees, 8 elbows, 12 ankles of 20 patient.
**Results** For the statistical analysis were used the Student's *t* test, the Pearson's linear correlation coefficients (r) and the Chi square. Ultrasonography, has been found: effusion in 80 % of haemophiliacs and in 20 % of non-haemophiliacs; synovial hypertrophy in 55 % of haemophiliacs and in no subject of the control group; cartilage erosion, 80 % of haemophiliacs and in 25 % of non-hemophiliacs. On MRI has been found: effusion in 85 % of haemophiliacs and in 35 % of non-haemophiliacs; synovial hypertrophy in 45 % of haemophiliacs and in no subject of the control group; cartilage erosion in 75 % of haemophiliacs and in 25 % of non-haemophiliacs. The results were statistically significantly correlated between ultrasonography and MRI for both the effusion and the synovial hypertrophy and the cartilage erosion.
**Discussion** The study shows that ultrasonography is a valid tool, able to detect early signs of arthropathy in young patients with haemophilia whom joints has never been affected by haemarthrosis. These data support the hypothesis that sub-clinical small bleedings may led to the first changes in the joints in asymptomatic patients.
**Conclusions** Ultrasonography, due to its sensitivity in the smallest joint damage and its wide availability, may be a useful diagnostic method for the long-term follow-up of haemophiliac patients. More detailed studies will be needed to testify the specificity of ultrasound examinations in the evaluation of haemophilic arthropathy.

### Comparison between hyaluronic acid and platelet rich plasma in the treatment of hip and knee osteoarthritis: preliminary results

M. Abate*[1], C. Schiavone[2], P. Di Gregorio[3], A. Pantalone[1], T. Scuccimarra[1], D. Vanni[1], E. Andreoli[1], V. Salini[1]

[1]Clinica Ortopedica e Traumatologica (Chieti, IT);
[2]Dipartimento Ecografia Internistica, Università ''G. d'Annunzio'' (Chieti, IT);
[3]Servizio di Immunoematologia e Medicina Trasfusionale, Policlinico "SS. Annunziata" (Chieti, IT)

**Introduction** Hip and knee osteoarthritis (OA) are very common in the general population. Among the several conservative treatments available, intra-articular injection of hyaluronic acid (HA) (i.e. visco-supplementation) can restore the normal articular homoeostasis, with positive results on pain and function. Recently, technologies for enhancing tissue healing involve the use of activated platelets retained in fibrin matrix (platelet-rich plasma, PRP) as a source of growth factors. Aim of the present study was to compare the use of HA and PRP in the treatment of hip and knee OA.
**Materials and methods** Twenty patients suffering from grade II-III Kellgren and Lawrence hip and knee OA, respectively, were enrolled. After clinical and functional evaluation (VAS, KOOS, Lequesne, WOMAC), the participants were randomly assigned to the treatment with HA (10 hip OA and 10 knee OA) and with PRP (10 hip OA and 10 knee OA). In both treatment groups, three injections of HA or PRP (each injection after a week interval) were performed, with ultrasound guidance used only when injecting hip joint. Clinical and functional follow-up were performed after 2, 3 and 6 months.
**Results** The randomized treatment groups did not differ for age, sex, BMI, and clinical/functional parameters. At 6 months both groups reported significant improvement ($p < 0.001$) in all the outcome measures (VAS, KOOS, Lequesne, WOMAC) in comparison to baseline. In the comparison between groups, in the short-term (2 and 3 months), patients treated with HA showed better functional outcomes (KOOS, WOMAC scale: $p < 0.002$), while after 6 months KOOS and WOMAC scales ($p < 0.003$) were significantly higher in the PRP group respect to HA. No differences in the VAS score were observed among groups at 2, 3 and 6 months follow-up. Mild adverse events (pain, swelling) were observed only in the PRP group.
**Discussion** In the short-term, the clinical efficacy of HA may be ascribed to the mechanical effects of HA itself (visco-supplementation) and to its protective properties on articular cartilage and soft tissue surfaces of joints (bio-supplementation). On the other hand, the presence of high amount of healing molecules in the PRP, which stimulates the healing processes at cartilage level, explains the long lasting effects of this compound.
**Conclusions** Visco-supplementation and PRP therapies are both effective in the treatment of hip and knee OA. However, in both treatments, the mechanical positive action due to the fluid injection into the articular space and the placebo effect must be taken into account when evaluating the final outcome.

### Depression and post-menopausal osteoporosis: potential relationships and problems

C. Rao*, M. Feola, V. Tempesta, S. Nicolai, C. Niolu, A. Siracusano, U. Tarantino

Università Tor Vergata (Rome, IT)

**Introduction** Several studies have shown that individuals with mental disorders are more likely to have co-morbidities than general population. A defined relationship between depression and osteoporosis has not been constantly reported: two recent meta-analyses have demonstrated a clear link between the two disorders and depression has been considered an important risk factor for osteoporosis.
**Materials and methods** A population of 72 women with a mean age of 59 years (range 47–75), average degree of education of 10.5 years, coming from the orthopaedic (N = 48) and from the psychiatric (N = 25) outpatient departments of our hospital. According to values of the Beck Depression Inventory II (BDI), a self-report test to assess the severity of depression in the last week, the patients were divided into two groups, respectively, 28 subjects with significant depressive symptoms in the last week and 44 non-depressed subjects. All patients were evaluated by densitometric DEXA (dual-energy X-ray absorptiometry).
**Results** The Student's $t$ test for independent samples showed significant values of bone mineral density (BMD) at the hip in depressed women compared to control ($p = 0.03$). The Pearson correlation among the scores of the somatic, cognitive, total BDI subscales and femoral BMD ($p = 0.10$, $p = 0.015$, $p = 0.010$) was significant. The correlation among the scores of the somatic, cognitive and total BDI subscales and the density values of the lumbar spine (L1–L4) was not significant.
**Discussion** Our study shows a statistically significant correlation between the presence of depression (BDI $\geq$ 19) and the severity of osteoporosis measured through the femoral BMD. It is evident, therefore, a state of reduced bone densitometry in people with moderate-to-severe depression.
**Conclusions** Further studies of the correlation between major depression and osteoporosis, through new biochemical and instrumental assessments, might suggest the need to assess bone stock in patients with depression prescribing, if necessary, an anti-osteoporotic therapy that takes into account the low compliance to drug treatment of these patients.

### *A05–TUMORS*

### Surgical treatment of pathologic fractures of the proximal femur due to metastases

B. Rossi*[1], A. Piccioli[2], G. Di Giacomo[1], G. Scudieri[1], G. Maccauro[1]

[1]Università Cattolica Sacro Cuore, Policlinico Gemelli (Rome, IT);
[2]Dipartimento Oncologia Ortopedica, Policlinico Umberto I, Università La Sapienza (Rome, IT)

**Introduction** Pathologic fracture of the proximal femur (PFPF) due to metastases decreases the quality of life and survival of cancer patients. Surgical options are intramedullary nailing (IN), open reduction and plate-screws internal fixation (ORIF) or resection and prosthetic reconstruction (RP). Adjuvant treatments are radiotherapy, concomitant debulking and/or local adjuvants (cement). Several studies validate each technique, but it is difficult to compare relative indications and results, because of both the lack of strong levels of evidence and for the poor prognosis of these patients.
**Materials and methods** This retrospective study included 193 patients treated for PFPF from 1993 to 2012. Preoperative staging, femoral imaging, stratification of patients in according to Capanna-Campanacci Classification and performance status and Mirels' score $\geq$8 for impending fractures. Solitary radioresistant metastases from renal clear cell carcinoma and thyroid and fractures at the neck, inter/sub-trochanteric with cortical destruction, in patients with life expectancy >6 months, were treated with RP with modular tumor prosthesis; conventional endoprosthesis was used for fractures of calcar in plurimetastatic patients with life expectancy <6 months. Locked IN was used for inter/subtrochanteric fractures extended to the shaft or with concomitant cervical lesions, for radiosensitive histotypes, multiple metastases, prognosis <6 months. ORIF was adopted for lateral cortical osteolyses. Cement was used for reinforced osteosynthesis, cemented prostheses and acetabuloplasty. Outcomes were: surgical complications, functional recovery, survival rates. The mean follow-up was 24 months (1–48).

**Results** Eighty IN, 103 RP, 10 ORIF for 92 complete and 101 impending fractures were performed. There were no intra-operative complications, peri-operative mortality nor local recurrence which required implant revision or hip disarticulation. Fifteen patients (8.2 %) suffered from non-fatal deep venous thrombosis. Postoperative complications were dislocation in RP (5.8 %) and superficial infection (3.8 %). Overall survival rate was 42 % at 1 year and 23.5 % at 2 years after surgery; only 10 % of patients undergoing IN survived 2 years post-surgery, while 20 patients (19.41 %) lived more than 3 years after RP and breast cancer, myeloma and renal clear cell carcinomas were the most common malignancies in these long-survivors.

**Discussion** The proximal femur is the most common site of long bones metastases. Only 30 % of patients survive more than 1 year; however, implementation of chemo-radiotherapy and advance of screening techniques have led, on the one hand, to the exponential increase of patients with pathologic fractures and, secondly, to the improvements of their prognosis.

**Conclusions** Definition of decision-making approach and evidence-based treatment recommendations for FPFP is a stated research priority of orthopaedic scientific societies.

### Allograft-prosthesis composite reconstruction of the proximal humerus in bone tumor surgery using a reverse shoulder prosthesis. Report of functional outcome and complications

D.A. Campanacci*, G. Beltrami, G. Caff, P. Cuomo, N. Mondanelli, R. Capanna

Ortopedia Oncologica (Florence, IT)

**Introduction** After resection of a tumour in the proximal humerus, there is a trend to select an allograft-prosthesis composite (APC) for reconstruction, particularly when the abductor mechanism are intact. However, when the rotator cuff has to be sacrificed completely, APC with conventional prosthesis may have postoperative instability and does often not achieve the expected active abduction because of insufficient fulcrum. The reverse shoulder prosthesis was developed for rheumatoid patients with a completely destroyed rotator cuff and is recently giving a new option for reconstruction in oncological patients.

**Materials and methods** From June 1996 to January 2011 forty-nine patients have been treated by allograft prosthesis or composite prosthesis in the humerus. Sixteen patients had received an APC using a reverse shoulder prosthesis. Following the resection of proximal (15) or total humerus (1) a reverse shoulder prosthesis APC was implanted. The resection was intra-articular with an average resection length of 130 mm (range 70–200 mm).In two cases the reverse prosthesis was implanted several years after a failed osteo-articular allograft, thus reparation of the rotator cuff was performed with the available rotator cuff of the previous allograft. At an average follow up of 32 months (4–109), functional outcome, fracture rate, glenohumeral stability, union rate and implant survival were evaluated using clinical examination, X-rays and MSTS functional evaluation system.

**Results** The average score of MSTS functional evaluation system was 76 % (46.7–100 %). In one case the greater tuberosity separated due to resorption so the fragment was removed. Two patients had loosening of the stem after 27 and 52 months. All three revised cases had final satisfactory results (MSTS 73 %) after a mean follow up of 58 months. One patient had primary postoperative instability and luxation of the prosthesis due to an absent rotator cuff on the allograft, which led to revision. One young and very active patient had loosening of the glenoid component after 1 year, which required revision with a custom made mega cup. Both patients achieved a satisfactory functional result after revision.

**Discussion** In selected cases with primary bone tumour or metastasis of the proximal humerus an ACP with reverse prosthesis has proven to be a successful procedure, offering good stability with a wide range of motion. Even in cases with complete removal of the abductor apparatus a functional reconstruction can be performed using a latissimus dorsi flap pro-deltoid. In three out of four cases of revision we were able to salvage the implant with satisfactory final result.

**Conclusions** A long time follow up is definitely indicated for confirmation of our experience regarding mechanical and functional results.

### Latissimus dorsi flap in orthopaedic oncology: coverage or functional flap?

M. Boffano*[1], U. Albertini[1], E. Boux[1], S. Marone[1], A. Ferro[1], D. Ciclamini[2], P. Tos[2], R. Piana[1]

[1]S.C. Ortopedia Oncologica e Ricostruttiva, AO Città della Salute e della Scienza (Turin, IT);
[2]S.S.D. Microchirurgia, AO Città della Salute e della Scienza (Turin, IT)

**Introduction** Latissimus dorsi flap (LDF) is a valid surgical option in orthopaedic oncology to fill in big tissue defects after the excision of bone and soft tissue sarcomas in the upper limb. To increase the functional result in the operated limb it is necessary to substitute the function of the resected muscles with a muscular flap. The aim of this study is to evaluate the possible indications for either pedicled LDF or free LDF.

**Materials and methods** From 2006 to 2012, at an Italian reference centre for the treatment of bone and soft tissue sarcomas, 14 LDFs have been performed in 14 different patients (7 males; average age 52, 5 years old, range 17–78 years). The purpose of the flap (either coverage or functional), the characteristics of the flap (pedicled or free), the grafted area (upper or lower limb) have been evaluated. The function was measured and compared to the function of the opposite limb.

**Results** Six coverage LDFs (5 pedicled flap in the upper limb, 1 free flap in the lower limb) and 8 functional LDFs (6 in the upper and 2 in the lower limb) were harvested. Functional LDFs substituted the deltoid muscle in 5 cases, the triceps, the quadriceps, the glutei in 1 case each. The function was good in the upper limb except for the cases of associated bone resection and reconstruction with prostheses; it was fair in the lower limbs (Mankin's classification).

**Discussion** LDFs are characterized by low morbidity in the donor area, large amount of available tissue, easy harvesting with a strong and trustworthy bundle. This study confirms the reliability of LDF as a coverage flap either pedicled or free. Even if good functional results were obtained in the upper limb, the possibility of using free LDFs to substitute lower limb muscles, such as glutei or quadriceps, is still debated.

**Conclusions** LDFs offer a strong and widespread reconstructive solution, if other surgical options are not available. Further studies will clarify the indications for the use of LDFs within reference centres for orthopaedic oncology.

### Uncemented GMRS modular prosthesis in reconstruction of the lower limb after resection for bone tumors: functional results, complications and a comparative statistical analysis

P. Ruggieri[1], E. Pala[1], E. Henderson[1], G. Trovarelli[1], A. Angelini[1], A. Piccioli[2], M. Romantini[1], T. Calabrò[1], S. Giannini[1]

[1]III Department of Orthopaedics, University of Bologna, Istituto Ortopedico Rizzoli (Bologna, IT);
[2]Department of Orthopaedic Oncology, CTO (Rome, IT)

**Introduction** Lower limb is a frequent site for bone tumours. Due to their availability, modularity, uncomplicated usage, immediate fixation, and relatively low complication rates compared to alternatives, metallic endoprosthesis have become the reconstruction option used most commonly. Complications and failures of these devices remain high compared to other arthroplasty procedures. Objective of this study was to retrospectively analyze results of a modular reconstructive tumour prosthesis for the lower limb (GMRS-Stryker) in primary and secondary implants.
**Materials and methods** Two hundred and ninety-five GMRS prostheses were implanted (2003–2010): 197 primary implants, 98 revisions in 84 failed primary reconstructions after tumour resection and 14 failed implants for non-oncologic reasons. Sites of reconstruction included: 199 distal femur, 60 proximal tibia, 32 proximal femur, 4 total femur. Histological diagnoses: 166 osteosarcomas, 22 Ewing sarcomas, 22 chondrosarcomas, 18 spindle cell sarcoma, 12 other sarcomas, 6 metastases, 35 giant cell tumours. Causes of endoprosthesis failure were classified as: soft tissues failures about the implant (type 1), aseptic loosening of the implant (type 2), structural fracture (type 3), infection (type 4), and tumour recurrence (type 5). Functional results (MSTS system) were analyzed and Kaplan–Meier curves of implant survival defined comparing primaries and revisions.
**Results** At a mean oncologic follow up of 4.2 years (range, 2 to 8 years), 195 patients are continuously NED, 43 NED after treatment of relapse, 10 AWD, 33 DWD. The overall failure rate in our series was 28.8 % and failure occurred at a median of 1.7 years (range, 1 month to 7 years). There was a significant difference in implant survival of all modes of failure between primary and revision implants ($p = 0.0313$). There was also significant difference in implant survival of failure of primary and revision proximal tibial implants ($p = 0.0410$). Breakage of prosthetic components did never occur. Functional scores were obtained in 229 of 295 patients. The average overall score was 81.6 % (24.5 range, 5–30).
**Discussion** Indications for the use of modular megaprosthesis are well defined in orthopaedic oncology, while their possible use in the non-oncologic setting is under investigation. Infection remain the most important complication.
**Conclusions** Middle term results with GMRS are promising, with excellent functional results and low incidence of complications. A significant difference in implant survival was found in this series between primary and revision implants. Functional results are satisfactory.

### Grade I chondrosarcoma: intralesional curettage versus wide resection: analysis of literature

M. Spinelli*[1], A. Piccioli[2]

[1]Ospedale Codivilla Putti (Cortina d’Ampezzo, IT);
[2]Policlinico Umberto I (Rome, IT)

**Introduction** Surgical treatment of intramedullary chondrosarcoma grade I is still debated. In the present study the authors have conducted a review of literature and a methanalysis of the data to analyze oncologic outcome in intralesional curettage versus wide resection in chondrosarcoma grade I.
**Materials and methods** A research has been made using MEDLINE, EMBAS and Cochrane Database. Cohort studies of patients with diagnosis of chondrosarcoma grade I who underwent intralesional curettage or wide margin resection were included. Two independent reviewers have selected the eligible studies with the Newcastle-Ottawa Quality Assessment Scale for Cohort Studies.
**Results** Five studies have been identified. These studies included, totally, 190 patients: 78 treated with intralesional curettage and 112 with wide margin resection. Just one case was localized in the pelvis and underwent a wide margin resection. In the cases analyzed there were 5 local recurrence and three cases of metastases. The risk of local recurrence and metastases did not differ significantly between the two group with a odd ratio for the intralesional curettage of 2.26 (confidence interval 95 %, 0.41–12.62) and 0.44 (confidence interval 95 %, 0.04–5.21) each.
**Conclusions** Actually there is no evidence in treatment of chondrosarcoma grade I. The analysis of literature evaluating the oncologic outcome (local recurrence and metastases) could achieve the goal to show the best surgical treatment for intramedullary chondrosarcoma grade I. The analysis have shown that intralesional curettage in extrapelvic localization of chondrosarcoma grade I do not increase the risk of local recurrence and metastases. Further analysis on a bigger number of patients are needed to have a more reliable data.

### ORAL COMMUNICATIONS

### *C09–BIOMATERIALS*

### Autologous collagen induced chondrogenesis technique (ACIC) for the treatment of chondral lesion of the talus: preliminary results

M. Cervellin*, C. Bait, E. Prospero, A. Quaglia, A. Redaelli, P. Volpi

Humanitas Clinical and Research Center (Rozzano, IT)

**Introduction** Chondral lesions greater than 2 $cm^2$ can be treated with microfractures in combination with chondral matrices. ACIC technique (autologous collagen induced chondrogenesis) uses swine atelocollagen and a stabilizer. The aim of the study is to evaluate the feasibility of the arthroscopic ACIC technique for the treatment of chondral lesions of the talus. We evaluated the cyto-compatibility in vitro by a matrix mainly constituted by a collagen gel (Cartifill) of bone marrow mesenchymal cells (BMSC) and chondrocytes (CH) of human origin.
**Materials and methods** We treated 6 chondral lesions of the talus with ACIC. All lesions were treated in a complete arthroscopic procedure with $CO_2$. We performed microfractures of the subchondral bone and then we covered the lesion area with Cartifill gel and finally we checked the stability. In vitro test: The BMSCs and CHs were isolated respectively from a sample of bone marrow and from fragments of articular cartilage and cultured in complete medium. Both cell populations were seeded on Cartifill at two different cell concentrations ($\times 10^5$ and 5.0 × 104 cells/scaffold). The seeding took place both before and after the final solidification induced by the addition of fibrinogen.
**Results** After 7 days of culture samples in vitro were assessed by histological analysis. The staining with hematoxylin-eosin revealed that both the BMSC and chondrocytes, when seeded at pre-solidification, colonized not only the surface but also the core of the sphere, remaining viable independently of the concentration of cells used. Instead the seeding at polymerization completed made the cells unable to migrate within the scaffold, growing only on the surface.
**Discussion** The ACIC technique has proved to be easy and feasible and can be used in a fully arthroscopic technique. The Cartifill is able to stably cover the lesion, forming a matrix of homogeneous coverage. The Cartifill is found to be cyto-compatible independently from the cell seeding density used. The seeding of BMSCs and CHs on Cartifill in pre-solidification is more advantageous because allows a more uniform cellular distribution than the one obtained on the scaffold already polymerized.
**Conclusions** The technique of micro-fractures, associated with the use of injectable matrices, could represent a significant advantage for its lower invasiveness in the treatment of grade IV chondral lesions of the talus.

### Trabecular titanium osteoinductive properties: a study on human adipose derived stem cells

L. Perticarini*, S.M.P. Rossi, A. Combi, F.M. Benazzo

Policlinico San Matteo (Pavia, IT)

**Introduction** Trabecular titanium is an innovative tridimensional, multiplanar, highly porous structure that imitates the morphology of trabecular bone with good mechanical properties. Adipose-derived stem cells (ASCs) are a mesenchymal multipotent cell population, that can be easily isolated from subcutaneous adipose tissue and can be induced to differentiate towards osteoblastic-like phenotypes. Is known from the literature that ASCs can differentiate in an osteoblastic-like phenotype when seeded on trabecular titanium scaffolds with osteogenic medium and extracellular matrix was also secreted.
**Materials and methods** ASCs isolated from subcutaneous adipose human tissue were seeded on trabecular titanium scaffolds (the structure has an average porosity of 65 %; the average diameter of the cell pores is 640 μm) and incubated with 5 % $CO_2$ at 37° C in growth medium and in osteogenic medium. Moreover ASC seeded on monolayer were cultured in three different media: growth medium, osteogenic medium and conditioned medium (medium collected from multiwell containing scaffolds in growth medium). The differentiation towards osteogenic phenotype of ASCs was evaluated by: I) qRT-PCR for expression of ALP and Runx-2 genes at time 0 and after 7 and 21 days from differentiation; II) biochemical and immunohistochemical assay for alkaline phosphatase and Runx-2 protein expression.
**Results** Trabecular titanium scaffold: after 7 and 21 days ALP and Runx-2 genes and protein were expressed by ASC in similar amount, independently by the medium used. Monolayer cultures: ALP and Runx-2 genes and protein were expressed by ASC grown in conditioned medium were similar to the one of ASC grown in osteogenic medium. ASCs were not differentiated towards osteogenic phenotype in monolayer growth medium.
**Discussion** In trabecular titanium scaffold ASCs can differentiate in an osteoblastic-like phenotype without the addition of osteogenic factors which are necessary for the differentiation of the same cells in monolayer. Further studies are needed to identify the molecular mechanism underlying the behaviour osteoinductive in vitro of trabecular titanium.
**Conclusions** The osteoinductive proprieties associated with those already demonstrated osteoconductive of trabecular titanium make this material useful in reconstructive surgery to fill bone defects that occur more often in revision hip replacement surgery.

### Pulsed plasma deposition of zirconia thin films on UHMWPE: proof of concept of a novel approach for joint prosthetic implants

M. Bianchi[1], N. Lopomo[2], A. Russo*[1], A. Ortolani[1], A. Visani[1], M. Boi[1], M.C. Maltarello[3], S. Sprio[4], M. Baracchi[1], M. Marcacci[5]

[1]Laboratorio NaBi, Istituto Ortopedico Rizzoli (Bologna, IT);
[2]Laboratorio di Biomeccanica e Innovazione Tecnologica, Istituto Ortopedico Rizzoli (Bologna, IT);
[3]Laboratorio di Biologia Cellulare Muscoloscheletrica, Istituto Ortopedico Rizzoli (Bologna, IT);
[4]ISTEC-CNR (Faenza, IT);
[5]Istituto Ortopedico Rizzoli (Bologna, IT)

**Introduction** Currently, the mean survival rate for total joint arthroplasty is ~90 % after 10 years; then, a revision surgery is generally required, due to osteolysis and aseptic loosening of the implant, which are strongly correlated with the formation of wear debris from the UHMWPE insert [Ingham, 2005]. Here, a new approach to overcome this detrimental issue is presented: hard, well-adhered and tough zirconia ($ZrO_2$) thin films directly deposited onto the surface of UHMWPE by the novel pulsed plasma deposition (PPD) technique in order to strongly limit the elasto-plastic deformation of the UHMWPE substrate especially under high local loads [Bianchi, 2013].
**Materials and methods** Three percent yttria-stabilized zirconia films were deposited by PPD technique. PPD is based on the ablation of a target material by a high-energy pulsed electron beam; the ablated material forms a plasma plume directed toward the substrate where it is deposited. Thank to the possibility to work efficiently even at room temperature, PPD is suitable for coating plastic materials. Films were characterized by SEM-EDX, X-ray diffraction, nano-indentation, adhesion and tribological tests. Moreover, the capability of the $ZrO_2$-UHMWPE system of carrying local loads—i.e. an estimation of the resistance to a third-body abrasion—was investigated.
**Results** Deposited zirconia films exhibited a fully cubic structure and a smooth nano-structured surface. Mechanical tests showed that hard, tough and well-adherent films were deposited. In particular, nano-indentation tests revealed rather high hardness and Young's modulus values (17 GPa and 154 GPa respectively), while critical fracture tests revealed that, even under loads as high as 500 mN (equivalent to $\sim$8 times the maximum pressure exercised on a femoral head during normal walking activity) no lateral cracks, spalling or pile-up phenomena were observable, revealing a high fracture toughness and a very high adhesion degree of the ceramic film to the plastic substrate. Moreover, preliminary tribological tests carried out in air against an alumina ball counterpart showed wear rate as low as $3.2 \times 10^{-6}$ $mm^3N^{-1}m^{-1}$ after 500000 cycles.
**Conclusions** The results showed in this work suggested the feasibility of pursuing this alternative and completely new route to improve UHMPWE performances while preserving its well-established mechanical properties.
**Suggested readings**

Bianchi (2013) J Mater Chem 1:310–318
Ingham (2005) Biomater 26:1271–1286

### The stromal vascular fraction: from a new strategy of cellular purification the possibility of new regenerative one-step applications in orthopaedic surgery

F.V. Sciarretta*

Clinica Nostra Signora della Mercede (Rome, IT)

**Introduction** Stromal vascular fraction (FVS) of adipose tissue consists of the connective support stroma for the adipocytes. It's a proven fact that it contains endothelial cells, preadipocytes, macrophages and mesenchymal stem cells. These stem cells from adipose tissue (ADSC) are present in a number 2500 times higher than that of the stem cells present in bone marrow. In fact, Suga et al. have shown that 1 g of adipose tissue contains $10.0 \pm 2.8 \times 10^5$ viable cells, of which 37–41 % are stem cells.
**Materials and methods** The FVS is commonly isolated by separation, collagenase digestion and filtration process of the share cells in order to obtain a cell suspension to be cultivated, which takes about 8–10 h and therefore of difficult clinical application. In 2009, Francis et al. studied an alternative protocol of rapid isolation of stem cells from the FVS without the use of collagenase digestion. On the basis of this new protocol and the ability to directly isolate mesenchymal stem cells from adipose tissue through their adherence to the cultivated area (Lattanzi et al.) was introduced on the market a system (MYStem Evo) particularly innovative that allows separating, washing and concentrating the cellular component on the basis of cell size directly inside a completely closed apparatus, without any centrifugation, with minimal handling and in absolute and total sterility, obtaining a final product enriched in multipotent stem cells ready for use and immediately in the operating room.
**Results** In this paper we present the rationale and the first experiences with the application of this innovative system for the treatment of cartilage knee defects and Achilles tendonitis.
**Discussion** Particularities of this technique are the ease of the aspiration procedure of abdominal adipose tissue through the use of a special biopsy blunt cannula, the chance to make cell culture, in a direct and secure way, in the operating room in few minutes, without any risk of contamination with time exactly matching that required for carrying out the one step repair process of the cartilage defect or tendinopathy.
**Conclusions** Based on this rationale and our initial experiences, we believe we can advise and propose to carry out controlled trials with autologous FVS in various orthopaedic pathologies of interest.

### Prospective evaluation of meniscal allograft transplantation

C. Terzaghi*[1], E. Borgo[2], C. Legnani[2], A. Ventura[2]

[1]U.O.S.D. Chirurgia Articolare Mininvasiva, Istituto Ortopedico G. Pini, Banca Regionale del Tessuto Muscoloscheletrico (Milan, IT); [2]U.O.S.D. Chirurgia Articolare Mininvasiva, Istituto Ortopedico G. Pini (Milan, IT)

**Introduction** The meniscus plays a crucial role in preserving and maintaining normal knee functions. Meniscal tears are common knee injuries that are responsible for pain and effusion in the joint. Whenever possible, meniscal tears are repaired. However, few meniscal injuries fulfil the criteria for adequate repair, thus meniscectomy is frequently performed. The absence of the meniscal tissue has been shown to result in articular cartilage degeneration. The goals of meniscal replacement are to reduce pain, to prevent degenerative joint changes and reduce the risk of osteoarthritis, and to restore optimally the mechanical properties of the knee joint after meniscal resection.
**Materials and methods** Between 2007 and 2010, five meniscus allograft transplantation were performed on patients with a history of lateral meniscectomy and knee pain and effusion at our institution. Clinical outcomes were evaluated over an average period of 3 years of postoperative follow-up by clinical assessment, Tegner score, Lysholm score, IKDC score, and MRI scan.
**Results** One patient underwent re-surgery due to detachment of the meniscus near the anterior horn. Inappropriate graft size has been identified as the factor responsible for the complication occurrence. In the remaining 4 cases (80 %), meniscal transplant reported reduction of the symptoms and improvement in knee function as demonstrated by IKDC and Lysholm scores ($p < 0.05$). MRI scan documented good integration of the allograft.
**Discussion** Several factors may influence the progression of degenerative changes in the knee joint following meniscal transplantation in the long-term: initial cartilage damage, surgical indications and technique, preservation and sterilization methods of the allograft, postoperative rehabilitation. Best results are reported in patients without advanced joint degeneration. Correct graft sizing is crucial for the procedure to be successful.
**Conclusions** Meniscal allograft transplantation represents a valid treatment option in patient which underwent meniscectomy. Correct indications, appropriate timing and careful graft choice are key-factors for a satisfying outcome.

### *C10–BIOTECHNOLOGIES 1*

### External fixation and platelet-rich plasma for the treatment of nonunions of long bones

M. Mariconda*, A. Cozzolino, S. Cerbasi, P. Recano, G.G. Costa

Dipartimento Ortopedia Università Federico II (Naples, IT)

**Introduction** Nonunion of long bones is a condition burdened by a high frequency of therapeutic failures. The aim of the present study was to evaluate the use of platelet gel supplementation to accelerate the healing of long bone nonunions treated by external fixation.
**Materials and methods** Twenty consecutive patients with atrophic tibial, humeral, ulnar, or radial nonunion treated with percutaneous stabilization with unilateral external fixator and injection of autologous platelet gel were followed prospectively. The healing time was compared to the results observed in a historical control group in which the external fixation was not supplemented with platelet gel. The rate of consolidation and time to healing in the two groups was evaluated in a blinded fashion by independent observers. The nonunion was judged to be healed when bridging callus formation on both radiographic views was observed on at least three of four cortices.
**Results** The cure rate of nonunions was 90 % in the patients treated with platelet gel (18/20) and 85 % in the controls (17/20) ($p = 0.633$). The mean time to radiographic healing in the group treated with platelet gel was 147 ± 63 days and no significant differences with the controls (153 ± 61 days) were appreciated ($p = 0.784$). Analyzing the mean healing time for separate segments, no differences were noted between study and control group (tibia: 112 ± 43 and 130 ± 5 days, respectively, $p = 0.382$; humerus: 225 ± 36 and 202 ± 70 days, respectively, $p = 0.530$).
**Discussion** There is a lack of literature data on the effectiveness of platelet gel and growth factors in the treatment of nonunions of long bones. The present study shows that the percutaneous injection platelet gel is not effective in promoting the healing of nonunions of long bones treated with external fixators.
**Conclusions** The present study failed to show the clinical usefulness of isolated percutaneous platelet gel supplementation in long bone nonunions treated by external fixation.

### Biotechnology in the treatment of delayed unions and non-unions of the femur

R. Erasmo, L. Guerra*, D. Palmieri

Unità Operativa Complessa di Ortopedia e Traumatologia, Presidio Ospedaliero Santo Spirito (Pescara, IT)

**Introduction** Delayed unions or non-unions after treatment of femur fractures derive from inadequate reduction and stabilization of the fracture, insufficient blood supply to broken bone, bone loss or inappropriate postoperative management. The treatment of this condition is always problematic and there is high incidence of complications.
**Materials and methods** We report the experience obtained from March 2010 to February 2013, in surgical treatment of femur fractures. We treated 10 patients, 7 with delayed union and 3 with clear signs of non-union of fracture. Treatment consists in new reduction and internal fixation, an autologous bone graft from controlateral femur with RIA (reamer/irrigator/aspirator) system, associated with the use of BMP-7 (bone morphogenetic protein-7).
**Results** Mean follow-up was 16 months. Patients underwent clinical and radiographic evaluation by periodic checks. Consolidation occurred in all patients, except one case of septic non-union. There were no complications in donor site of autologous graft. The only complication observed was a case of knee stiffness treated with mobilization in narcosis.
**Discussion** Delayed union or non-union of femur may be a consequence of inadequate treatment or post-operative management, but is often result of bone loss or avascular necrosis of fragments due to trauma's high energy. Therefore is important in treatment of this condition to ensure early mechanical stability associated to biological stimuli.
**Conclusions** Early treatment with the technique described above (new fixation with autologous bone graft and BMP) in our opinion shows clear advantages in determining the healing of these diseases, avoiding other problems to patients.

### Autologous concentrated bone marrow derived cells combined with dried bone allograft in long bones nonunions treatment

M. Scaglione*, L. Fabbri, D. Dell'Omo, F. Gambini, G. Guido

Clinica Ortopedica Università di Pisa (Pisa, IT)

**Introduction** Nowadays the treatment of long bone nonunions continues to be one of the most complex and debated topics due to the high number of failures. For several years in the relevant literature three factors have been considered essential in the healing process: growth factors and hormones, osteoprogenitor cells (mesenchymal stem cells, MSCs), extracellular matrix. The mechanical stability of the fracture site is considered the fourth element: diamond concept theory.
**Materials and methods** We dealt with 24 patients with long bones nonunion. All patients have been treated with concentrated mesenchymal stem cells without bone autologous transplant. We used the Extracell BMC-marrow aspirate protocol of Regen Lab. Four of our patients who had an infected nonunion (confirmed by MRI and scintigraphy) were treated by means of the Masquelet surgical technique. Other cases (non-infected nonunions) were treated employing the Judet technique. In both groups, the osteosynthesis was supported by apposition of demineralized bone (from the regional bone deposit) and autologous bone marrow derived cells harvested from the iliac crest. The radiographic parameters taken into account for the diagnosis of healing has occurred have been the presence of a bridge callus, obliteration of the fracture line and bone cortical continuity. Clinically, the pain was investigated with VAS score (visual analogue scale), where zero means no pain and 10 the worst possible pain.
**Results** Radiographic investigation shows complete healing in 78.9 % with a average time to healing of 6.5 months (minimum healing time 80 days) which has resulted even complete remission of clinical symptoms. The remaining 21 % still did not show signs of healing with radiographic checks.
**Discussion** Recently an interesting debate was opened about long bones PA treatment, but none particular technique demonstrated to be better than others. Tissue engineering has now the potential to dramatically change pseudoarthrosis therapeutic strategies thanks to the use of human stem cells.
**Conclusions** The application of autologous mesenchymal stem cells by the use of tissue regeneration's systems in orthopaedics is a valid and innovative biotechnology in PA treatment, finalized to accelerate healing process. Author's experience with this technique leads to complete healing in 79 % of cases. This result in PA can be considered satisfying though it's impossible to compare this result with other series, due to lacking literature of big surveys in this type of patients, in particular in healing time using or non-using biological adjuvants.

### Simulation of a rotator cuff repair through a finite element analysis

M. Mantovani*[1], L. Barbieri[1], M. Marini[1], P. Baudi[2], F. Catani[2]

[1]NCS-LAB Carpi (Carpi, IT);
[2]Azienda Ospedaliero-Universitaria Policlinico (Modena, IT)

**Introduction** In recent years several attempts have been made with 2D and 3D finite element models to simulate the humerus-supraspinatus system with the purpose of finding the peak stress areas and a correlation between tear position and correspondent lesion progression. Few attempts tried to reproduce cuff repairs.
**Materials and methods** Starting from the most significant published papers we created a 3D model in Ansys with the aim of reproducing the repair effects. The simulation makes use of a validated tendon constitutive model and moreover the acting loads as a function of position. Multiple lesions and correspondent repair configurations (suture bridge, double row, single row, transosseous and transosseous with an interposed device) can be compared as a function of bone and tendon properties. Further variables such as sutures number, span between stitches and number of anchors can be considered.
**Results** The transosseous and suture bridge repair provide a wider footprint coverage and an homogeneous pressure distribution which are between the most important factors influencing the biologic repair.
**Discussion** The various 3D repair models reproduction with finite element method represents a new approach in the biomechanical study background. This approach permits to evaluate and compare, in equivalent environments, essential factors such as contact area in the footprint, average pressure distribution, local stress peaks and the correspondent weak zones subjected to possible failures. These mechanical factors are directly correlated to the biologic factors affecting the healing process.
**Conclusions** The conceived 3D finite element repair model provide a flexible tool to compare different repair configurations by considering the organic environment properties. The measurement of geometric and mechanical parameters such as footprint extension, average contact pressure and peak stress can be used as a pre-operative assessing tool to consider the best repair approach. The transosseous and suture bridge configurations confirm the best measured properties.

### *C11–BIOTECHNOLOGIES 2*

### Regenerative medicine in treatment of osteonecrosis of the hip: morphogenetic proteins' role

P. De Biase*[1], O. Pecchioli[2], D.A. Campanacci[2], N. Mondanelli[2], M. Ippolito[2], R. Capanna[2]

[1]Azienda USL 8, PO San Donato (Arezzo, IT);
[2]AOU Careggi (Florence, IT)

**Introduction** Osteonecrosis of the hip is a disease that if left untreated evolves into a structural failure of the proximal and secondary osteoarthritis. In recent years in literature has been associated with foraging the infiltration of autologous concentrated bone marrow.
**Materials and methods** The aim of our study was to evaluate the effectiveness of the use of bone morphogenetic protein 7 (Osigraft) associated with core decompression in the treatment of osteonecrosis of the hip. From 2006 to 2010 we treated 23 patients (28 hips) with osteonecrosis of the proximal femur in Steinberg stage II. The core decompression was performed with dedicated instruments of Wright (X-Reamer) which create a tunnel of 9 mm in diameter in the femoral neck until the necrotic area which is subsequently removed with an expandable milling cutter. Subsequently the vacuum created was filled with a compound of autologous concentrated bone marrow and lyophilized tissue homologous or with an autologous concentrated bone marrow and Osigraft. The bone tunnel was then sealed with a compound of calcium phosphate (Prodense, Wright). We treated 11 patients with bone marrow concentrate and Osigraft and 16 cases with concentrated bone marrow and freeze-dried bone. The bilateral cases received only a vial of Osigraft that had not been used in the second treated hip.
**Results** At a minimum distance of 24 months we observed healing intended as the disappearance of symptoms and failure to progress of the lesion in 79 % of cases. We found a cure rate of 82 % healing in cases treated with Osigraft, a difference not statistically significant. The use of a scaffold in the compound injected into the area of necrosis is instead a significant result, since in all cases of failure no type of any scaffold had been used, but only the infiltration of bone marrow concentrate added or not to the Osigraft.
**Discussion** The use of concentrated autologous bone marrow is a valuable option with 79 % of success in treating early stage of hip osteonecrosis. Osigraft gives better results, but negative results were related to the lack of scaffold.
**Conclusions** The Osigraft improves the cure rate in core decompression and injection of concentrated bone marrow, but in bilateral cases it can only be used once. Our experience leads us to always recommend the use of a scaffold.

### Treatment of degenerative ankle osteochondral lesions using arthroscopy transplantantion of mononucleate medullary cells: clinical and radiological results

F. Castagnini*, R. Buda, M. Cavallo, A. Parma, L. Ramponi, F. Vannini, S. Giannini

Istituto Ortopedico Rizzoli (Bologna, IT)

**Introduction** Early degenerative ankle disease represents a challenging pathology, mostly in young people. Procedures like arthrodesis and ankle replacement are scarcely desirable. Other treatment, as mosaiplasty or autologous chondrocyte implantation, are difficult to be performed. Mesenchymal stem cells transplantation (BMDCT), successfully used for osteochondral lesions repair, due to their regenerative impact may be useful even in early joint degeneration, in order to delay or avoid more invasive procedures. Aim of this study is to report the clinical and radiological outcomes at short term follow-up of a series of patients who underwent one step technique for early degenerative ankle disease.
**Materials and methods** Between 2008 and 2011, 36 patients (mean age: 38.4 ± 7.1 years) affected by degenerative ankle disease grade I–II underwent arthroscopic debridement, impingement removal and BMDCT. Clinical and radiological evaluation encompassed AOFAS score, X-rays projections and MRI scans pre-op and at a mean follow-up of 26.7 ± 9.1 months. The bone marrow derived cells were aspirated from the posterior iliac crest, concentrated and implanted in the same surgical time (one-step).
**Results** AOFAS score improved from a preoperative of 41.86 ± 9.1 points to 77.50 ± 16.23 ($p < 0{,}0005$) at 2 year follow-up. A peak of 82.50 ± 9.05 points was recorded at 18 months. Negative prognostic features were the age of the patients, the dimensions of the lesions, the osteoarthritis degree and the time between the trauma and the treatment. Arthritis progression was evident in 17 % of the cases. MRI outcomes showed satisfactory parameters in term of lesions filling in 46 % of the lesions and partial restoration in 30 %. Two patients underwent ankle arthrodesis and other two patients reported low AOFAS results.
**Discussion** BMDCT, associated with the arthroscopic debridement, proved good, with satisfying clinical and radiological results. The degenerative process was arrested in many cases, achieving a partial or a total chondral and bony regeneration.
**Conclusions** BMDCT showed encouraging clinical and radiological outcomes in young and selected patients, emerging as a charming option to delay or even avoid arthroplasty or arthrodesis. Longer follow-ups and larger case series are needed to confirm these results.

### The treatment of early hip osteoarthritis with intra-articular ultrasound-guided injections of platelet rich plasma. A perspective study with short-term follow-up

C. Martini*, L. Nistri[1], B. Redl[1], L. Turelli[2], R. Civinini[1], M. Innocenti[1]

[1]CTO Firenze (Florence, IT);
[2]Ospedale S.M. Annunziata (Florence, IT)

**Introduction** Aim of this work was to evaluate the efficacy and safety of intra-articular injections of platelet rich plasma (PRP) in patients with early hip osteoarthritis.
**Materials and methods** Twenty-two patients with hip OA grade 1, 2 and 3 according to Croft's classification were included in the study. Twenty-two patients with hip OA grade 1, 2 and 3 according to Croft modified classification. They were 7 males and 15 females, the average age was 52.5 years. Each patient received three ultrasound-guided intra-articular injections of PRP, one every 3 weeks. The primary end point was the significant reduction in pain, described as a reduction in pain level of at least 30 % compared to baseline, as assessed by WOMAC scale evaluation at 6 months post-treatment. Moreover, we also used visual analogue scale (VAS) and the Harris hip score (HHS) for the evaluation of hip joint function and pain. Secondary endpoints included changes in the level of disability of at least 30 % and the percentage of positive responders, meaning the number of patients who achieved a reduction in pain and disability of more than 30 %.
**Results** All patients were available at the final follow-up. There were statistically significant reductions in VAS and WOMAC scale and in subscores of Harris hip score for pain and function at 8 weeks and 6 months ($p < 0.05$). Fifteen patients (68.1 %) reported a clinically important reduction in pain; in all cases they were patients with osteoarthritis of grade I and II as assessed by WOMAC scale. Sixteen (72.7 %) of these patients were classified as excellent responders, showing an early reduction in pain at 8 weeks, which was maintained at 6 months, and a contemporary reduction of disability. Side effects were minor and were limited to a feeling of heaviness in the injection site.
**Discussion** Among the possible options of the initial non-surgical treatment of hip osteoarthritis, in addition to infiltrative therapy with hyaluronic acid, one of the possible therapies is represented by infiltration with PRP. It was in fact demonstrated that platelet growth factors are able to determine a regenerative and reparative effect on articular chondrocytes and chondroblasts. For this reason we have studied the effect on early hip osteoarthritis.
**Conclusions** This preliminary uncontrolled prospective study assessed the safety, tolerability and the effectiveness of PRP intra-articular injections to relieve pain and improve function in a limited number of patients with early hip OA.

### *C12–SHOULDER AND ELBOW 1*

### Accelerated rehabilitation after rotator cuff repair: double-row technique reduces recurrence risk?

F. Franceschi[1], R. Papalia[1], E. Franceschetti[1], L. Diaz Balzani[1], S. Vasta*[1], N. Maffulli[2], V. Denaro[1]

[1]Campus Bio-Medico (Rome, IT);
[2]Centre for Sports and Exercise Medicine, Barts and The London School of Medicine and Dentistry, Mile End Hospital (London, UK)

**Introduction** Shoulder stiffness is the second most frequent complication affecting patients who underwent rotator cuff repair. Recently, an accelerated rehabilitation protocol it has been proposed to lower the risk of developing this condition. It includes closed-chaine exercises as well as passive overhead and passive external rotation movements to be performed during the first 6 weeks post-operatively in all patients but those who received a repair of the subscapularis tendon. This rehabilitative approach puts higher mechanical stress on the tendon-bone interface often causing failure of the repaired construct, and therefore the use of this special protocol has been limited only to those patients with high risk of postoperative stiffness. We hypothesize that, if accelerated rehabilitation is adopted, the fixation technique (single or double row) may affect the rate of postoperative failure of the repair.
**Materials and methods** We compared two groups of patients undergoing rotator cuff repair followed by accelerated postoperative rehabilitation. One group was treated with single row repair (SR group) while the other with double row repair (DR group). Sixty patients (30 per groups) were enrolled in our prospective randomized clinical trial. Inclusion criteria were (1) night pain; (2) no shoulder instability; (3) no fractures of the glenoid and of the greater and smaller tuberosity; (4) failure of conservative measures for a period of 6 months (NSAIDs, CCS intrarticular injections and physiotherapy); (5) loss of muscle strength. Shoulder MRI was performed at 6 months post-operation to assess the rate of re-tear. Clinical outcomes was evaluated measuring Constant-Murley and UCLA score.
**Results** Mean pre-operative UCLA values were 12 (4–23) for SR group and 13 (5–26) for DR group; at the final follow-up UCLA mean values were 31 (8–35) for SR group and 33 (11–38) for DR group, without significant intergroup differences. Re-tear occurred in 8 patients out of 30 in SR group and in 3 patients in DR group ($p < 0.005$).
**Discussion** Our results demonstrated as double row repair lead to a stronger tendon-bone interface, resulting in a significant lower re-tear rate. We are aware that clinical advantages of the double row repair are still troublesome compared to the single row, also considering the poor cost-benefits ratio of this technique.
**Conclusions** For these reasons we suggest to restrict the use of this repair technique only to patients presenting a high risk of shoulder stiffness which can benefit from accelerated rehabilitation.

### Arthroscopic posterior partial repair in irreparable rotator cuff tears: 3–9 year follow-up

M.C. Giordano*[1], S. Rosi[1], M. Villa[1], L. Castellani[2]

[1]Casa di cura Guarnieri (Rome, IT);
[2]Casa di cura Ulivella (Florence, IT)

**Introduction** The purpose of this study is to report the results of a particular rotator cuff tear pattern: (1) irreparable supraspinatus lesion despite tendon release; (2) infraspinatus tear (with or without delamination), reducible and repairable; (3) integrity of the subscapularis tendon; (4) long head of the biceps (LHB) variably involved. The procedure consisted in a partial posterior repair (PPR) of the infraspinatus in order to recreate a reliable transverse force couple.
**Materials and methods** From March 2004 to February 2010 we performed a PPR in 34 patients. There were 17 males and 17 females. The mean age at surgery was 61.0 years (range 46–72 years). The mean follow-up was 71.1 months. LHB tenotomy or tenodesis was performed if this tendon was damaged, unstable and/or a source of pain. In 8 cases the LHB was absent, in 14 cases a tenotomy was performed and in 12 cases a tenodesis was performed with 1 anchor. No interval slide mobilization was attempted. No subacromial decompression was performed. Clinical assessments were done before surgery and at final follow-up with UCLA score (0–35) and Constant score (0–100).
**Results** The UCLA score improved (29.8 *vs.* 10.5 $p < 0.01$) and was statistically significant, as well as the Constant score (33.9 *vs.* 74.7

$p < 0.01$). The best results have been reported for pain, motion and function. Constant score in group I (spontaneous LHB tenotomy), group II (surgical tenotomy) and group III (tenodesis) was not statistically significant (respectively 74.2 *vs.* 70.0 *vs.* 76.9, $p < 0.01$). There were no complications during surgery.

**Discussion** PPR is an effective procedure and patients improve their clinical condition, undergoing a less invasive surgery rather than a tendon transposition/transplantation or a reverse shoulder prosthesis and can be the best solution for dysfunction and pain in these selected cases.

**Conclusions** We consider the PPR an appropriate surgical approach in patients with a massive postero-superior rotator cuff tear, with intact subscapularis, without osteoarthritis and younger than 70 years. The different LHB treatments does not change the results.

### May endothelial dysfunction be a risk factor in rotator cuff tendinopathy?

R. Papalia*, L. Moro, E. Albo, S. Vasta, R. Antonelli Incalzi, V. Denaro

Università Campus Bio-Medico (Rome, IT)

**Introduction** Many factors are involved in the genesis of tendon lesions. Recent studies have recognized metabolic abnormalities as additional contributory factors in the development of rotator cuff (RC) tendinopathy. It has been hypothesized that the insertional area of this tendon is susceptible to degenerative changes due to intrinsic hypovascularization. The mechanisms underlying this process are yet not clear. In this study we attempted to confirm if larger lesions of the RC were related to impairment in the vasodilatatory response of the local circulation in conditions of hemodynamic stress.

**Materials and methods** We assumed that impaired vasal reaction to face hemodynamic stress was a systemic condition. This phenomenon should therefore be not limited to the critical area of the tendon tear. Given this assumption, post-ischemic vasodilation of brachial artery was studied through an echo Doppler evaluation. 50 patients (mean 61 ± 4, range 50–65), all scheduled for surgical rotator cuff repair following a tendon tear, were enrolled. We collected 3 preoperative measurements of the brachial artery diameter before and after application of an ischemic sleeve. The size of the lesions was later assessed at the time of surgery. We then proceeded to perform a statistical analysis of the eventual correlation between US assessment of brachial artery diameter and the corresponding size of the RC lesions. UCLA and ASES scores were also measured to assess clinical and functional outcomes.

**Results** Patients were classified into 4 groups according to Cofield's classification of tear size; respectively, 4 patients had massive lesions, 32 large, 10 medium and 4 had finally small lesions. The extent of the RC lesion showed to have inverse correlation with the diameter of brachial artery after an ischemic stimulus: an increase in size of the lesion corresponded to lower mean post-ischemic diameter of the vessel ($p < 0.0001$). UCLA and ASES data showed no statistically significant differences between the subgroups ($p > 0.534$).

**Discussion** It is not clear why the insertional area of tendons composing the RC is hypovascularized. We hypothesized there is an imbalance between local vasodilator and vasoconstrictor factors. The prevalence of vasoconstrictor substances determines a reduced post-ischemic vasodilation. The data presented provide the basis for the future identification of vascular impairment that could underlie the beginning of tendon degeneration in patients that are not yet affected by injury.

**Conclusions** An imbalance between vasodilator and vasoconstrictor factors could be the basis for vascular distress of RC. More specific vascular patho-physiological studies are however needed to better understand this mechanism.

### Resurfacing arthroplasty in arthritis of the shoulder

E. Siuni*[1], A. Mastellone[1], D. Nonne[2], G.L. De Pascalis[2], B. Orrù[1]

[1]Servizio Chirurgia della Spalla, SCOMR, ASL Cagliari (Cagliari, IT); [2]Scuola di Specializzazione Ortopedia (Cagliari, IT)

**Introduction** The aim of this study is to review long-term results of resurfacing arthroplasty of the humeral head in shoulder's pain caused by avascular necrosis, arthritis, rheumatoid arthritis.

**Materials and methods** From 2006 to 2011 we treated 21 patients, 9 male and 12 female; the age was about 49 and 71 years. All patients undergone standard shoulder X-rays and MR study (someone CT scan) before surgical treatment. Thirteen patients had a degenerative arthritis, 2 had rheumatoid arthritis of the shoulder, 4 had avascular necrosis and 2 had post-traumatic arthritis. All of this was valued using the Constant-Murrey score, standard X-rays and occasionally ultrasonography to evaluate the rotator cuff. The average follow-up was 32 months (15–72).

**Results** In all patients we had an increase of Constant score and it was above 76 after surgical treatment. Less levels of Constant score was in subjects with rotator cuff lesions too (lesion of the supraspinatus tendon); we treated this one in the same time. One of all was dissatisfied because feels persistence of a residual pain and functional limitation compared with against side. At X-rays we did not find evidence of radiolucent lines, no signs of primary loosening.

**Discussion** The indications for the shoulder resurfacing is primary concentric osteoarthritis, post-traumatic osteoarthritis, rheumatoid arthritis, avascular necrosis, non-healing fractures associated or not with lesions of the rotator cuff. The shoulder resurfacing saves the bone stock of proximal humerus; it reduces the peri-operative blood loss and it get early hospital discharge. Contraindications are local or systemic infections, pseudoarthrosis, neurological and muscular disorders, instability, osteoporosis or less bone stock.

**Conclusions** The resurfacing shoulder arthroplasty is a well alternative to stemmed prosthesis because it respects the normal anatomy and geometry of the shoulder joint with minimal bone resection and provides good functional recovery with marked reduction of pain.

### *C13–SHOULDER AND ELBOW 2*

### Factors influencing internal rotation recovery in reverse prosthesis

N. Ivaldo, G. Caione, M. Rossoni

Acqui Terme, IT

**Introduction** The purpose of the study is to evaluate the influence of the subscapularis tendon and the humeral stem version on internal rotation recovery in patients with reverse prosthesis.

**Materials and methods** We clinically evaluated a series of 93 non-consecutive reverse arthroplasty (CTA Delta and Delta Xtend) with mean follow-up of 38 months with the aim of analyzing the influence of the subscapularis and the humeral stem version on internal rotation recovery. The evaluation was effected pre-and intra-operatively and on the final follow-up. The series includes patients with eccentric arthropathy or uncompensated rotator cuff massive lesion. The second inclusion criterion was the presence of an effective teres minor.

**Results** Patients with the worst preoperative active elevation (EAA less than 60°) obtained the best value to the final check (158°), probably because in this group there was a significant percentage of rotator cuff massive lesions without osteoarthritis, therefore without important rigidity. A second interesting fact is that, comparing patients in which the subscapularis was still intact or at least 2/3 and the group in which this muscle was totally torn, the internal rotation was identical (5.3 *versus* 5.2 points evaluated with the Constan score). The third important finding was that patients in which the humeral stem had been implanted with 0° of version obtained a modest but significant gain on the internal rotation (average 5.6 points corresponding to L4), with respect to those in which the stem had been implanted with a retroversion of 20° (average 4.2 points corresponding to the level of sacrum). Since March 2010 we have begun to implant a series of prosthetic stems with 10° of anteversion in order to improve the internal rotation recovery. Only 11 patients (11.8 %) with mean follow-up of 24 months was included in this study. Internal rotation recovery was identical to patients with stem implanted at 0° (5.6 pts evaluated with Constan score).

**Conclusions** Subscapularis function in the reverse prosthesis remains to be clarified. In our study its presence did not affect the internal rotation. We showed a difference of 2 vertebral segments on the internal rotation recovery in patients with stem implanted at 0° compared to patients with stem implanted at 20° of retroversion. No difference on the internal rotation has occurred by placing the stem at 10° of anteversion with respect to that obtained with 0° of version.

### Results of stemless-metaphyseal reversed shoulder tess prosthesis in excentred osteoarthritis

M. Stamilla*[1], P. Teissier[2], J. Teissier[2], T. Gaudin[2]

[1]Catania, IT;
[2]Montpellier, FR

**Introduction** Reverse shoulder arthoplasty (RSA) developed by Grammont is a treatment that improves pain, mobility and function in very poor conditions, combining glenohumeral arthropathy and cuff deficiency. The aim of this study was to evaluate the efficiency and the performance of stemless-metaphyseal reversed shoulder TESS prosthesis.

**Materials and methods** We report a prospective series of 105 reverse shoulder arthroplasty. This is homogeneous, continues and monocentric series with a minimum of 2 years follow-up. The used implants are always the same: the TESS RSA with metal back glenoid compound, reverse head, reverse corolla, optional stem and PE insert. The group of the patients was regularly revised clinically and radiologically. Clinical evaluation based on VAS scale, ROM and Costant's score. Radiographic examination allowed to measure a glenoid inclination angle, humeral metaphysis angle, glenoid tilt and scapular notching signs.

**Results** There were 87 patients (91 cases), the mean age at surgery was 73 years. Post operative pain was 2.9/15 *versus* 7.44/15; the mean active anterior elevation was 143° *versus* 95°, the gain in external rotation with elbow at the side was 39° *versus* 26°, the abduction was 139° *versus* 89°. The absolute Costant-Murley score increased from 40.5 to 68.4 points. The X-rays showed, the mean GM angle was 48.9°. The BMI was significantly correlated with the preoperative GM angle and with the notching risk. Scapular notching was observed in according with Sirveaux classification in 71 % stage 1 and in 28 % stage 2. The GH mean angle was 153.9°.

**Discussion** Study shows that the results with shoulder TESS prosthesis are comparable to the data of the literature. The development of notching on the pillar of the scapula following RSA is a frequent complication; in our study the rate of notching was 62.7 % (71 % grade 1, 28 % grade 2). The 93.4 % of TESS-RC didn't move. The problems relating to the stem was: periprosthetic fractures, shoulder arthroplasties revisions and anatomical variation and modularity. The TESS-RSA improves: reliability of implants, the humeral bone stock, decreased peri-prosthetic fractures risk, best adaptation to anatomical variation, choice of RC position (CD: 135°, 150°,155°).

**Conclusions** The stemless implant allows for easier revision as there is no need for stem removal. In the stemless prosthesis, an osteotomy of the head is performed, allowing for optimal visualization of the socket as in conventional stemmed implants. We recommend so the pose of this type of RSA prosthesis in case of high quality bone stock.

### Changes in positioning of the glenosphere and effects of the lateralization the rotation centre of the reverse shoulder prosthesis

L. Nalbone*[1], M. D'Arienzo[1], R. Adelfio[1], T. Ingrassia[2], V. Nigrelli[2], G. Trinca[2], P. Paladini[3], F. Campi[3], G. Merolla[3], G. Porcellini[3]

[1]Clinica Ortopedica e Traumatologica, Policlinico "Paolo Giaccone", Università degli Studi (Palermo, IT);
[2]Dipartimento di Ingegneria Chimica, Gestionale, Informatica, Meccanica, Università degli Studi di Palermo (Palermo, IT);
[3]Laboratorio di Biomeccanica, Ospedale Cervesi di Cattolica (Cattolica, IT)

**Introduction** The reverse shoulder prosthesis has revolutionized the treatment of cuff arthropathy. Through the medialization of the rotation centre of the gleno-humeral joint, the tension of the deltoid is heightened, increasing the abduction and alleviating pain. Scapular notching, instability and limitation of rotation of the limb are the major problems. The aim of our studies is to determine the effects obtained through modifications in the implant technique of the glenoid component and the biomechanical changes produced by the lateralization of the rotation centre.

**Materials and methods** With the use of a 3D scanner, the computer created a skeleton of the shoulder and a design of a reverse prosthesis, using the Tornier prosthesis. The prosthesis was then mounted and assembled in the shoulder. It was implemented in two configurations: in the first the glenoid component was implanted implementing a standard milling and in the second the milling was obtained by performing a cut inclined at 15° with respect to the plane perpendicular to the transverse and 30° with respect to front to obtain changes in the gleno-humeral kinematics. Then a spacer was positioned between the glenosphere and the base of the glenoid implant to obtain the lateralization of the rotation centre and study the changes in the movement of abduction and intra-external rotation and in both cases, and to minimize scapular notching.

**Results** When there is a cut at 15° there is a decrease in the abduction, and with the limb adducted and elbow flexed to 90°, there is a decrease in internal rotation with an increase in external rotation. Joint stability was not affected by the changes. The insertion of a spacer, between the glenoid and the glenosphere, in both configurations, with modifications in the cut, resulted in an improvement of in all ranges of joint abduction of over 110°.

**Discussion** The inverse prosthesis medializes the rotation centre and moves the humerus down. It decreases the pressure forces on the glenoid and increases the tension of the deltoid that allows the abduction of the limb. After surgery, however, residual limitation of rotation remains because of poor excursion of the humeral cup around the glenosphere. The lateral displacement of the rotation centre causes an increase in pressure forces on the glenoid and increases the tension of the deltoid, but it improves joint stability by decreasing the cutting forces, influencing the movements of rotation and decreasing the risk of scapular notching.
**Conclusions** The change of the geometry of the reverse prosthesis allows the shoulder paralyzed by cuff injury to move again. The displacement of the rotation centre and the muscular retensioning always remain the key of the new joint system.

### Treatment gleno-humeral arthropathy using a biological graft

F. Vannini*, G. Pagliazzi, V. Persiani, A. Shehu, S. Giannini

Istituto Ortopedico Rizzoli (Bologna, IT)

**Introduction** Surgical treatment of severe post-traumatic shoulder arthritis in young patient is a challenge. Bipolar fresh osteochondral allograft (BFOA) is a fascinating option for patients without severe cuff tear arthropathy.
**Materials and methods** Three patients of mean age 48.5 ± 0.7 years, affected by post-traumatic unilateral arthritis of shoulder, received BFOA. The ideal patient to allograft match was permitted through CT scans and X-rays. Patients evaluation was carried out clinically with the Constant score and radiographically with X-rays, CT scans and MRI. In order to limit the degenerative changes, patients were advised to post-operative soft immunosuppressive therapy for 6 months. Bioptic specimens were harvested during hardware removal at 12 months follow-up in all the patients for histological and immuno-histochemical evaluation.
**Results** The Constant score improved from 38.3 ± 2.9 points pre-operatively to 78.7 ± 16.2 points at 12 months of follow-up, to 72.3 ± 15.3 at 24 months and to 59.3 ± 22 at final follow-up of 34.6 ± 4 months. The average improvement at the final follow-up was 32.3 ± 39.8 points. One patient was not satisfied with the procedure, due to instability and muscle strength deficiency, but allograft revision had not been performed. Good radiographic consolidation of the graft was observed at 5 months. Mild arthritis was evident at the final follow-up, without a correlation with clinical outcomes. Bioptic specimens showed cartilaginous structure with an high proteoglycan content and a normal cellular distribution, collagen type II expression. Analysis of synovial biopsies demonstrated limited number of macrophages, without prominent perivascular inflammatory cell infiltrates or lymphoid aggregates.
**Discussion** For properly selected patients without symptomatic and disabling rotator cuff deficiency, BFOA can result in improvements in pain, motion, function, and patient satisfaction.
**Conclusions** The preliminary results of this technique are promising, but radiographic arthritis at follow-up, was evident although immunosuppressive therapy, correct positioning and size of the graft. This finding, even if uncorrelated to the clinical results, still remain cause of concern. Further research regarding the immunological behaviour of transplanted cartilage are needed in order to improve the results.

### *C14–SHOULDER AND ELBOW 3*

### Fractures of the humeral proximal epiphysis: surgical treatment using plates with angular stability

G. Restuccia*, A. Grimaldi, V. Gargano

Ortopedia (Catania, IT)

**Introduction** Fractures of the humeral proximal epiphysis are relatively frequent and are still growing because of an increase of mean age of the population as they are related to osteoporotic diseases. They represent the 4–5 % of the whole number of fractures. They appear because of high energy trauma in young individuals and because of low energy ones in old patients. The male sex is the most struck by this kind of pathology. The traumatic mechanisms, besides the direct ones, are a combination of torsion forces and bending of the superior limb.
**Materials and methods** The authors examined the clinical records of 350 individuals who have been treated from 2005 to 2011, concentrating on plate osteosynthesis (Neer 2–3). The majority of the patients are males (60 %) of a range age between 60 and 75 years (mean age 67.5), mainly struck on their right shoulder (55). The results have been estimated through clinical and radiological follow-up at 1 month and 3 months, using the Constant score.
**Results** The average follow-up is 3.5 years (range 1–7). All of the fractures showing an average consolidation time of 5 weeks, with no signs of joint fragments necrosis. We achieved a 73.3 % of good and excellent outcomes in line with the ones already reported in literature. Clinical results have been optimal, achieving an almost complete recovery of joint functionality of the shoulder with an average Constant score of 86.3 points.
**Discussion** Despite the remarkable incidence of this kind of lesion, it doesn't exist a common mindset concerning its treatment in literature. During the last two decades the use of ORIF technique is spreading thanks to the technique refinement and the improvement of materials, leading it to be an optimal surgical solution in the treatment of the complex proximal humeral fractures.
**Conclusions** Modern plates with angular stability, which have been created and drawn for the fractures of proximal humerus, have surely led to an improvement in functional results among the most complex fractures. The use of this kind of plates has always allowed to a stable fixation of fractures, permitting a early mobility and a faster return of the patients to their daily activities.

### Surgical treatment of traumatic ruptures of the pectoris major

G. Merolla[1], L. Nalbone*[2], G. Porcellini[1], P. Bianchi[3]

[1]U.O. Chirurgia Spalla e Gomito, AUSL Rimini (Cattolica, IT); [2]U.O. Ortopedia e Traumatologia, Policlinico Giaccone (Palermo, IT); [3]Dipartimento di Scienze Ortopediche, Traumatologiche, Riabilitative e Plastico-Ricostruttive della Seconda Università degli Studi di Napoli (Naples, IT)

**Introduction** Rupture of the pectoralis major is an uncommon lesion first described in 1822 by Patissier. The lesions are classified according to the degree (partial and complete) or location (tendon, muscle–tendon junction, intramuscular). Ruptures are reported in young athletes with high muscle performance due to the anomalous contraction of the muscle–tendon unit. The mechanism most likely detrimental in the elderly is an abrupt movement applied to a stiff and atrophic muscle. The lesions usually affect the sternal component.
**Materials and methods** Complete ruptures require surgical treatment because they determine loss of strength in adduction, flexion and internal rotation. In this study, we report the results of direct repair after acute injury in 5 cases and 2 chronic cases treated by reconstruction with autologous fascia lata. In all patients treated we used a deltoid-pectoral surgical approach, reinserting the tendon in its anatomic location with two metallic anchors. After surgery, the patients were immobilized for 30 days before starting physiotherapy. Active rehabilitation was begun after 90 days.
**Results** The results at a mean follow-up of 48 months were excellent in all cases treated in an acute phase, with satisfactory strength recovery and return to previous activity. The two cases treated by reconstruction with fascia lata have had good functional recovery but a lower recovery of strength.

**Discussion** Traumatic ruptures of the pectoralis major mainly affect the sternal component both for the particular anatomy of the muscle and for the mechanism of the injury. An early surgical approach is suggested in acute injuries but reconstruction with an organic tendon graft can provide adequate functional recovery.

**Conclusions** Injuries of the pectoralis major are rarely observed and the diagnosis and early treatment ensure a better functional prognosis. Reconstruction with allograft offers good prospects for recovery in chronic cases.

### Intra-medullary nail in proximal humerus fractures: our experience and considerations at a mean follow-up of 32 months

S. Creaco*[1], P. Ditto[1], G. Topa[2], V. Burgio[1], M.A. Rosa[1]

[1]Scuola di Specializzazione in Ortopedia e Traumatologia, Dipartimento di Scienze Biomediche e delle Immagini Morfologiche e Funzionali, Sezione di Ortopedia e Traumatologia, Università degli Studi di Messina (Messina, IT);
[2]Azienda Ospedaliera Bianchi Melacrino Morelli, Ospedale Riuniti (Reggio Calabria, IT)

**Introduction** We report clinical and radiographic follow-up (medium 2.8 years; range 1–5) of 75 patients having proximal humerus fractures operated with intra-medullary nail.

**Materials and methods** The authors present a retrospective study on a series of 75 consecutive patients operated with intramedullary nail (PHN or UHN) for proximal humerus fractures between January 2008 and January 2013. We used Neer and AO classifications to stage the fractures including A2–B3 and B1–B3 types (AO classes). 47 patients were females and 28 males with a mean age at surgery of 68 years (range 26–91 years). The aims of the surgical treatment consisted of reduction of the immobility time and fast mobilization of the shoulder. We evaluated the time to fracture healing, pain intensity (by VAS), shoulder function (in accordance with Neer criteria), ROM, patient satisfaction and post-operative complications.

**Results** In 90 % of cases the fractures healed without complications and clinical consequences. Subjective satisfaction was excellent/good in 85 % of cases, fair/adequate in 10 % and poor in 5 %. The main causes of failure were: misplacement of the distal screws (3 cases); pull-out of the spiral blade (2 cases); subacromial impingement (1 case); rotator cuff injury (2 cases).

**Discussion** To obtain a functional recovery is necessary early identification of the lesion and perform the right surgery. It needs to move quickly the shoulder to avoid post-traumatic stiffness. The intramedullary nail gives a primary stability for rapid mobilization.

**Conclusions** Intra-medullary nailing is a good choice to stabilize fractures of the proximal humerus with 2–3 fragments, mainly in osteoporotic patients. The nails promotes early mobilization to reduce post-traumatic stiffness and accelerates functional recovery. The respect of indications and the performing of a right technique are required for the best results. In osteoporotic patients is useful to employ a system that increases the bone contact surface.

### Treatment of humeral diaphyseal nonunions with PRP and MSC

V. Triolo*, R.A. La China, S. Mannino, M. D'Arienzo, N. Galvano

U.O. Ortopedia e Traumatologia Policlinico (Palermo, IT)

**Introduction** Diaphysis fractures of humerus, as the majority of these kinds of fractures, are at risk of developing nonunion. This complication is favoured by the presence of local risk factors (kind of fracture, exposure) and general factors (cigarette smoke, alcohol, diabetes, vascular diseases). Treatment should aim to increase the mechanical stability of the system and induce the formation of bone callus, stimulating osteoinductive, osteoconductive and osteogenetic activities.

**Materials and methods** From November 2011 to January 2013 at our U.O. were treated 12 cases of patients with humeral diafyseal nonunion: 8 of fracture previously treated with intramedullary nail, 4 of fracture treated with plate and screws. Our protocol consists of two phases: the first involves the taking of two test-tubes with sodium citrate of 8 cc of blood, subsequent centrifugation and preparation of the platelet gel; the second phase involves the withdrawal of MSC (mesenchymal stem cell) from the iliac crest of the patient and subsequent centrifugation. Following surgery for removal of the means of synthesis, removal of the inert tissue, approach of the two bone stumps with filling of the gap by means of a compound obtained from the mixture of PRC, MSC of bone marrow and bone substitute. Fixation either with plate and screws or intra-medullary nail, preceding reaming of the medullar canal.

**Results** Twelve patients were subjected to clinical and radiographic controls up to 6 months. In 9 cases we obtained a clinical cure and formation of the bone callus at 6 months. In 3 patients, in which we observed minimal formation of the bone callus, it was necessary to resort to magnetotherapy cycles.

**Discussion** Clinical controls and X-ray showed signs of bone callus formation, reduction of pain in the previous fracture, good clinical and functional recovery of the affected limb in the majority of patients treated. These data are attested by the recent literature.

**Conclusions** Our protocol has shown positive results in the treatment of the humeral diafyseal nonunions because works by stimulating the three basic elements of bone regeneration: osteogenesis (mesenchymal stem cells, MSC), osteoconduction (bone substitute), osteoinduction (PRC).

### Reverse shoulder arthroplasty for proximal humeral fractures: results after implantation without subscapularis repair

F.A. Grassi*, I. Zorzolo, M.N. Molina, P. Fornara

Università del Piemonte Orientale, A.O.U. "Maggiore della Carità" (Novara, IT)

**Introduction** The use of reverse shoulder arthroplasty for the treatment of proximal humeral fractures in the elderly population has been proposed to overcome the limited efficacy of traditional fixation techniques and anatomic replacement in these patients. The aim of this study is to report the results observed in a consecutive series of 19 patients, in whom the reverse arthroplasty was performed according to a standardized technique, without subscapularis repair.

**Materials and methods** Between 2009 and 2012, 19 reverse prostheses with a cemented stem were implanted in 19 patients affected by complex fractures of the proximal humerus. A concomitant rotator cuff tear was present in 9 patients. All the patients were women, with an average age of 75 years (70–83) at the time of injury. The surgical procedures were performed through a delto-pectoral approach. In all the cases the humeral component was implanted in 20° of retroversion, with peri-prosthetic reconstruction of the greater tuberosity and external rotators; the lesser tuberosity and subscapularis were not repaired. Passive motion was started on the first post-operative day; after one month the patients were allowed to use the operated shoulder for activities of daily living.

**Results** No complications occurred in the post-operative period. At an average follow-up of 20 months (8–46), 15 patients were evaluated clinically and radiographically; one patient was interviewed by phone, while three patients were untraceable. Average shoulder active elevation was 108° (50°–160°). Pain was absent in 11 patients, mild/moderate in 4 and severe in one patient. The mean absolute Constant-Murley score was 46 (16–69), while the normalized score based on gender and age was 59 (24–91); the average SPADI (Shoulder Pain and Disability Index) score was 39 (6–89).
**Discussion** Functional recovery of the shoulder after a proximal humeral fracture is a difficult goal to reach in the elderly population. Like other surgical options, reverse arthroplasty does not lead to good results in all the treated patients and a wide variability among individual outcomes can be observed. In our experience, subscapularis repair was not a critical factor for prosthesis stability; future studies should clarify its possible relevance on the functional result.
**Conclusions** Reverse arthroplasty is a reliable surgical technique for the treatment of traumatic injuries of the proximal humerus and finds its main indications in elderly patients. Potential drawbacks of the operation on general conditions, compliance to rehabilitation and functional needs should be individually and carefully evaluated before recommending this surgical option.

### *C15–SHOULDER AND ELBOW 4*

### Outcomes of surgical treatment of the terrible triad of the elbow: prospective study on 22 patients and review of the literature

G. Giannicola*, M. Scacchi, F.M. Sacchetti, D. Polimanti

Università "La Sapienza" (Rome, IT)

**Introduction** Our aim was to analyze the clinical and radiographic results of 22 patients affected by terrible triad and to compare our results with the literature. We hypothesised that satisfactory results can be achieved in the majority of cases by applying a rigorous diagnostic-therapeutic protocol.
**Materials and methods** Twenty-two patients with a mean age of 52 years were considered. Preoperatively, X-ray and CT-scan with 2D–3D reconstructions were performed. The surgical procedure adopted for radial head fractures consisted of the reduction and internal fixation with screws or a plate, while a radial head prosthesis was implanted in non-synthesizable fractures. Internal reduction of the coronoid fractures was performed in 21 of the 22 cases by means of transosseous sutures (9 cases) and/or fixation using the fragment fixation system (FFS) (12 cases), depending on the number and size of the fragments. Moreover, capsule-ligament reconstruction and, in persistent joint instability, the application of a hinged elbow fixator were performed. The functional evaluation was assessed with MEPS, DASH-score and m-ASES. Literature review was performed with the PubMed database.
**Results** The mean follow-up was 26 months. The average MEPS was 91, with results being excellent in 15 cases, good in 5 and poor in 2; the average DASH-score and m-ASES were 9.2 and 85.9, respectively. The mean flexion, extension, pronation and supination were 137°, 13°, 80° and 77°, respectively. One patient was re-operated on for elbow stiffness, with excellent clinical results at the last follow-up. We identified 9 comparable studies published in the last 10 years. The rate of unsatisfactory results and re-operations in these studies, in which a total of 215 patients were treated, was 0–56 % and 0–29 %, respectively.
**Discussion** The terrible triad is challenging to treat and has a history of complicated outcomes. The primary goal of surgical fixation is to obtain a stable joint that permits early motion. The advances in elbow knowledge combined with improved implants and surgical techniques have led to the development of standard surgical protocols. The application of this standard surgical protocol allowed us to obtain satisfactory results in 91 % of cases. Results were unsatisfactory in the remaining 9 % of cases, with a reoperation being necessary in one case (5 %). These results are comparable to those reported in the literature. Patients should be informed of the rate of unsatisfactory results and complications that may require additional surgical procedures.
**Conclusions** A standard management of the terrible triad reduces the rate of unsatisfactory results and complications.

### Kinematic analysis of functional movements in patients with total elbow replacement: biomechanical study with computer analysis system of movement

G. Giannicola*, F.M. Sacchetti, S. Stigliano, S. Coco, F. Alviti, M. Mangone, M. Paoloni

Università "La Sapienza" (Rome, IT)

**Introduction** The elbow represents the link between the shoulder and the hand, allowing both the orientation of the latter in space and many activities of daily living (ADL). Pathologies that compromise elbow function impede the execution of these activities, causing a marked worsening in the quality of life. Some of these pathologies can be treated with a total elbow replacement (TER). In recent decades, TER has evolved considerably as a result of a more thorough knowledge of the anatomy and biomechanics. The aim of the study was to analyze the kinematics of the replaced elbow during the execution of common daily activities.
**Materials and methods** Eight patients who underwent a TER were evaluated (mean age: 62.8 years; mean time after surgery: 24.6 months). The discovery elbow system (DES) was implanted in all the patients by the same surgeon. A optoelectronic system (ELITE, BTS) was used to carry out a kinematic evaluation while the patients were performing different ADL (reading a book, drinking, eating, pouring a drink, combing hair, using the phone). This system was also used to assess the ROM of the implanted elbow and the contra-lateral elbow.
**Results** The mean values of the flexion–extension arc and pronation-supination arc obtained in each task (implanted elbow *vs* non-implanted elbow) were: reading a book = 73° *vs* 37° of flexion–extension and 134° *vs* 123° of pronation-supination; drinking = 103° *vs* 83° of flexion–extension and 60° *vs* 55° of pronation-supination; eating = 79° *vs* 64° of flexion–extension and 80° *vs* 101° of pronation-supination; pouring a drink = 58° *vs* 79° of flexion–extension and 65° *vs* 55° of pronation-supination; comb hair = 79° *vs* 79° of flexion–extension and 99° *vs* 83° of pronation-supination; using the phone = 100° *vs* 118° of flexion–extension and 132° *vs* 113° of pronation-supination.
**Discussion** The analysis of elbow kinematics showed that the ROM of the replaced elbow is comparable to that of the contralateral limb during the performance of ADL, and also allows the execution of functional tasks that often require a greater functional ROM than that reported in the literature (130-30 of flexion–extension, 50-50 of pronation-supination).
**Conclusions** This study shows that TER is a viable solution for the treatment of different pathologies of the elbow and, if correctly implanted, allows all ADL to be performed thanks to the preservation of the physiological elbow kinematics and biomechanics. Moreover, this study highlights the need to increase the current values of functional ROM, as they are often insufficient for the performance of modern ADL.

### Our experience in the use of prosthetic in the healing of the radial head and elbow fractures

R. Lupo, S.A. Rapisarda*, G. Palmisciano

U.O. Ortopedia, Ospedale S. Giovanni di Dio (Agrigento, IT)

**Introduction** The treatment of fractures of the elbow remains a challenge for the surgeon both for it's difficult balance of the injury, as in the technical difficulties of repair. Given the importance of the radial head as a secondary stabilizer for the elbow, joint replacement results a fundamental option, especially in the presence of comminuted fractures or associated injuries of the ligaments. The purpose of this work is to analyse the clinical results obtained in a series of patients with fractures of Mason III-IV of the radial head.
**Materials and methods** The case study concerns 35 patients in which 25 are males and 10 are females, with an average age of 43 years old who have undergone surgical treatment of radial head arthroplasty in our division from April 2005 to April 2012. We used 17 bipolar prosthesis to press fit, 3 bi-articular long stem cemented prosthesis, 15 monoblock and press fit prosthesis. In 14 cases the fractures were of Mason type III, in 21 cases of Mason type IV. In 19 cases there were associated injuries 5 fractures of coranoid, 3 fractures of the proximal ulna, 8 cases of injuries of the LCL (lateral collateral ligament) and 3 MCL (ulnar collateral ligament). In 10 patients an articulated external fixator was applied to protect the repair which was kept in place for about 5 weeks.
**Results** The average follow-up was of about 32.2 months (range 10–63) in accordance with the MEPS score of an average of 80 points, we have had excellent results in 15 patients, good results in 12 patients, mediocre in 6 patients, and poor results in 3 patients. The Dash score was of an average result of 11.5 points. What complications we had: 8 cases of heterotropic bone, 2 cases of mobilization that required the monoblock press fit and this required a review, 3 cases of early osteoarthritis, 1 case of wound dehiscence and 3 cases of rigidity.
**Discussion** Prosthetic replacement of the radial head represent an essential condition for the restoration of the elbow stability in the presence of comminuted fractures or associated ligament injuries. Our survey covers only patients treated in acute and with failure of osteosynthesis or resection and the functional result agree satisfactorily with the other authors.
**Conclusions** Based on our experience we can say that the prosthetic replacement of the radial head shows encouraging results, despite being aware of the difficulties, in terms of the indications and the correct surgical technique.

### Radial head fractures. Surgical treatment options: resection, reconstruction or prosthesis?

G. Caruso*[1], A. Aquino[1], F. Del Prete[1], L. Preziuso[2], A. Vitali[2], A. Petrini[1]

[1]SC Ortopedia e Traumatologia Ospedale San Giovanni Di Dio (Florence, IT);
[2]SS Chirurgia Mano e Arto Superiore, Ospedale di Palasciano (Palasciano, IT)

**Introduction** Radial head fractures represent 20 % of elbow injuries and are typically treated conservatively. Traditional criteria for surgical indication are: joint extension, patient's age and comminution. Osteosynthesis has been so far reserved to younger patients with non-comminuted fractures, while in more complex cases capitellum resection or debridement of the fractured fragments are performed. In recent years, the treatment of fractures of the radial head and associated ligamentous injuries of the elbow has included alternative techniques (new generation radial head prosthesis, osteosynthesis and ligament reconstruction techniques). Today, the treatment choice does not consider the radial head alone, but evaluates the overall stability of the elbow. Non-operative treatment still retains its value only for compound fractures with an otherwise stable elbow. We use the Mason classification (osteosynthesis indicated for some fractures of type I and type II; prosthetic replacement for some type III and type IV).
**Materials and methods** From January 2006 to December 2012 we treated 179 fractures of the radial head, 40 of these (22.3 %) operatively (23 females, 17 males, age range 13–90 years). In 1 case the fracture was exposed; in 9 it presented with an acute elbow dislocation, in 2 the dislocation was impacted. In 10 cases there was an associated ulnar fracture. Of the 40 operative cases, we performed 20 osteosynthesis, 11 radial head prostheses replacement, 5 total and 4 partial resections. In 21 cases we associated a ligament reconstruction. In 3 cases of severe instability we applied a temporary external fixator.
**Results** Patients were assessed at follow-up with DASH and MEPI procedures. We obtained excellent results in 25 cases, fair in 10, sufficient in 3 (1 case osteosynthesis with stiffness, 2 cases of revision surgery for capitellum mobilization), poor in 2 (1 osteosynthesis complicated by algodystrophy and stiffness: we removed the plate and performed arthrotomy after 6 months; one case of partial resection required an elbow hemiarthroplasty).
**Conclusions** Treatment of fractures of the radial head has evolved in recent years. Whenever possible, radial head osteosynthesis should be performed. Otherwise, a radial head prosthesis should be performed; in selected cases, capitellum resection is still a viable alternative. New knowledge on the biomechanics of the elbow made us change our approach mandating greater emphasis to anatomical reconstruction. Correct diagnosis is today crucial; fractures of the radial head must be considered not an isolated event, but in the broader context of functional anatomy of the entire elbow.

### *C16–FOOT AND ANKLE 1*

### Lateral ligament reconstruction with allograft for the treatment of chronic ankle instability

C. Legnani*[1], C. Terzaghi[2], E. Borgo[1], A. Ventura[1]

[1]U.O.S.D. Chirurgia Articolare Mininvasiva, Istituto Ortopedico G. Pini (Milan, IT);
[2]U.O.S.D. Chirurgia Articolare Mininvasiva, Istituto Ortopedico G. Pini, Banca Regionale del Tessuto Muscolo Scheletrico (Milan, IT)

**Introduction** Chronic ankle instability is a condition of perception of giving way and persistent pain usually following multiple ankle sprains. In case of severe joint laxity, surgical treatment with reconstruction of the lateral ligamental complex is recommended. The aim of this study was to evaluate the functional outcome of patients treated with lateral ligament reconstruction with allograft for severe chronic lateral ankle instability.
**Materials and methods** We performed a retrospective cohort study of 10 patients who underwent allograft external ligamentoplasty for severe chronic lateral ankle instability between 2009 and 2011, with an average follow-up of 16.3 months (SD: 8.2). Median age at surgery was 29 years (range: 25–35). All patients presented both ATFL and CFL lesion. Patients were evaluated using AOFAS score, Karlsson-Peterson score, Tegner activity level, Sefton stability scale, and objective examination comprehending ROM, anterior drawer sign and talar tilt test. Telos Stress equipment was used for pre- and post-operative radiographic laxity testing.

**Results** Follow-up examination at an average of 16.3 months (SD: 8.2) after surgery showed significant improvement of all variables compared to pre-operative values ($p < 0.001$). Most patients rated their outcome as good/excellent. Telos Stress radiographs documented improvement in joint stability.
**Discussion** Many treatment modalities have been proposed for the treatment of chronic lateral instability. Many papers support anatomical repair as the ideal treatment option in the treatment of chronic ankle instability. However, according to several surgeons, ligamentoplasty possesses more stability compared to anatomical repair and frequently no adequate ligamentous structures are available for a direct repair. In order to reduce donor site morbidity, some authors advocated the use of allografts, which possess advantages such as shorter operative time, no donor site morbidity, better availability of grafts, decreased incidence of arthrofibrosis and fewer postoperative complications. Disadvantages include risk of disease transmission, the potential for subclinical immune response and increased cost. The results of the present study demonstrate that allograft ligamentoplasty represent a reliable treatment option in patients with combined ATFL and CFL lesion.
**Conclusions** Lateral ligament reconstruction with allograft represents a valid treatment option in patients with severe chronic lateral ankle instability and leads to satisfying clinical outcomes and reduced donor site morbidity.

### Distal tibiofibular syndesmosis reconstruction using the Arthrex Tightrope System

P. De Santis*, P.P. Rota, G. Gualtieri, A. Rota

Ospedale S. Pertini (Rome, IT)

**Introduction** The tibiofibular syndesmosis complex lesions require rapid diagnosis and early treatment to avoid unfavourable long-term results. Many treatment are described in literature. We present many cases using the Arthrex Tightrope System for the management of tibiofibular syndesmosis lesions.
**Materials and methods** Twenty-four patients with rupture of the distal tibiofibular articulation are treated with Arthrex Tightrope System. Associated fractures are treated with AO technique.
**Results** Mean follow-up is 15 months. The post-operative radiographic analysis included the average distance from the tibial plafond Tightrope, the average postoperative tibiofibular overlap, the overlap average tibiofibular after 15 months.
**Discussion** The Tightrope use in syndesmosis diastases is a good solution. The operative technique is fast, easy, minimally invasive and avoids the implant removal.
**Conclusions** In our series, has maintained an excellent reduction of the syndesmosis.

### Anatomical tenodesis for the treatment of chronic lateral ankle instability: a new surgical technique

F. Morelli*, M. Guidi, A. Ferretti

Ospedale Sant'Andrea (Rome, IT)

**Introduction** Ankle sprains are among the most common injuries in sports and everyday life. In about 20 % of grade III ankle sprains, a residual laxity is still present and it may require a surgical treatment. Numerous surgical techniques are described for the treatment of chronic ankle instability. They can be divided into two categories: anatomical ligamentous reconstructions and non-anatomical techniques with the use of autologous or allograft tendons. In our department, an innovative surgical technique was performed for the stabilization of chronic instability of the ankle. This procedure consists of a tenodesis with one-half of peroneus brevis tendon (or gracilis tendon), through the reconstruction of the natural course of the anterior talofibular ligament and calcaneo-fibular ligament.
**Materials and methods** Between May 2011 and April 2012 five patients underwent a lateral ankle tenodesis (4 with one-half of peroneus brevis tendon and one with gracilis tendon). All patients had pre-operative positive ligamentous laxity test. The neo-ligament was fixed to the calcaneus bone with a mini-Swing-Bridge (Citieffe), to the lateral malleolus through a bone tunnel and to the talar neck with a metal anchor. Radiographic evaluation was performed with standard radiographs, stress-radiographs pre- and post-operatively and at one-year follow-up. AOFAS score (ankle, hindfoot score) and the FADI index (foot & ankle disability index) were assessed for the evaluation of the ankle functionality.
**Results** The results at one-year follow-up were satisfactory in all patients, with an improvement of AOFAS score (pre-operative: 18, post-operative: 93) and FADI index (pre-operative 49, post-operative 95.2). Stress radiographs showed a significant reduction of both the talar tilt (side to side mean pre-operative: 27.5, post-operative 5.1) and the anterior drawer test (s/s 29.7 average pre-operative, post-operative 12-1).
**Discussion** In severe chronic ankle instability, the quality of the ligamentous remnants does not always guarantee sufficient mechanical strength. In these cases, non-anatomical techniques are preferred (e.g. modified Watson-Jones procedure) to the detriment of the normal anatomy of the lateral ligament complex of the ankle.
**Conclusions** This new surgical technique allows to combine the advantages of the conventional tenodesis to the biomechanics preservation of the anatomical reconstruction. Despite a small group of cases, all patients had satisfactory results at one-year follow-up.

### Retrospective study on intra-articular injections with HYMOVIS in haemophilic patients with ankle arthropathy

C. Ruosi*[1], S. Liccardo[1], D. Rossi[1], G. Colella[1], S. Lupoli[1], A. Coppola[2], M.N.D. Di Minno[2]

[1]Dipartimento di Ortopedia e Traumatologia, Università "Federico II" (Naples, IT);
[2]Centro di Riferimento Regionale per i disordini della coagulazione, Dipartimento di Medicina Clinica e Sperimentale, Università "Federico II" (Naples, IT)

**Introduction** Haemophilic arthropathy is the consequence of recurrent joint bleeds in patients affected by severe haemophilia A or B or Von Willebrand disease, leading to functional limitations and chronic pain, with negative impact on quality of life. Main target joints are knees, ankles and elbows. Few therapeutic options are available in ankle arthropathy. Temporary symptom improvement have been reported by repeated (3–5) intra-articular administrations of hyaluronic acid (HA).
**Materials and methods** Patients with ankle arthropathy undergoing a single HYMOVIS (Fidia) injection were retrospectively studied. HYMOVIS is a sterile, non-pyrogenic, hydrogel manufactured with HYADD 4 (hexadecylamide of highly purified natural sodium hyaluronate obtained by bacterial fermentation). The hyaluronic acid's chains have a molecular weight of 500–730 kD. The small chemical modification introduced allows the formation of hydrophilic and hydrophobic interactions which are able to increase the rheological parameters of the product (viscosity and elasticity). Clinical assessment, including Gilbert Score (pain—PGS, and physical examination—PEGS), pain scores (visual analogue scale—VAS, and McGill Score—MGS) and generic quality of life evaluation (EQ5D), and magnetic resonance imaging (EMIS score) were performed at baseline (T0) and 1 (T1), 6 (T6) and 12 (T12) months after HYMOVIS injection.
**Results** Twelve ankles in 7 patients (5 severe, 2 moderate, mean age 34, 5 years) were treated. A significant improvement of symptoms and functional outcome was reported at T1 *vs.* T0 (PGS $0.5 \pm 0.4$ *vs.* $1.5 \pm 1.0$, $p = 0.03$; PEGS $2.4 \pm 2.1$ *vs.* $4.8 \pm 3.5$, $p = 0.001$; VAS $17 \pm 16$ *vs.* $74 \pm 27$; MGS $0.9 \pm 0.7$ *vs.* $2.9 \pm 1.2$, $p < 0.001$; EQ5D $83 \pm 15$ *vs.* $55 \pm 25$, $p = 0.015$; EMIS $15 \pm 1$ *vs.* $15 \pm 1$, $p < 0.05$).
**Discussion** Data were not statistically different at T6 *vs.* T1, suggesting long-term benefits after HYMOVIS injection. Only 2 patients presently reached T12. The positive experienced outcomes led to repeat treatment in these 2 patients, 15 months (mean) after first injection. No significant change in the European Magnetic Resonance Imaging Score was found throughout the study.
**Conclusions** Treatment with HYMOVIS has allowed the improvement of joint mobility by increasing the viscoelasticity of the synovial fluid, with consequent improvement in the quality of life of patients and clinical benefits which have proved to be long lasting. This single-injection treatment provides an approach reducing risks and costs of multiple intra-articular injections, well accepted by patients and with a favourable cost-utility ratio.

### *C17–FOOT AND ANKLE 2*

### Reconstructive surgery in sequelae of ankle fractures

M. Mosca*, F. Vannini, M. Di Liddo, A. Shehu, S. Giannini

Istituto Ortopedico Rizzoli (Bologna, IT)

**Introduction** Many factors are related to long-term complications in sequelae of ankle fractures like the degree of the joint damage, the severity of the fracture and the quality of the reduction. Numerous clinical reports demonstrate that the articular anatomy must be restored to achieve good results and to avoid pain, osteoarthritis, deformity and stiffness. This is a review of our experience in the treatment of malunion and arthritis of ankle fractures using reconstructive surgery instead of arthrodesis, with a peri-articular release, malleolar osteotomies, and an articular graft repair.
**Materials and methods** The case report consisted of 52 cases, 30 males and 22 females, mean age 43 years, treated from 5 months to 2.8 years after injury and followed up after an average of 6 years. The surgical technique consisted of osteotomies, combined or not, with soft-tissue release, grafting of autologous mesenchymal cells to repair cartilage defects. The post-operative treatment consisted of early CPM gradual weight-bearing after 4 weeks and complete weight-bearing allowed after an average of 12 weeks. Clinical and radiographic evaluation of the results was performed.
**Results** Twenty-one patients were considered excellent: normal range of motion, no pain, and no progression of the arthritis. Twenty patients were considered good: slight pain after long walk, range of motion limited less than 15°, and no progression of the arthritis. Five patients were considered fair with decreased range of motion, slight pain, and progression of arthritis, and four of these were poor because of persistent severe pain, stiffness and deformity due to severe arthritis, and persistent subluxation of the talus. In these cases we performed a subsequent arthrodesis.
**Discussion** If the arthritis is not severe at the time of surgery, and if the joint congruity is achieved with the operation, results of the late reconstruction after ankle fractures are excellent and good in more than 80 % of the cases, with no severe pain, despite a slight reduction of range of motion and regardless of the severity of the defects.
**Conclusions** Late reconstruction is also indicated in cases of severe arthritis in young patients, with the aim to delay an ankle fusion, allograft or replacement with biological prosthesis.

### External fixation *versus* ORIF with plate and screws for the treatment of sanders type 2 and 3 calcaneal fractures: our experience

G. Bruno*, D. Ghargozloo, L. Cannavò, A. Raffa, M. Riccioli, L. Costarella, S. Avondo

Catania, IT

**Introduction** The calcaneus is the most fractured tarsal bone (2 % of all fractures). This kind of fractures are common in young/adult males following car accidents or work injuries. Calcaneal fractures can be intra or extra-articular with different patterns. CT scan is mandatory in order to plan the adequate treatment. Our aim is make a comparison between two different surgical techniques: external fixation and ORIF with plate and screws.
**Materials and methods** Between January 2009 and January 2013 at the Division of Orthopaedic Surgery and Traumatology of the University of Catania, 47 patients underwent to a surgical treatment for a calcaneal fracture. Thirty patients were males (64 %), 17 females (36 %) with a mean age of 41 age (range 20–67). All fractures were studied with an X-ray exam (antero-posterior, lateral and axial) and with a CT scan with coronal reconstruction, performed at the ER. Sanders classification of calcaneal fractures has been followed. Twenty fractures were Sanders type 2, 27 were type 3. Thirty-two fractures were treated with external fixation, 15 with ORIF.
**Results** Patients were clinically evaluated every week for the first month and every two weeks for the following months and with an X-ray follow-up at 1, 2, 3, 6 and 12 months, then every year. Mean follow-up was 10 months (3–18 months). Fracture healing was observed in 43 cases, the other 4 showed a incomplete consolidation at the last follow-up. A clinical evaluation with the MFS score were performed. MFS score improved from 70 (average) at one month follow up to 88.0 (average) at the last follow–up.
**Discussion** Both techniques showed good results. At the X-ray exam, ORIF offers better images but the clinical results are comparable. ORIF disadvantages are: wound complications, higher deep infections rate, another surgical incision for remove the plate, damage of soft tissues. External fixation disadvantages are: lower patients compliance, pin infections, difficulties in the reduction of fractures.
**Conclusions** There isn't a consensus on the best way to treat a calcaneal fracture. The surgeon have to consider a number of factors: severity of the fracture, soft tissues involvement, age, compliance and activity level of the patient, smoke, diabetes. Both methods showed good results in Sanders type 2 and 3 fractures but with specific differences and indications.

### Distal-tibia epiphyseal detachment in paediatric patients

L. Marciandi*, A. Pascazio, P.V. Frediani

Ospedale S. Antonio Abate (Gallarate, IT)

**Introduction** Distal-tibia epiphyseal detachments represent from 25 % to 38 % of all growth-related cartilage injuries. Around 60 % of these detachments take place during sport activity. They are more frequent in males between 8 and 15 years of age. They are anatomically classified according to Salter. Typical transitional-age triplanar fractures are pointed out, as well as Tillaux fracture.
**Materials and methods** Our study has been a multi-centre one, in order to gather data from a broad survey. Therapies are analyzed and most common complications are identified.
**Results** Five-hundred and one cases of tibia epiphyseal detachment have been revised, with an average follow-up of 18 months. We have established predominance of male sex, sport-related traumas, as well as incidence of Salter 1–2, 3–4, 5, triplane, Tillaux. Types of treatment and complications have been analyzed.
**Discussion** Starting from a non-homogeneous survey with a limited follow-up in view of an effective appraisal of late complications (growth arrest, angular defects), we have obtained epidemiologic data which are in line with literature, and have identified treatment guidelines. Plaster for simple fractures, open surgery and synthesis for displaced fractures (in line with literature).
**Conclusions** It is a frequent pathology, and not a complication-free one. Anatomical reduction is of major importance for treatment.

### Balloon assisted reduction, pin fixation and tricalcium phosphate augmentation in calcanear fracture: our first 10 cases

D. Vittore, G. Caizzi*, G. Vicenti, V. Conserva, M. Dilonardo, A. Abate, B. Moretti

Unità Operativa di Ortopedia e Traumatologia II, Azienda Ospedaliera Universitaria Policlinico di Bari (Bari, IT)

**Introduction** The goal of treatment for intra-articular calcanear fractures has been focused for years on the anatomical reduction of the articular surface; open reduction and internal fixation are the most widely used techniques, but they are associated with high rate of wound breakdown and infection. We propose a new technique of reduction using an inflatable bone tamp filled with tricalcium phosphate.
**Materials and methods** We randomized 10 patients with Sanders III and IV calcanear fracture. Patients underwent surgery at a mean of 4.6 days (range 2–10 days), necessary for the resolution of soft tissues pain and oedema. We positioned a calcaneus traction wire to correct misalignment. Fluoroscopy was used to determine the quality of calcaneal alignment and of fracture reduction. A stylet and a cannula were placed into the calcaneus followed by insertion of a bone tamp attached to a digital manometer. The balloon was inflated gradually under fluoroscopy. Bone cement was prepared immediately prior to its injection into the defect and the balloon was removed. The patient was able to walk with partial weight-bearing at the removal of K-wires (7th day). Full weight bearing was performed after 4 weeks. Clinical follow-up was performed at 1, 2, 3, 6 and 12 months postoperatively, using the Maryland foot score. MDCT scan was obtained for the evaluation of the cement position, the reduction of joint surface and the calcaneal alignment at 3 months after surgery.
**Results** Mean operative time was 40 min, without any significant blood loss. Seven patients showed immediate pain relief; 3 patients reported pain when mobilizing the subtalar joint. Two patients had a more difficult reduction for the longer time passed since the trauma occurred (9 and 10 days). The mean follow-up was 8.4 months (at least 6 months, max. 12 months). Both the immediate postoperative radiographs and MDCT at 3 months showed a good reduction of the launch and recovery of the volume heel with satisfactory lifting of the talar articular surface. Five patients resulted excellent, 4 good and 1 sufficient at Maryland foot score. No infection was reported.
**Discussion** Restoration of correct orientation of posterior calcanear articular surface, restoration of length, width and axial angle of valgus, early restoration of mechanical stability of calcaneus appear the main parameters to be reconstituted in the case of complex articular calcanear fractures.
**Conclusions** This method allows both early mobilization and protected load.

### Autologous chondrocyte implantation in talar osteochondral lesions: results after a 7-year follow-up and predictive value of T2 mapping

G. Pagliazzi*, R. Buda, F. Vannini, M. Cavallo, A. Olivieri, S. Maraldi, S. Giannini

Istituto Ortopedico Rizzoli (Bologna, IT)

**Introduction** Autologous chondrocyte implantation (ACI) in the ankle is a consolidated procedure. Aim of this study was to report the clinical and qualitative results of a series of patients who underwent ACI of the talus at 7-years follow-up.
**Materials and methods** Forty-six patients (age $31.4 \pm 15.1$ years) affected by talar osteochondral lesion received ACI. Chondrocyte were loaded into hyaluronic acid scaffold and arthroscopically implanted. Patients were evaluated by AOFAS and MRI pre-operatively and at intervals up to $87.2 \pm 14.5$-months follow-up. MRI qualitative T2 mapping was performed in 20 patients at 5 years.
**Results** AOFAS improved from $57.2 \pm 14.3$ (pre-operative) to $86.8 \pm 13.4$ (12 months), to $89.5 \pm 13.4$ (36 months), up to $92.0 \pm 11.2$ a final follow-up of $87.2 \pm 14.5$ months ($p = 0.0005$ from baseline to each follow-up). Three cases failed and histological and immuno-histochemical evaluation was performed on regenerated tissue harvested during revision showing fibro-cartilaginous tissue along with tissue in remodelling phase and presence of type II collagen expression. T2 mapping detected regenerated tissue with T2 map value in mean $69 \pm 21.6$ % of the repaired lesion areas compatible with hyaline (35–45 ms) and direct correlation with maintenance of clinical score at final follow-up ($p < 0.005$).
**Discussion** ACI in talar osteochondral lesions showed satisfactory clinical results improving over time. The quality of the regenerate, detected by T2-mapping had an impact on the durability of the clinical result. This confirm the capability of MRI T2-mapping to give a reliable qualitative evaluation of the repair tissue.
**Conclusions** The treatment of talar osteochondral lesions with the implantation of autologous chondrocytes is confirmed to be a reliable technique that can provide stable results over time. The MRI T2-mapping method showing effective benefit both in the evaluation of cartilage repair tissue and also representing a non-invasive to the second-look arthroscopy.

### *C18–FOOT AND ANKLE 3*

### Surgical techniques for the treatment of diabetic Charcot foot with external fixation

A. Kirienko*[1], A. Peccati[1], G. Sacchi[2], R. De Giglio[2], N.M.A. Portinaro[3]

[1]Istituto Clinico Humanitas (Rozzano, IT);
[2]Unità Piede Diabetico, Ospedale di Abbiategrasso (Abbiategrasso, IT);
[3]Clinica Ortopedica, Università degli Studi di Milano (Milan, IT)

**Introduction** Charcot arthropathy is a disabling pathology of the foot and the ankle, which requires an effective treatment to improve clinical and functional outcomes and prevent foot amputation. The aim of the study is to present our experience with the correction of the foot and the restore of its function in diabetic patients with Charcot deformities using the principles of the Ilizarov technique and circular external fixation.

**Materials and methods** Between 2010 and 2012 we treated 13 patients (7 males and 6 females, meanly aged 48.27 years; 1 patient affected by type 1 diabetes and 12 patients by type 2 diabetes) with different kind of deformities of the mid- and hind-foot (4 patients with arthropathy and dislocation at the level of the Lisfranc joint, 5 patients with Chopart joint dislocation, 1 patient with a combined pathology of the Lisfranc and the talo-navicular joints, 1 patient with pathology of the Lisfranc and the subtalar joints, 1 patient with necrosis of the talar body, 1 patient with osteomyelitis and amputation of the 4th and the 5th rays with associated rigid equino-varus deformity). Seven patients had plantar skin ulcerations because of articular instability and overload at the plantar arch.

**Results** All the patients have been treated with open resection, partial intra-surgery reduction, frame application and progressive correction of the residual deformities. In the case with necrosis of the talus a tibio-talar arthrodesis was performed, while in the case with equino-varus deformity after the initial deformity correction a panarthrodesis was performed using the same frame. All the skin ulcerations healed during the treatment. The mean follow-up time has been 34 months.

**Discussion** With the Ilizarov technique we achieved the restore of the foot morphology, with a plantigrade foot in all cases, and no deep infection or recurrent skin ulcerations; no amputations were required. This technique is best reserved for patients who are at the highest risk for complications or have failed with standard orthopaedic methods of internal fixation.

**Conclusions** After our experience, we recommend one step surgical technique of dynamic external fixation for the treatment of Charcot deformities in the diabetic foot, with progressive correction of associated residual deformities.

### Correction of complex foot deformities with V-shaped and Y-shaped osteotomies of the tarsal bones and circular external fixator

A. Kirienko*[1], A. Peccati[1], E. Arnaldi[1], N.M.A. Portinaro[2]

[1]Istituto Clinico Humanitas (Rozzano, IT);
[2]Clinica Ortopedica, Università degli Studi di Milano (Milan, IT)

**Introduction** Complex deformities of the foot can be due to several causes (trauma, poliomyelitis, osteomyelitis, neuro-muscular conditions, congenital deformities) and present different components on different spatial planes. Children can be early treated with soft tissues procedures; in adults such approaches are ineffective and bony procedures are required—such as osteotomies of the talus and the calcaneus.

**Materials and methods** Between 2000 and 2011, we treated 55 feet (28 right- and 27 left-side) belonging to 50 patients (33 males and 17 females, meanly aged 29.91 + 14.51). Fourteen patients were affected by clubfoot, 13 by other congenital deformities, 15 by deformities due to neuropathies, 8 by post-traumatic deformities. We performed 36 V-shaped and 19 Y-shaped osteotomies.

**Results** In all cases the normal anatomy of the foot was restored in order to permit a correct and pain-free gait. The mean time-in-frame was 15.42 + 8.03 weeks, and the mean follow-up time has been 6.47 + 4.68 years. The main complications during treatment were local infection (15 cases), Kirschner wires breakage (10 cases), soft tissues linear necrosis (7 cases), early consolidation of the bone regenerate (4 cases), neuro-vascular lesions (1 case of A/V shunt).

**Discussion** Many procedures are available for correction of complex foot deformities, but they require an invasive approach and present a variable rate of complications. The Ilizarov method permits a progressive correction of this kind of foot deformities in all the spatial planes. Both the V-shaped and the Y-shaped osteotomies permit the independent correction of the deformities of the hind- and the mid-foot, but the latter prevents an excessive lengthening of the foot and requires a shorter time for consolidation.

**Conclusions** The Ilizarov technique, together with a tarsal osteotomy, permits the correction of complex deformities of the foot in those patients for whom a conservative approach is not possible, maintaining the gained outcome even in the long-term. On the other side this technique requires a long learning curve.

### The treatment of claw toes deformity in lesser toes: new surgical technique extensor digitorum longus pro brevis

M. Pompili*, F. Calderazzi, B. Pedrabissi, E. Dalpane, F. Ceccarelli

Azienda Ospedaliera Universitaria Parma, Ospedale Maggiore, Clinica Ortopedica (Parma, IT)

**Introduction** The aim of the technique is to preserve as far as possible the muscle–tendon units of the forefoot and to be easy for the surgeon to contrive.

**Materials and methods** This new and innovative technique includes the elongation of the lesser toes extensor apparatus through the suture between the proximal end of the extensor digitorum longus tendon (cut above the proximal phalanx on its distal part) and the distal end of the extensor digitorum brevis tendon (cut on its proximal part). Sixty-six patients underwent this technique: in most of them (97 %) the claw toes deformity was associated with other foot deformities (hallux valgus 90 %, 2 % hollow foot, flat foot 5 %). Rigid claw toe deformities were mostly found in the second ray (more than 90 %), 65 % in the third ray, 35 % in the fourth ray, 2 % in the fifth ray of our cases. In cases such as these was performed an additional arthrodesis of the proximal interphalangeal joint, then fixed with a temporary Kirschner wire. In dynamic deformities was worth an additional manipulation into forced hyperextension, then fixed with a temporary Kirschner wire (3 % in the second ray, 33 % in the third ray, 48 % in the fourth ray, 25 % in the fifth ray). The new technique and a manipulation were performed in the remaining cases.

**Results** We conducted a retrospective study on fifty-four subjects at an average follow-up of 34.5 months (11–65 months). The analyses included: physical examination, paper pull-out test, measurement of flexion strength (N) of lesser toes, AOFAS score, VASFA, SF-36. Excellent results and remarkable patient satisfaction were reported for 98 % of all cases.

**Discussion** We observed a perfect recovery of the forefoot morphology and the regression of the functional metatarsal overload (calluses). The flexion strength has achieved the 50th percentile of normative data. The scores of the tests express a consistency between patient satisfaction and performance measured.

**Conclusions** The extensor digitorum longus pro brevis technique is an effective procedure to correct claw toes deformity.

### Bioreabsorbable implants for flatfoot surgical treatment

V. Persiani*, P. Capra, D. Luciani, C. Pungetti, C. Faldini, S. Giannini

Istituto Ortopedico Rizzoli (Bologna, IT)

**Introduction** Flatfoot represent one the most common orthopaedic disorders in childhood and its treatment can be surgical or non-surgical. If surgery is indicated, that means functional flatfoot, sub-talar arthrorisis with bioreabsorbable or non bioreabsorbable implants is the treatment of choice. Aim of this study is to evaluate and compare the results obtained using two different bioreabsorbable screws designed for subtalar athroreisis in childhood.

**Materials and methods** From September 2010 to September 2011, 60 patient (120 feet), 34 male and 26 female, mean age 10.8 years (range 9–12), affected by bilateral flexible flatfoot underwent bilateral arthrorisis with bioreabsorbable implants. If needed, Achilles tendon lengthening, posterior tibial tendon re-tensioning, hallux valgus correction, other fingers correction, were performed. After surgery, walking plaster casts were positioned for 2 weeks in case of arthroereisis only, for 5 weeks (walking admitted after two weeks) in case of arthroereisis combined with other procedures. All patient were evaluated before and after surgery using the same protocol consisting of clinical and radiographic evaluation and Gait Analysis.

**Results** Mean follow-up was 18 month (range 12–24). Arthrorisis were performed in 60 cases with bioreabsorbable endorthesis and in 60 cases using bioreabsorbable calcaneostop. In 23 cases arthrorisis were combined with additional surgical procedure: 14 Achilles tendon lengthening, 3 posterior tibial tendon re-tensioning, 5 allux valgus correction using SERI technique, 1 case of second finger correction. No intra-operative or early complications were recorded. Late complications were recorded in 7 cases (5,8 %): 1 case of calcaneo-stop failure (due to fracture of the screw), 6 cases of intermittent walking pain 4 months after surgery.

**Discussion** Post-operative evaluations (clinical, radiographic and Gait Analysis) have shown the efficacy of both surgical procedure. No residual walking pain was observed at the final clinical follow-up. Post-operative X-rays have shown radiographic Meary line restoration compared with preoperative. Post-operative Gait Analysis has shown a progressive recovery of normal kinematic, kinetic and electromyographic results compared with pre-operative after 4–6 month, as expression of the surgical correction of flatfoot.

**Conclusions** Surgical correction of flatfoot can be completely achieved using both bioreabsorbable implants (calcaneostop and endorthesis). Moreover the big advantage of the use of bioreabsorbable implants is to avoid removal of the implants with a secondary surgery.

### *C19–FOOT AND ANKLE 4*

### Cylindrical cervical resection in biomechanical metatarsalgia

A. Valente*, I. Bagnoli, G. Peretti, L. Milano

Clinica Cellini (Turin, IT)

**Introduction** Metatarsalgia is one of the most common reports in patients with foot problems. This condition is often secondary to a first ray deficiency, which transfers loads to the lateral metatarsals. It can involve one or more lateral metatarsals and there is great variability in possible causative factors. The first-line management is non-operative: oral anti-inflammatory analgesics, physiotherapy and plantar orthotics. When these do not provide sufficient pain relief, a surgical treatment is indicated. Many types of osteotomy have been described: osteotomies at proximal, midshaft or distal metatarsal levels (i.e. Chevron, Weil, DMMO) with or without plantar plate repair or flexor tendon transfer. In the literature, there is no difference in outcomes between the operative treatments. Since 2006, in case of metatarsalgia, we use a cylindrical resection in the cervical region of the painful metatarsals. We reported surgical technique and our clinical outcomes.

**Materials and methods** Between 2006 and 2012 were treated 132 patients (156 feet). In 94 % of cases, metatarsal osteotomy was associated to a procedure on the first ray. Were performed weight-bearing radiographs of the forefoot with lateral, dorso-plantar and axial views in the pre operative. Cylindrical resection in the cervical region of the painful metatarsals was performed in order to restore correct metatarsal formula. We used internal fixation with K-wire for 5 weeks. Patients were assessed clinically and radiographically at 5 weeks, 4 months and 12 months after surgery.

**Results** In the 86 % of the patients there was pain resolution. In the 14 % of the cases there was residual metatarsalgia treated with plantar orthotics. Complications were: transitory forefoot oedema (23 %), complex regional pain syndrome (2 %), mal union and/or nonunion (7 %).

**Discussion** The management of metatarsalgia remains an open problem. There is no differences between our findings and those reported in the literature.

**Conclusions** This surgical technique allows us to predict metatarsal shortening particularly when we treat more rays. Furthermore, it is a simple technique, with good medium-term outcomes and low cost.

### Metatarsal lengthening by Ilizarov technique

S. Reverberi*

Casa di Cura Polispecialistica Villa Verde (Reggio Emilia, IT)

**Introduction** Metatarsal lengthening by distraction osteogenesis (or Ilizarov technique) is a very effective technique, especially if we need an important elongation. Despite being a proven technique, it is not devoid of complications.

**Materials and methods** We studied 25 patients operated by metatarsal lengthening with Ilizarov technique to evaluate number and severity of complications.

**Results** In our series complications observed were: subluxation of the MTP joint; stiffness of MTP joint; arthritis of MTF or IP joint; inclination of the fragments; broken screws (fiches); loss of anchorage of the chips from the bone; non-union.

**Discussion** Main observed complications are related to a major elongation (greater than 50 % of the original length of the metatarsal) or to a speed of elongation incongruous (generally too rapid for the possibility of soft tissues).

**Conclusions** Metatarsal lengthening by Ilizarov technique is a very effective method to correct deformities such as brachymetatarsia (congenital metatarsal shortening). If, however, we do not take into account the severity of the deformity of origin and do not observe the principles of good surgery we are likely to run into major complications. If we do everything to prevent them and are ready to deal with them, we will have a very high percentage of satisfaction of patients operated on with this technique.

### Metatarsal lengthening with LAKI external fixator

A. Kirienko*[1], A. Peccati[1], E. Arnaldi[1], N.M.A. Portinaro[2]

[1]Istituto Clinico Humanitas (Rozzano, IT);
[2]Clinica Ortopedica Università degli Studi di Milano (Milan, IT)

**Introduction** Shortening of one or more metatarsal bones can be congenital, associated with other deformities such as clubfoot, or post-traumatic. The aim of our work is to present our experience with lengthening of the metatarsal bones with the distraction osteogenesis technique in patients affected by congenital or post-traumatic shortening with the LAKI external fixator.

**Materials and methods** Between 2006 and 2011 we treated 25 metatarsal bones belonging to 16 patients (6 males and 10 females, meanly aged 19.22 years), affected by brachimetatarsia of the 4th ray (11 cases; 5 bilateral), congenital or post-traumatic brachimetatarsia of the 1st ray (3 cases, 2 bilateral), bilateral brachimetatarsia of the 3rd and 4th metatarsal bones (1 patient affected by Turner syndrome). Lengthening was performed with custom-made LAKI semicircular external fixator, fixing bone fragments with Kirschner wires, crossed-placed on the transverse plane; distraction was begun on 5th post-operative day with a 0.75 mm/day rate for 2 weeks, then lowered to 0.5 mm/day.

**Results** The mean time-in-frame was 16.32 weeks. The mean lengthening was 17.30 mm, with a mean 36.12 % increase of the initial length; the mean healing index was 61 days/cm. The most common complications have been rigidity of the metatarsal-phalangeal joint (6 cases) or its dislocation (2 cases), angular deviation of the regenerate (5 cases), delayed consolidation after lengthening (1 case, requiring a new surgery). No cases required bone grafting. The mean follow-up time has been 37.12 months.

**Discussion** Progressive distraction technique for metatarsal lengthening permits the accuracy required to allow the formation of a physiological arch of the metatarsal heads and changes in soft tissues, and at the same time the correction of associated deformities without requiring any kind of bone graft. Joint dislocation can be prevented by pinning the toes with longitudinally placed Kirschner wires.

**Conclusions** Excellent clinical and functional results have been gained with LAKI semicircular external fixator. This technique can be recommended as gold standard to treat metatarsal shortening. Bone lengthening for brachimetatarsia correction can be indicated not only for cosmetic purposes, but also to relief pain and plantar hyperkeratosis at the 2nd and 3rd metatarsal heads and to prevent secondary deformities of the other toes.

### Mechanical complications of first metatarso-phalangeal arthrodesis: biomechanical analysis and practice deductions

M. Stamilla*[1], F. Bonnel[2], P. Auteroche[2], M. Benzid[2]

[1]Catania, IT;
[2]Montpellier, FR

**Introduction** The pseudoarthrosis in case of arthrodesis of the first metatarso-phalangeal joint (MPJ) is a variable complication in international literature : 3 % using intramedullary screw fixation (Johanson), 5 % using K-wire (Mann); Coughlin gets 92 % of bone consolidation using plates. The purpose of this study was to compare and to evaluate the efficiency of 3 different plates.

**Materials and methods** We report retrospectively, homogeneous and continues a series of 126 patients. There were 102 women and 24 men. The mean age at surgery was 61 years (44–88). We operated 72 right feet and 54 left feet. Etiologies were the following: 85 hallux valgus, 20 revision of hallux valgus and 21 hallux rigidus. We always used dome-shaped reamers and 2 low-profile types with 10° dorsiflexion dorsal titanium plates with compression screw. The mean clinical and radiographic follow-up was 12 months.

**Results** Osteolysis was major, 5/30 cases, using standard plates; 2/96 cases using angular stable plates. There were 9 broken standard plates, 1 angular stable plate; only 2 cases were asymptomatic. We observed significant correlation with plate breaking and plate morphology.

**Discussion** Angular stable plates have not been a bone fusion assurance in our series. Buranosky (2001) used plates with compression screw; for Neufeld (2002) the importance of, first the screwing and after the plate. Coughlin (2006) obtained 92 % of bone fusion with cross screwing plate. Maestro (1997) preconized titanium plates. Bennett (2005), Goucher (2006), Shapiro (2009) proconized plates with intrafocal screw.

**Conclusions** Angular stable plates have not showed significant superiority of standard plates. The other anatomical factors must therefore be determined for the design of plates in the first metatarso-phalangeal joint (MPJ) arthrodesis.

### PRP in the treatment of Achilles tendinopathy

A. Sanfilippo*[1], G. Salomone[1], V. Colombo[2], D. Scaturro[2], A. D'Arienzo[1], M. D'Arienzo[1], G. Letizia Mauro[2]

[1]Clinica Ortopedica e Traumatologica, Università degli Studi (Palermo, IT);
[2]Cattedra di Medicina Fisica e Riabilitativa, Università degli Studi (Palermo, IT)

**Introduction** The Achilles tendinopathy is often a result of overuse and occurs in athletes; however, it is frequent in general population for inappropriate shoes, drugs, overweight, poor muscles. Clinically shows pain in the tendon area and the adjacent structure with functional limitation. An early diagnosis prevents the chonicization and its treatment is generally conservative. Surgery is advised when a conservative approach doesn't result after 6–8 months. In this study we refer to 24 patients with mild to moderate Achilles tendinitis treated with intratendinous infiltration of platelet-rich plasma (PRP).

**Materials and methods** The study was performed on 24 patients with mild or moderate Achilles tendinopathy (aged between 27 and 56 years). All reported localized pain in Achilles tendon about 2–4 cm from its insertion to heel and functional limitations, particularly during deambulation and going up and downstairs. Diagnosis were confirmed by ultrasonography. The patients were treated with a cycle of 3 PRP intratendinous infiltrations every 14 days. The platelet gel was prepared using autologous blood centrifuged to obtain a dialysate rich of platelets (1000000 platelets/$mm^3$), ready to inject. In the follow-up at 3 and 6 months the patients were monitored clinically and sonographically.

**Results** Clinical (pain, functional limitation) and ultrasonographic parameters showed a return to normality for the objective and subjective clinical parameters with normal ultrasound pictures in 16 patients. In 7 patients remained mild pain with slightly altered ROM and residual ultrasound thickening of the tendon. In 3 patients clinical and ultrasound data remained unchanged/little changed compared to initial.

**Discussion** PRP is a bioactive component with biological functions of regeneration and repair of soft tissue and a powerful anti-inflammatory effect.

**Conclusions** The treatment of Achilles tendinopathies with PRP affords good results to obtain the reduction of pain and correct deambulation recovery.

### Articular reconstruction with prosthesis in the treatment of hallux rigidus

R. Valentini*, L. Zandanel, M. Martino, G. Piovan, G. Fancellu

Clinica Ortopedica e Traumatologica, Università degli Studi di Trieste (Trieste, IT)

**Introduction** The degenerative joint disease of the hallux rigidus in the advanced stage has always been a challenge in surgical treatment. Over the years there have been several proposals of joint replacement surgical techniques with the aim of relieving pain, correcting deformity and maintaining a degree of motion.

**Materials and methods** In recent years, for the treatment of hallux rigid severe, we used a metal arthroplasty (Reflexion). We checked 25 patients (18 females, 7 males) of mean age 58.1 years, operated with this technique from June 2008 to June 2011. Truncal anaesthesia is performed on the ankle and is granted the immediate loading. The patients were followed up clinically and with radiographs (medium follow-up 18 months). The functionality was evaluated with the score of Kitaoka et al.

**Results** The return to normal activities was around 28 days (21–65). The mean score second Kitaoka was increased to 75 points at three months postoperative and 87.1 points after 6 months, starting from a preoperative score of 36.6. reached the average ROM was 42° (extension and flexion = 25.3° and 18.1°). We had a loosening of the implant with removal of prosthesis and the appearance of valgus of first MP with occasional pain in 4 cases.
**Discussion** Over the years various techniques have been proposed to alleviate pain, restore and maintain the motility of the first MF in patients with hallux rigidus. Our results appear to be in line with those obtained by other authors who use prosthetic replacement. That research should go towards improving the osteointegration of prosthetic stems to prevent the mobilization and the pain, perhaps creating anatomical stems with more fit. Only then prosthetic replacement of the first MP can be considered one of the best surgical solution.
**Conclusions** The results seem to be favourable, as even patient satisfaction is complete. We must improve the design prosthetic to get positive results to be comparable to those of knee and hip. We believe, however, that with this technique it maintains a certain degree of motion of the joint guaranteeing a good feature of the forefoot.

### Etiopathogenesis and treatment of biomechanical metatarsalgia: clinical and radiographic study

A. Folliero*[1], M. Minicelli[1], S. Ghera[2]

[1]Fisioeuropa (Rome, IT);
[2]Ospedale S. Pietro (Rome, IT)

**Introduction** The aim of this study is to investigate the role of foot morphology (cavus, flat and normal foot) and metatarsal formula in the etiopathogenesis of biomechanical central metatarsalgia. Also, we report the results of conservative treatment with the Valenti customized foot orthoses. When this treatment fails, we propose the subcapital metatarsal circular osteotomy stabilized with an absorbable pin for the treatment of this disease.
**Materials and methods** Between March 2006 and April 2012, 1815 feet with central metatarsalgia without hallux valgus (845 women and 211 men with a total of 1056 patients) were examined. All patients were aged between 40 and 85 years old. We evaluated the callosities by physical examination and the pain on the visual analogue scale (VAS). The weightbearing lateral and antero-posterior foot X-ray projection was used to evaluate the metatarsal index and formula. The baropodometric analysis was used to assess the type of plantar arch.
**Results** All symptomatic feet were cavus: 68 % of feet had three grade of cavus, 30 % two grade and 2 % had one grade of cavus or had a normal arch. The Morton's neuroma was found in 91 symptomatic feet. All cavus feet with central metatarsalgia had an index minus, the pain increased with age regardless of shoes. In patients aged over 40 years, we showed callosities beneath metatarsal heads as *durone plantaire* and in older there was the thinning of *plateau plantaire* with corns. All patients were treated with Valenti customized foot orthoses to correct the abnormal gait mechanics and to reduce the plantar pressure under the metatarsal heads. Eight hundred and ninety-seven patients were reviewed and evaluated with a mean follow-up of 5 years. The VAS showed improvement from 7 to 1 at follow up in 98 % of cases. Nineteen patients (30 metatarsals) were operated because no-responders to non-operative treatment.
**Discussion** In this study the metatarsalgia affects the cavus feet with 2 and 3 grade of cavus provided they have an Index minus. The metatarsal pain increases with age regardless of type of shoes.
**Conclusions** The central metatarsalgia may be predicted from an early age. The treatment with Valenti foot orthoses produces a remission of symptoms at long follow up and also the subcapital metatarsal circular osteotomy stabilized with an absorbable pin for the no-responders reports excellent results. This operative technique allows to distribute the load uniformly on the plantar metatarsal heads.

### *C20–SHOULDER AND ELBOW 5*

### Surgical treatment of upper limb spasticity

G. Caserta*[1], G. Petrella[1], M.C. Gagliano[1], A. Barbati[2], A. Landi[1]

[1]Chirurgia della Mano (Modena, IT);
[2]Policlinico Universitario "S.S. Annunziata" (Chieti, IT)

**Introduction** The authors provide their 20 year experience in surgical treatment of upper limb spasticity. They show the pre-surgical evaluation, with the spinal centre specific for each region, which gives directions for the upper limb surgical treatment.
**Materials and methods** The authors report their experience of 242 treated patients on a total of more than 500 surgical treatments performed in almost 22 years of activity. They also provide long term results and the pre and post-surgical evaluation procedures the patients go through.
**Results** This treatment can be aimed to a hygienical and postural goal or to a functional recovery.
**Discussion** This treatment can be aimed to a hygienical and postural goal or to a functional recovery. In the first case, the surgical treatment consists of tenotomies to free the upper limb from forced and few hygienical behaviours or arthrolysis of the upper limb joints. In the case of functional surgery, after a careful analysis and treatment tests with botulinum toxin, it consists of a fractioned extension of the muscles considered as spastic at the myotendinous junction. In some cases, the surgery can reactivate a lost function by means of a muscles transfer.
**Conclusions** However, in this case the surgeon must ensure that the muscle aimed to the transfer is not spastic through a careful electromyographic analysis. For this reason, palliative surgery in spasticity is less frequent than in tetraplegia.

### Arthroplasty of capitulum humeri: experience with anatomic prosthesis

M. Zanlungo*, E. Finardi, F. Nasi, F. Caracciolo

Isituto di Cura Città di Pavia (Pavia, IT)

**Introduction** Malunion's capitulum humeri causes severe stiffness of the elbow in flexion and extension. Spontaneous signs of arthritis begin on joint's radial side and led to disability. At present two types of prosthesis are available: one type with anatomic reconstruction of condyle (SBI Mirmex and Tornier) and the second type at joint surface partial reconstruction (BIOMET).
**Materials and methods** Between 2009 and 2012, 7 patients were treated with arthroplasty of humeral capitulum: Patients were: 6 women and one man, 41 to 76 years old; 5 cases of malunion of humeral condyle fractures, 1 woman with Essex-Lopresti fracture outcome, 1 labourer with evidence of arthritis on joint radial side. All patients complained of an important limitation of flexo-extension of the elbow that limit hand function at work and at daily activity. We implanted 6 press-fit humeral component SBI Mirmex and one Tornier with press-fit on humeral component and cemented for radial capitulum reconstruction. The fisiokinesy-terapy treatment started in second day after redon took and supplemented by Quenquel dynamic arm brace.
**Results** According to the Morrey Score results were 1 very good, 5 good and 1 fair: 5 patients with post-traumatic malunion recovered elbow without painful with extension range −30°, flexion 120°; in patient with Essex-Lopresti prono-supination was limited of 1/3, the labourer with elbow arthritis resumed his occupation with pain reduction, flexion 135°, extension −30° and complete prono-supination. There wasn't any complication.

**Discussion** These starting experience are positive for very good primary stability of the implants and for early osteointegration. As in the case of radial capitulum have not to overdimensioned the implant: it's necessary X-ray study during the operation and pre-operatory X-ray study of healthy side. In any case the flexion recovery it's better than extension recovery. A larger number of sizes available as we have in greater prosthetic surgery cancan be able to reconstruct anatomically humeral condyle and improve the results.

**Conclusions** The arthroplasty of radial side of elbow in the in traumatic outcomes and arthritis grade 1–2 is now surgical reliable possibility also with only arthroplasty of humeral condyle, without macroscopic lesions of radial capitulum.

### Negative prognostic factors in complex elbow instability: a prospective study on 76 patients

G. Giannicola*, D. Polimanti, G. Bullitta, M. Scacchi

Università "La Sapienza" di Roma (Rome, IT)

**Introduction** A significant improvement in the results of surgical treatment of complex elbow instability (CEI) has been observed in recent years; nevertheless, a consistent percentage of complications and reoperations persist in CEI. Our aim was to identify, in a large group of patients with CEI, the main negative prognostic factors that affect outcomes.

**Materials and methods** Seventy-six patients (78 elbows), 35 males and 41 females, mean aged 52 years, were prospectively followed-up for an average of 70 months. The elbow injuries consisted of 5 radial head fracture-dislocations, 5 coronoid fracture-dislocations, 22 terrible triads, 35 fracture-dislocations of the proximal radius and ulna, and 11 capitulum humeri and trochlea fractures with a posterior dislocation. A standard X-ray and CT scan were performed before surgery in all the patients. Surgical treatment consisted of ORIF of all fractures and radial head replacement in comminuted fractures. Following ORIF, soft tissue constraint lesions were repaired and, if instability persisted, a hinged elbow fixator was applied. All the patients started an early rehabilitation program. The clinical outcome was evaluated by means of the MEPS, ASES and DASH score.

**Results** Unsatisfactory results following the first operation were associated with poor general conditions in 60 % of the patients, lack of co-operation in 70 %, obesity in 70 %, late surgery in 50 % and high-energy trauma in 30 %. Thirty-one of the 75 patients developed heterotopic ossification, which was asymptomatic in 91 % of the cases. Thirty-two patients displayed signs of arthrosis, which was severe in 2 cases. Four patients developed severe stiffness. Thirteen percent of the patients underwent a second operation. At the last follow-up, the average MEPS, ASES and DASH were 90.2, 87.4 and 11.9 points, respectively, with excellent results at the MEPI in 64 % of the cases, good in 28 %, fair in 6.5 % and poor in 1.5 %. There were no significant differences between the clinical results in the different CEI patterns.

**Discussion** Stiffness and instability were the underlying reasons for the unsatisfactory results. Proximal radio-ulnar synostosis, arthrosis and extrinsic contracture were the main causes of stiffness. Severe osteoarthrosis and chronic instability were often associated in the same patients; these patients were frequently uncooperative and/or obese. The intensity of the trauma, delayed treatment and poor general conditions were other negative prognostic factors.

**Conclusions** In CEI, the psycho-physical characteristics of the patient, the intensity of the trauma and the timing of the intervention may affect the final clinical outcome, regardless of the fracture pattern or of the surgery performed.

### Glenoid defects reconstruction in posterior instability of the shoulder using autologous tricortical iliac crest graft

A. Busilacchi, D. Falcioni*, D. Enea, S. Cecconi, A. Gigante

Clinica Ortopedica, Università Politecnica delle Marche (Ancona, IT)

**Introduction** Posterior instability of the shoulder is a relatively rare occurrence, accounting for 4 % of all shoulder instability. Several factors may simultaneously contribute to posterior instability: the traumatic one in glenoid hypoplasia or dysplasia is relatively frequent. When conservative treatment fails, the condition can be surgically addressed with several techniques. The purpose of this study was to report the outcomes of patients with posterior shoulder instability treated with an autologous tricortical iliac crest graft and a tendon/muscle-sparing surgical procedure.

**Materials and methods** Between 2006 and 2012, 3 patients (2 females, 1 male) have been treated with the same surgical technique. Any of the three patients had a history of traumatic recurrent posterior dislocation and a diagnosis of glenoid hypoplasia or dysplasia documented by preoperative imaging. Surgical procedure: patient in the lateral decubitus; arthroscopic examination to assess any associated injury; posterior approach with a vertical incision without sectioning the posterior bundle of deltoid muscle; intermuscular dissection between infraspinatus and teres-minor to expose the posterior part of the capsule sparing the muscle–tendon tissues; arthrotomy by a vertical incision situated on the posterior edge of the glenoid labrum; abrasion of the posterior edge of the glenoid labrum and neck to insert an autologous tricortical iliac crest graft fixed by two cannulated screws; one patient also underwent a treatment for an associated SLAP lesion. After the surgical procedure a brace was worn for 40 days post-op to maintain the arm in abduction/external-rotation, following a rehabilitation of 4–6 months.

**Results** The average follow-up was 40 months. All patients completely recovered active ROM, with mild pain in the last 20° of abduction and in the final phase of intrarotation/adduction (Apley-Test). During the follow-up no patient showed signs of glenohumeral osteoarthritis nor recurrence of posterior dislocation.

**Discussion** According to the review of the limited case series in the literature, the clinical and functional outcomes of our study are similar to those of other authors, as regards the degree of functional recovery and patients' satisfaction, as well as by the absence of recidivism. However, it is a technique requiring accuracy in particular during the positioning, modelling and synthesis of the bone graft. We also believe that such tissue-sparing approach represents an improvement from a biological and functional point of view compared to the classical approach, especially in the short-term results.

**Conclusions** The posterior tricrotic iliac crest graft therefore can be considered an effective technique in the treatment of traumatic posterior instability caused by glenoid hypoplasia.

### *C21–SPINE 1*

### Brace treatment in juvenile idiopathic scoliosis: a prospective study with outcomes in agreement with SRS committee on bracing and non-operative management standardization criteria

A.G. Aulisa*[1], M. Giordano[1], A. Poggiaroni, M. Peruzzi[2], V. Guzzanti[3]

[1]Ospedale Pediatrico Bambino Gesù (Rome, IT);
[2]Policlinico Gemelli, Università Cattolica del Sacro Cuore (Rome, IT);
[3]Università degli Studi di Cassino (Cassino, IT)

**Introduction** The Juvenile idiopathic scoliosis by age of onset, severity and evolution is source of great doubts concerning the purpose and use of conservative treatment. The different clinical experiences leave unsolved the question that arises in applying a conservative treatment when the patients are effectively forward a long growing period, in scoliosis characterized by inevitable evolution. The aim of this prospective study was to determining the effectiveness of conservative treatment.

**Materials and methods** From 1238 treated for idiopathic scoliosis between 1995 and 2012 fulfil the inclusion criteria (age between 4 and 10 years, full-time prescription) 163 patients treated with PASB, Lyon brace and Milwaukee. Of these, 113 patients had a definite outcome, 27 have abandoned treatment e 23 are still in treatment. The minimum duration of follow-up was 24 months. Antero-posterior radiographs were used to estimate the curve magnitude (CM) and the torsion of the apical vertebra (TA) at 5 time points: beginning of treatment (t1), 2-year minimum follow-up(t5). Three outcomes were distinguished in agreement with SRS criteria: curve correction, curve stabilization and curve progression. Moreover results were evaluated according to compliance dividing patients into 5 subgroups. Statistical analyses were performed with GraphPad Prism 6.

**Results** The results from our study showed that of the 113 patients with a definite outcome CM mean value was 30.55 ± 5.16 SD at t1 and 21.9 ± 7.65 SD at t5. TA was 13.58 ± 6.14 SD at t1 and 8.95 ± 5.82 at t5. The variations between measures of Cobb and Perdriolle degrees between CM t5-t1 and TA t5-t1 were statistical significantly different. Curve correction was accomplished in 88 patients (77.8 %), whereas a curve stabilization was obtained in 18 patients (15.9 %). Seven patients (6.19 %) have a curve progression and of these, 4 (3.5 %) where recommended for surgery. Of 26 patients who abandoned the treatment, at the time of abandonment (12.4 age) have achieved curve correction in 19 cases (73.0 %), curve stabilization in 5 cases (19.2 %) and curve progression in 3 cases (11.5 %). Of these patients, reviewed at the end of growing, four have been operated on. In addition, there is a statistical significant correlation between compliance and result from T1–T5 with an interaction of 3.43 $p < 0.0001$.

**Discussion** Our study confirmed that most patients reaching a complete curve correction and only 4.9 % of patients need surgery. It also confirms how to obtain good results, the full-time and patient compliance is essential.

**Conclusions** The conservative treatment with brace is highly effective in treating juvenile idiopathic scoliosis.

### Surgical treatment of congenital kyphosis and kyphotic scoliosis

M. Crostelli*, O. Mazza, M. Mariani, D. Mascello

U.O. Patologia del Rachide, Ospedale Pediatrico Bambino Gesù (Rome, IT)

**Introduction** Congenital vertebral deformities are caused by formation or segmentation defects of vertebrae. Kyphosis and kyphotic scoliosis generally require surgical treatment due to high risk of curve progression and of potential neurological complications. Modern correction techniques, including hemivertebrectomy, allow radical correction of deformity without the need of adjunctive anterior approach.

**Materials and methods** From 2006 to 2012 we operated 12 patients (10 males, 2 females) with kyphosis or kyphotic scoliosis by posterior approach by different techniques, chosen depending on malformation (subtraction osteotomy, hemivertebrectomy), and by instrumented arthrodesis with pedicle screws. Mean age at the time of surgery was 7 years (range 5–15 years) and 8 patients was under 10 years of age. Mean kyphotic curve was 75° Cobb, mean scoliosis curve was 44° Cobb. Mean follow up was 3 years (range 1–7 years). All patients have been operated by the same surgeon. In interventions until 2011 we did not use intra-operative neurophysiologic monitoring because it was not available in our hospital, this monitoring has been used in interventions after 2011.

**Results** Mean kyphotic curve after surgery has been reduced to 20° Cobb, mean scoliosis curve to 11° Cobb. We had no major complication at follow-up (neurologic, vascular or visceral lesions, instrumentation failures with loss of correction, infections).

**Discussion** Different posterior approach procedures by pedicle screws instrumentation are less invasive than combined anterior-posterior approach procedures and are well tolerated even by very young patients. Pedicle screws have great grip in vertebral bodies and can obtain important corrections that in different cases can be maintained by filling of the empty space created by correction with bone obtained by osteotomy/hemivertebrectomy, alone or with use of cages; if patient is treated early, before secondary curve structuring, arthrodesis segment could be relatively short. Surgical strategy must be accurately studied and complete imaging study is paramount.

**Conclusions** We think that posterior only approaches allow excellent deformities correction on sagittal plane and, when it is necessary, on frontal plane, optimal stability, low risk of neurological lesions, no need for adjunctive anterior approach procedures; intervention should be proposed as early as possible to avoid deformity progression.

### The Michel-Allègre three points brace in the treatment of idiopathic scoliosis: long follow-up review

A. Martucci*, S. De Giorgi, S. Tafuri, A. Piazzola, B. Moretti

Azienda Ospedaliero-Universitaria Policlinico di Bari (Bari, IT)

**Introduction** The spine is a viscoelastic structure which becomes tight in case of scoliosis. The corsets represent an effective device in the treatment and restraint of the curves. The Michel-Allègre brace's aim is to correct the closure of ilio-lumbar angle in lumbar and thoraco-lumbar idiopathic scoliosis with limit vertebra not exceeding T9 in reducible curves, without overlying structured curve, in order to straighten the spine through a translation of the trunk on the pelvis. The brace, made of plexidur and aluminium, has three valves adjustable on the coronal plane: the correcting ilio-lumbar valve and the compensating thoracic and pelvic ones.

**Materials and methods** We carried out a long follow-up study (mean f.u. 8.8 ± 2.7 years) to evaluate, through the examination of specific clinical and radiological parameters, the real efficiency of the treatment, by analyzing the persistence of a good correction and the related changes. The study was a retrospective cohort-type model of a group made up of 60 patients treated between 1994 and 2006, whose data were analyzed using Stata MP11 software.

**Results** Our analysis has showed that the three points brace has been effective in the restraint of the curve with an improvement of 8 % at the end of the treatment, which stands at 4 % at follow-up. The ilio-lumbar angle and the C7 plumb line have improved respectively by 17 % and 27 %, despite the rotation that worsen by 17 %; sagittal parameters haven't shown significant variations. Clinically we noticed a slight improvement in the extent of bulges. Comparing lumbar to thoraco-lumbar scoliosis we observed a significant improvement of C7 plumb line in the thoraco-lumbar one. The psychological test SRS-22 showed a satisfaction rate of 77.4 %.

**Discussion** The three points brace seems to be effective in preventing the worsening of scoliosis with ilio-lumbar angle closure, improving the verticality and the appearance of the patient, but unable to control the worsening of the vertebral rotation. It allows a satisfactory restraint of curve maintained even after many years and a good quality of life.
**Conclusions** The three points brace could be still considered a valid mean for the treatment of lumbar and thoraco-lumbar scoliosis, as long as the prescription follows a good evaluation of the characteristics of the curve. The good acceptance of the brace allows a discrete treatment compliance, without affecting the normal psychological development of patients.

### *C22–SPINE 2*

### Heart rupture associated to vertebral fracture: two cases report

S. Paderni*, M. Cappuccio, G. Bosco, L. Amendola, A. Corghi, F. De Iure

Chirurgia Vertebrale d'Urgenza e del Trauma, Ospedale Maggiore (Bologna, IT)

**Introduction** Literature does not report cases of vertebral fractures associated with heart rupture. In the revision of autopsy case studies, we can find descriptions of lesions of the thoracic aorta associated with thoracic vertebrae fractures and the only one case of vertebral fracture associated with tracheo-bronchial rupture.
**Materials and methods** The authors report 2 cases of multiply injured patients, victims of car crashes, resulting in the rupture of the right atrium together with fractures (or fracture-dislocation) of the high thoracic spine. Both cases (male, 23 years, motorbiker and female, 36 years, car driver) underwent emergency thoracotomy and repairing of the right auricle tear. Once the general conditions of the patients were stable other vertebral fractures were diagnosed: fractures of T3–T4–T5–T6 with dislocation T4–T5, anterior dislocation of the body of the sternum, dislocation of the left costo-transverse articulations from T7 to T10 in the first case; fracture of the base of C2, fracture of T2 with a mild reduction of the spinal canal in the latter. In the first case, eight days after trauma, decompression and posterior fixation C7–T9 were performed. In the second case posterior fixation C5–T4 was performed 10 days after the trauma. Stabilizing the T2 fracture allowed surgeons to position the patient in a lateral decubitus so that an expandable metal endoprosthesis could be inserted in the left bronchus.
**Results** Both patients underwent several hospital admissions due to pulmonary problems and received rehabilitation at a specialized structure. Fifteen months from surgery, the first patient who woke up after surgery tetraplegic with level C5–C6 recovered the use of his upper limbs, with residual paraplegia level T5, ASIA score A. Nine months from surgery, the second patient presented no peripheral neurological deficit.
**Discussion** The approach to these patients has to be multidisciplinary: management and treatment of vertebral fractures should be given once the general conditions of the patients are stable, but sometimes they are mandatory to allow further subsequent treatments. Circulatory shock related to a cardiac lesion may worsen an actual neurological damage.
**Conclusions** Nowadays, pre-hospital trauma care improvement and early application of resuscitation manoeuvres allowed an increase in the survival rate of these patients. Prognosis is strictly dependant on the precocity of diagnosis and treatment and to the severity of associated lesions.

### Post-traumatic upper cervical instability treated by C1–C2 and C1–C3 posterior fixation

L. Amendola*, M. Cappuccio, G. Bosco, A. Corghi, L. Mirabile, S. Paderni, G. Scimeca, F. De Iure

UOSD Chirurgia Vertebrale del Trauma e dell'Urgenza, Ospedale Maggiore (Bologna, IT)

**Introduction** To achieve a posterior stable fixation of the post-traumatic upper cervical instability, the occiput is often included. With the new occiput sparing techniques, cervical motion impairment is reduced, while still allowing a stable fixation.
**Materials and methods** The authors report their experience on 21 post-traumatic upper cervical instability treated in 6 years: 13 cases with the classic Harms' technique and 8 cases with C1–C3 posterior fusion. There were 13 men and 8 women, with an average age of 50 years.
**Results** Harms' technique has been used in 5 non-unions of the odontoid (after Halo-vest, collar or anterior screw failure), 4 acute fractures (three patients were polytrauma, and one patient refused the Halo-vest), three C1–C2 post-traumatic instability and one os odontoideum C1–C3 posterior fixation has been used in 7 case of complex fractures of C2 vertebral body, and one case of odontoid non-unions.
**Discussion** Post-operative CT scans showed proper placement of all the screws, except for one in C1 that was too medial, without mechanical or clinical relevance. Posterior fusions were achieved on an average of 4 months. At 12-months follow-up, 4 patients reported intermittent neck pain and head rotation impairment was 40° (20 on each side) compared to normal ROM. Flexion and extension ROM was close to normal.
**Conclusions** In our experience, these techniques showed to be effective and reliable in treating post-traumatic upper cervical instability although requiring a long learning curve, careful preoperative imaging study and a detailed knowledge of regional anatomy.

### *C23–SPINE 3*

### Results and risks of surgery to correct spinal deformities in adult and elderly

G.A. La Maida*, L. Zottarelli, G.V. Mineo, B. Misaggi

Istituto Ortopedico Gaetano Pini (Milan, IT)

**Introduction** The adult scoliosis is primarily degenerative with a prevalence of 1 % to 10 % and it affects more than 30 % of adults without a history of spinal deformity. The surgical treatment is no longer a technical problem since we have last generation of vertebral instrumentation, with the possibility of using cemented screws combined with vertebral osteotomies. The osteotomies we regularly used are the Ponte procedure (PP) for the thoracic spine, the Smith-Petersen osteotomy (SPO) in the lumbar region and the pedicle subtraction osteotomy (PSO). This last procedure is particularly complex and subject to a higher risk of general and neurological complications.
**Materials and methods** Our experience is based on 110 cases (ages between 30 and 72 years) of spinal deformity operated since 2007. All patients underwent extended posterior surgery (thoraco-lumbar) for the treatment of the spinal deformity (treatment levels V and VI of Lenke-Silva).
**Results** Eleven patients (11/110) had a complication (10 % of cases) with a reoperation rate of 5.4 % (6 patients undergoing reoperation). Patients over sixty years (30/110) were analyzed clinically and radiographically in order to evaluate the results but also to assess the complication rate of this type of surgery. In the group over 60 years (n = 30) we observed a complication rate of 26.6 % (8/30) for a total of 13 complications in 30 patients (43.3 %). The complications we observed were: 1 deep infection, 2 root injuries (1 transient), 3 dural tears, 1 pulmonary embolism, 6 hardware failures. The rate of re-intervention was 16.6 %. All patients had at least one co-morbidity and three patients who had a complication (37.5 %) were over 65 years old and underwent a PSO.

**Discussion** The complication rates reported in the literature about adult deformity surgery vary according to the type of surgical procedure performed and in particular we can have a risk of 2.5 % to 35.2 % for the procedures of spinal decompression alone, which increases to 21.4 %–37 % in the case of instrumented spinal fusion and becomes dramatically high with a mortality rate of approximately 10 % in patients older than eighty years.

**Conclusions** In conclusion, patients with more than 60 years with severe thoraco-lumbar kyphoscoliosis candidates for posterior surgery (treatment levels V and VI of Lenke-Silva) have a high risk of complications, estimated in the order of about 40 %. In particular, the risk of complication seems to be particularly high in patients older than 65 years and in those undergoing PSO. Surprisingly the degree of patient satisfaction is very high, even in those who have experienced a complication.

### Results and complications in thoraco-lumbar spine surgery in patients aged over 70 years

G. Gargiulo*, F. De Salvo, N. Ruggieri

U.O. Ortopedia, Città della Salute e della Scienza (Turin, IT)

**Introduction** An increasing number of people aged over 70 years, biologically active, may require surgical treatment for complex diseases of the thoraco-lumbar spine, exposing to increased risk of peri-operative complications. Authors report their experience in these population affected by morbidity and mortality.

**Materials and methods** During the period 2005–2010 were surgically treated 41 patients of mean age 74 years (70–81) suffering from lumbar spinal stenosis (30), results of fracture in osteoporosis (5), secondary tumours (3), previous surgical failures (3). All complained of low back pain (VAS 8), claudicatio in 33, paraparesis in 6 (ASIA C 4, ASIA B 2), 16 patients were overweight, 3 had signs of Parkinson's disease, 21 cardiac disorders (FA in 2, hypertension in 19), 3 severe pulmonary deficit, 2 DVT which required a vena cava filter pre-operatively, 19 diabetes, 2 chronic liver diseases, 3 rheumatoid arthritis. Twenty-three were subjected to decompression 2 or more levels, 17 decompression and instrumented arthrodesis 2 or multiple levels, combined anterior-posterior decompression and instrumented fusion in a case. Average blood loss was 300 cc (100–1050) and surgical times 3 h (2–5). Among minor peri-operative complications we report two urinary infections, 5 delay wound healing, 3 delirium exceeding 24 h, among the bigger 4 re-operation of cases the surgical wound, post-operative DVT in 2 cases, and one cecum ischemia in a plurioperated Parkinson obese patient suffering from chronic constipation. A patient with wound dehiscence in metastasis died of progressive deterioration of the general conditions in 21 days. Altogether we observed 18 complications in 12 patients (30 %), more than one in six.

**Results** At a follow-up of 3 years (1–5) the clinical result was satisfactory in most cases (VAS 4) but with a result less than their expectations in 14 (34 %), with improvement of radicolalgia and claudicatio in almost all cases and better recovery of paraplegia (ASIA C 1, ASIA D 3, ASIA E 1). Five patients complained of pain from the iliac crest, a failed back patient complained the persistence of sciatica, 11 recur from time to time to physiatrist care, 4 patients required a new foraminotomy at an average distance of 1 year.

**Discussion** Despite the variety of different treatment options, surgery of the spine of the elderly differs from that of adults in the therapeutic choice and clinical results because it must take into account the patient's general status, co-morbidities and functional expectation.

**Conclusions** The aim of the treatment is a result clinically and radiographically minor comparing to adults but functionally acceptable after a careful evaluation of the overall medical needs to be made aware that the same patient exposing advantages and disadvantages compared to.

### A prospective study in adolescent idiopathic scoliosis affected by thoraco-lumbar and lumbar curves in treatment with a progressive action short brace (PASB): assessment of results according to the SRS criteria

A.G. Aulisa*[1], F. Falciglia[1], M. Giordano[1], M. Peruzzi[2], V. Guzzanti[3]

[1]Ospedale Pediatrico Bambino Gesù (Rome, IT);
[2]Policlinico Gemelli, Università Cattolica del Sacro Cuore (Rome, IT);
[3]Università degli Studi di Cassino (Cassino, IT)

**Introduction** The effectiveness of conservative treatment of scoliosis is controversial. In recent retrospective studies we have demonstrated the effectiveness of progressive action short brace (PASB) in correcting and stopping thoraco-lumbar and lumbar curves. Furthermore in recent years have been published several papers on adolescent idiopathic scoliosis in agreement with SRS criteria but none of them was prospective. The purpose of the present study was to confirm the effectiveness of PASB in the correction of thoraco-lumbar and lumbar curves, in agreement with the Scoliosis Research Society (SRS) Criteria in a prospective study.

**Materials and methods** We have carried out a prospective study of 197 adolescents (mean age 11.8 ± 0.5 years) treated from 1993 to 2012 with thoraco-lumbar and lumbar curve and a pre-treatment Risser score ranging from 0 to 2. To evaluate the effectiveness of treatment and standardize the results were used the SRS criteria. All patients were prescribed with a full-time PASB. The minimum duration of follow-up was 24 months. Antero-posterior radiographs were used to estimate the curve magnitude (CM) and the torsion of the apical vertebra (TA) at 5 time points: beginning of treatment (t1), and 2-year minimum follow-up (t5). Three outcomes were distinguished: curve correction, curve stabilization and curve progression. Statistical analyses were performed with GraphPad Prism 6.

**Results** Patients with least 2 years of follow-up were 136 (69 %), 37 were still in treatment (19 %), while patients who abandoned the treatment were 24 (12 %). Of these 24 patients who abandoned the treatment, at the time of abandonment, have achieved curve correction in 20 cases, curve stabilization in 3 cases and curve progression in 1 case. Of these 9 were revised to follow-up and one patient where recommended for surgery. In patients with a definite outcome CM mean value was 29.06 ± 4,73 SD at t1 and 13.58 ± 8.7 SD at t5. TA was 11.56 ± 5.53 at t1 and 7.4 ± 4.93 at t5. The variations between measures of Cobb and Perdriolle degrees between CM t5-t1 and TA t5–t1 were statistical significantly different. Curve correction was accomplished in 120 patients (88 %), whereas curve stabilization was obtained in 13 patients (10 %). Three patients (2 %) have a curve progression and nobody where recommended for surgery.

**Discussion** Our study confirmed, also in a prospective study, that the PASB is highly effective. Moreover the PASB has an excellent compliance with a dropout of only 12 %.

**Conclusions** The PASB is able to correct the thoraco-lumbar and lumbar curves with most patients reaching a curve correction and especially in reducing surgery.

### Minimally invasive SI joint fusion. Prospective, minimum 6-month follow-up

F. Valli*[1], M. Teli[1], C. Angelini[2], M. Sofia[2], D. Prestamburgo[1]

[1]UO Ortopedia e Traumatologia, Ospedale Civile Legnano (Legnano, IT); [2]Terapia del Dolore e Cure Palliative, Ospedale "G. Salvini" (Garbagnate Milanese, IT)

**Introduction** Aim of the present study is to analyze the early clinical results of percutaneous fusion of the SI joint with a titanium device, without bone graft implementation.
**Materials and methods** In a prospective longitudinal study, the authors evaluated 7 consecutive adult patients affected by degenerative SI joint pain without neurologic symptoms or evidence of systemic disease. Patients were treated with unilateral (7 cases) or bilateral (1 case) fusion of the SI joint with three titanium implants per joint. The entire surgeries were performed through a 3 cm stab incision in the upper buttock and AP, lateral, inlet and outlet fluoroscopy views. No bone graft or bone substitutes were applied. Inclusion criteria were repeated SI joint suppression tests, full medical workout to exclude systemic or inflammatory disease, and no evidence of symptomatic spinal pathology as reviewed by two consultant spinal surgeons. Clinical outcome was assessed using a VAS scale and the ODI score.
**Results** At 8.2 months follow-up, 75 % of the patients were satisfied with their outcome; 25 % of patients did not perceive any positive effect from surgery at follow-up. A single patient showed arterial bleeding during surgery that was difficult to control and required 2 units of blood transfused after surgery. She did not have any benefit from surgery at 6 months follow-up and requires continuous medical pain treatment. A second patient with postpartum SI chronic pain failed to show any improvement at follow-up. Overall, improvements in the VAS and ODI scores were clinically and statistically significant—from 8.1 to 2.1 and from 56.8 to 32.8 %, respectively. The radiographic fusion rate was not interpretable at this follow-up. No mobilisation of the implants occurred up to this stage. The cost of implants was higher than the average one level lumbar posterior fusion with pedicle screws and cages.
**Discussion** Minimally invasive fusion of the SI joint can be an effective tool in ¾ patients after careful medical and surgical workout of their source of pain. Complications at short-term follow-up are minor. Hospitalisation was shorter compared to low back fusion cases but the cost of implants is higher at the present stage.
**Conclusions** The present study suggests that percutaneous SI fusion can be effective at short term when degenerative chronic SI joint pain is carefully diagnosed with a full medical and suppression test workout at the expense of higher implant costs and lower surgical complication rates compared to low back fusion.

### *C24–BASIC SCIENCES*

### Muscle atrophy and fragility fractures: microstructural study of muscle tissue by MR-DTI

M. Celi*[1], J. Baldi[1], G. Di Pietro[2], E. Piccirilli[1], E. Gasbarra[1], S. Capuani[2], U. Tarantino[1]

[1]Università degli Studi di Roma "Tor Vergata" (Rome, IT); [2]Università degli Studi di Roma "La Sapienza" (Rome, IT)

**Introduction** The microstructural alterations involved in osteoporotic bone are studied by time through imaging techniques adopted both in vitro and in vivo; the muscle on the contrary, due to its plasticity, it is difficult to analyze. Aim of this study was to investigate the microstructural features in muscles of osteoporotic and osteoarthritic women by using magnetic resonance (MR) diffusion tensor imaging (DTI). Toward this goal we examined in vitro at 9.4 T the vastus lateralis biopsy of osteoporotic and osteoarthritic subjects (taken during a primary total hip arthroplasty procedure) by measuring mean diffusivity (MD), fractional anisotropy (FA), the three eigenvalues ($\lambda1$, $\lambda2$, $\lambda3$) of muscles and assessing associations between DTI parameters, subjects age, subjects bone mineral density (BMD) and subjects body mass index (BMI).
**Materials and methods** For this study we performed vastus lateralis biopsy in 10 women with osteoporosis (mean age $= 82.3 \pm 4.5$) underwent primary total hip arthroplasty (THA) for hip fracture, and 10 age-matched women (mean age $= 75.0 \pm 5.5$) underwent surgery for hip osteoarthritis. This study was approved by the local ethics committee and written informed consent was obtained before study initiation. A 9.4 T MR system with a micro-imaging probe was used to investigate muscle samples. Each muscle of 2 cm in length was stored in a 4 %-paraformaldeyde and PBS. The DTI protocol with b-values = 400 and 700 s/mm$^2$ was applied, using a PGSTE-imaging sequences (TE/TR = 14.5/2500 ms, diffusion delay = 40 ms, diffusion gradient pulse duration $\delta = 2$ ms, field of view = 0.75 cm, number of average NS = 4, slice thickness ST = 1 mm, twelve axial slices). Mean values and standard deviation were obtained for each variable for osteoporotic and osteoartritic subjects. Between-group comparisons to assess group differences and Pearson correlation analysis were performed. $p$ values $< 0.05$ were considered statistically significant.
**Results** FA was significantly higher in osteoartritic compared to osteoporotic subjects while MD, $\lambda2$ and $\lambda3$ were lower in osteoartritic compared to osteoporotic subjects ($p = 0.039$, $p = 0.040$ and $p = 0.022$, respectively). No significant difference in $\lambda1$ was found between osteoporotic and osteoartritic. A significant linear correlation was found between FA and BMI in osteoporotic subjects only. No significant correlation was found between DTI parameters, BMD and age.
**Discussion** Our in vitro preliminary results highlight differences in DTI parameters between OP and OA muscles, confirming previous histological evidences.
**Conclusions** Although it is necessary to correlate these results to histological data, it is interesting to study the possibility, through methods of in vivo imaging, the model micro-architectural muscle in diseases related to muscular skeletal disorders.

### Use of incisional negative pressure wound therapy in orthopaedics and traumatology

D. Luciani*, P. Capra, E. Chiarello, M. Cadossi, G. Tedesco, S. Giannini

Clinica I Ortopedia e Traumatologia, Istituto Ortopedico Rizzoli (Bologna, IT)

**Introduction** In the last few years, negative pressure therapy applied to post-surgical closed incisional wounds (incisional negative pressure wound therapy, INPWT) has grown in popularity in orthopaedics and traumatology. Surgical debridement is still the first step in the treatment of complex or contaminated wounds. The INPWT uses particular dressings and a constant suction of −80 mmHg; the goal is to achieve a reduction of the exudates, a reduction of perilesional tissue pressure, promoting wound healing and reducing the risk of surgical site infection.

**Materials and methods** From October 2010 to-days we have treated 11 patients In our institution (9 males and 2 females) aged between 15 and 71 years (average 49 years): 5 patients with a diagnosis of vascular insufficiency, 3 patients with open fracture and 3 patients for aseptic loosening of hip prostheses. Since the first postoperative day all patients received INPWT set at −80 mmHg until wound healing (closed and dry post-operative wound). Patients fill out a numeric rating scale (NRS) for pain and Asepsis Score (AS) for evaluation of wound healing every time the dressing was changed.
**Results** Five patients underwent surgical debridement, 3 patients required transmetatarsal amputation and 3 patients received a revision of hip prosthesis. The INPWT has been used for 29 days (range 6 to 44 days). Patients reported a pain of 15 mm to NRS score during the dressing change. The final AS was satisfactory for all patients and all reported a closed and dry wound.
**Discussion** INPWT has showed to be effective in the treatment of difficult postoperative wounds or at risk of infection. Our results in agreement with the literature; all patients reported a satisfactory AS. In no one case there was a deep infection, suffering skin or wound dehiscence.
**Conclusions** INPWT is an effective method for difficult wounds healing in patients with closed surgical wound and high risk of complications.

### Effect of low-power ultrasonic stimulation on the proliferation of human mesenchymal cells extracted from follicular fluid: possible applications in bone tissue engineering

L. Fassina*[1], C. Omes[2], F. Riva[1], D. Ogliari[1], C. Tinelli[2], G. Magenes[1], F. Benazzo[1]

[1]Università di Pavia (Pavia, IT);
[2]Policlinico San Matteo (Pavia, IT)

**Introduction** From the literature, it is known that low-power ultrasounds are able to remodel the cytoskeleton with consequent increased proliferation, enhanced expression of extracellular matrix proteins, and promotion of differentiation. In particular, ultrasounds stimulate the osteoblast proliferation, the production and the calcification of bone matrix and promote the osteoblastic differentiation of mesenchymal cells. Recently, a new source of human mesenchymal cells was discovered: the antral cells extracted from the follicular fluid.
**Materials and methods** Antral cells were obtained from 5 women with age between 30 and 37 years. These cells were seeded in number of 250,000, cultured in DMEM supplemented with 10 % FBS, 2 mM L-glutamine, 100 mg/ml penicillin, and then stimulated with low-power ultrasounds (frequency, 1.5 MHz; power, 149 mW; Igea, Carpi, Italy) for 2 or 5 min per day for a total of 4 days. At the end of the culture period, the proliferative capacity was evaluated by incubation with BrdU and its detection by immunofluorescence (the proliferative capacity is expressed as percentage of fluorescent cells).
**Results** The stimulation with ultrasounds for 2 min per day, compared to the unstimulated control, showed no significant changes in the proliferative capacity of mesenchymal cells (11 % in control *vs* 15 % in 2 min stimulus, $p > 0.05$). In contrast, the stimulation for 5 min per day significantly increased the proliferative capacity compared to the previous cases (46 %, $p < 0.05$).
**Discussion** In these preliminary experiments, we have shown that a low-power ultrasonic stimulation is able to increase the proliferative activity of mesenchymal stem cells extracted from the human follicular fluid.
**Conclusions** In conclusion, the use of ultrasounds on this source of pluripotent stem cells is useful in those applications of bone tissue engineering where it is necessary to greatly expand the mesenchymal cells before their differentiation in vitro.

### Electromagnetic fields counteract IL-1β activity during chondrogenesis of bovine mesenchymal stem cells

A. Ongaro*[1], A. Pellati[1], S. Setti[2], M. De Mattei[1]

[1]Dipartimento di Morfologia, Chirurgia e Medicina Sperimentale, Università di Ferrara (Ferrara, IT);
[2]Laboratorio di Biofisica Clinica, IGEA (Carpi, IT)

**Introduction** Osteoarthritis (OA) is a common joint disease associated with articular cartilage degeneration. Cartilage damage is an irreversible process, because of the limited capacity of the adult articular chondrocytes to repair and regenerate the normal cartilage matrix architecture. To improve the therapeutic options of OA, tissue engineering based on the use of mesenchymal stem cells (MSCs) has emerged. However, the presence of inflammatory cytokines, such as interleukin-1β (IL-1β) during chondrogenesis reduces the efficacy of cartilage engineering repair procedures, by preventing chondrogenic differentiation. Previous studies have shown that pulsed electromagnetic fields (PEMFs) stimulate anabolic processes in OA cartilage and limit IL-1β catabolic effects. We investigated the role of PEMFs during chondrogenic differentiation of MSCs, isolated from bovine synovial fluid, in the absence and in the presence of IL-1β.
**Materials and methods** Pellets of MSCs were differentiated for 3 and 5 weeks with transforming growth factor-β3 (TGF-β3), in the absence and in the presence of IL-1β and exposed or unexposed to PEMFs. Proteoglycans (PGs) synthesis and PG content were measured by radioactive $^{35}$S-sulphate incorporation and dimethylmethylene blue assay, respectively. Real-time RT-PCR was performed to analyze type II collagen and aggrecan expression. Histological analysis for haematoxylin and eosin and for alcian blue and immunohistochemistry for type II collagen and aggrecan were carried out on pellet sections. For statistical analysis, comparisons between groups were performed using Student's *t* test and $p < 0.05$ level was considered significant.
**Results** Biochemical, quantitative real-time RT-PCR and histological results showed that PEMFs alone or in the presence of TGF-β3, play a limited role in promoting chondrogenic differentiation. Notably, in the presence of IL-1β and TGF-β3 a recovery on PG synthesis, PG content, aggrecan and type II collagen mRNA expression in the PEMF-exposed compared to unexposed pellets was observed. Also, in the same experimental conditions, histological and immunoistochemical results showed an increase in staining for alcian blue, type II collagen and aggrecan in PEMF exposed pellets.
**Discussion** The presence of inflammatory cytokines, such as IL-1β in human joints, due to arthritis or trauma, may explain why existing cartilage engineering repair strategies that rely on the in situ differentiation of MSCs, fail to provide a reliably successful. Our results support the hypothesis that PEMF treatment may favour chondrogenic differentiation in inflammatory conditions.
**Conclusions** This study shows a significant role of PEMFs in counteracting the IL-1β induced inhibition on chondrogenesis, suggesting a possible therapy for improving the clinical outcome of cartilage repair procedures.

### How different PEMF treatments can change *in vitro* functional response of tendon cells?

M. Viganò*[1], L. De Girolamo[1], D. Stanco[1], S. Setti[2], E. Galliera[3], V. Sansone[4]

[1]IRCCS Istituto Ortopedico Galeazzi (Milan, IT);
[2]IGEA S.p.A (Carpi, IT);
[3]Dipartimento di Scienze Biomediche, Chirurgiche ed

Odontoiatriche, Università degli Studi di Milano (Milan, IT); [4]Clinica Ortopedica, Università degli Studi di Milano, IRCCS Istituto Ortopedico Galeazzi (Milan, IT)

**Introduction** Conflicting studies on the effect of pulsed electromagnetic field (PEMF) on tendons have been conducted. We already showed that a single 1.5 mT PEMF exposure (PEMF-1.5 mT) was able to positively modulate the in vitro behaviour of tendon cells (TCs). To investigate a possible relation between PEMF dosage and TCs' response, we evaluated the effects of higher intensity (PEMF-3 mT) or repeated treatments at 1.5 mT (R-PEMF-1.5 mT).
**Materials and methods** The experiments were performed on TC populations isolated from semitendinosus and gracilis tendons of 7 donors who underwent anterior cruciate ligament reconstruction. TCs were exposed to different PEMF treatments (intensity: 1.5 mT or 3 mT; duration: 8 or 12 h; periodicity: single or three treatments every two days). DNA content and SCX, COL1A1 and VEGF expression were assessed by CyQUANT assays and real time-PCR, respectively, at 0 and 2 days after treatment. Moreover, the release of TNFα and IL-10 was assessed by ELISA.
**Results** Eight-hours of PEMF-3 mT and R-PEMF-1.5 mT exposure increased the TCs DNA content, although not significantly (+14 % and +24 %, respectively). On the other hand, 12-h of both types of treatment provoked a decrease of DNA content in all treated cells, in particular for R-PEMF-1.5 mT group (−20 %, $p < 0.05$). Differently from what observed for the single 1.5 mT treatment, gene expression of SCX and COL1A1 were relevantly reduced by PEMF-3 mT one, whereas the exposure of TCs to R-PEMF-1.5 mT was able to up-regulate VEGF ($p < 0.05$) and COL1A1, although not significantly due to the high interdonor variability. TNFα release was not relevantly affected by any treatment, whereas all treatments induced a significant increase of IL-10 in the culture medium ($p < 0.001$).
**Discussion** Our findings show that all the tested PEMF treatments were able to enhance cell proliferation, with the exception of 12 h R-PEMF-1.5 mT, which is probably too invasive for this in vitro model. Moreover, all the treatments were able to increase the release of anti-inflammatory cytokines, without affecting pro-inflammatory ones, thus demonstrating the feasibility of this approach. However, 3 mT PEMF is not beneficial for in vitro TCs, whereas 1.5 mT PEMF, above all as a single exposure, induced an up-regulation of tendon specific markers, possibly involved in tendon healing.
**Conclusions** Our in vitro study suggest that a single 1.5 mT-PEMF treatment allows to obtain the better biological response on this in vitro TCs model. Indeed, PEMF-3 mT seems to be too high, while 8 h R-PEMF-1.5 mT treatment can extend PEMF effect. Further analyses on different models, like whole tendon tissue specimens, are needed to confirm these observations.

### *C25–PAEDIATRIC ORTHOPAEDICS 1*

### Surgical reconstruction of the hip joint: our experience

N.M.A. Portinaro*[1], A. Panou[2], A. Peccati[2], F. Ronchi[2], C. Cucca[2], V. Monzani[2]

[1]Clinica Ortopedica, Università degli Studi di Milano (Milan, IT); [2]Istituto Clinico Humanitas (Rozzano, IT)

**Introduction** Alterations in the hip anatomy (dysplasia or subluxation, the neck-shaft angle and femoral anteversion) can affect the articular biomechanics and consequently gait, especially in patients affected by cerebral palsy (CP). The aim of the surgical treatment is to early restore the physiological anatomy of the hip joint avoiding more complex deformities, that require palliative treatment.
**Materials and methods** Between 2006 and 2012 we treated 102 hips belonging to 75 patients (44 males and 31 females, meanly aged 10.32 ± 3.59 years; 56 patients younger than 12) affected by CP (65 cases: 36 tetraplegic and 29 diplegic) or other neurological conditions (10 cases). The acetabular index, the migration percentage and the neck-shaft angle have been used for pre-operative and post-operative assessment. Patients were treated with pelvic and/or proximal femur osteotomy. In 63 cases ancillary procedures, such as psoas, adductors or hamstring release, were required.
**Results** The mean follow-up time was 32.61 ± 22.17 months. All the radiological parameters, measured both immediately after surgery and 1 year after surgery, showed a greater improvement in patients younger than 12, with no significant differences among each other. No re-subluxation or AVN were observed; in 1 case a partial bone graft re-absorption was detected.
**Discussion** The restoration of the functional anatomy of the hip joint is mandatory for a physiological articular biomechanics (which is needed to walk and maintain the standing position), especially in children affected by neurological conditions. The earlier the surgical reconstruction of the hip joint, the lesser the rate of recurrences or worsening of the radiological parameters.
**Conclusions** The early reconstruction of the hip joint promotes its anatomical and functional restoration, and decreases the failure and re-currences rate.

### Surgical techniques for correction of pes planus in children affected by cerebral palsy: our experience

N.M.A. Portinaro*[1], A. Panou[2], F. Ronchi[2], A. Peccati[2], V. Monzani[2]

[1]Clinica Ortopedica, Università degli Studi di Milano (Milan, IT); [2]Istituto Clinico Humanitas (Rozzano, IT)

**Introduction** Pes planus valgus represent one of the most functional deformities of the flexible lever arm in children with cerebral palsy (CP); the deformity alters, especially in walking patients, the support, the push, the plantar-flexion/knee-extension moment. The correction of this deformity allows improvement in gait and to correct the alignment of the lower limb.
**Materials and methods** Between 2006 and 2012 we surgically treated 250 feet of 141 patients (81 males and 60 females, meanly aged 15.20 ± 5.52 years). 61 patients were diplegic and were treated with calcaneal osteotomy (C1; 12 cases), cuboid-calcaneal osteotomy (C2; 2 cases) first cuneiform-cuboid-calcaneal osteotomy (C3; 29 cases), talar-navicular resection/arthrodesis (medial pillar, MP; 18 cases); 14 patients were hemiplegic (5 C1 + 2 C2 + 4 C3 + 3 MP); 41 patients were tetraplegic (2 C1 + 2 C2 + 1 C3 + 36 MP); 25 patients were diagnosed foot deformities due to other causes.
**Results** The mean follow-up time was 32.27 ± 16.64 months. The anatomy was restored in all cases in the first post-operative follow-up, while 12 and 24 months after surgery 8 and 9 recurrences were respectively observed; in 1 case (treated with MP) a nonunion was observed, which required a further surgery, and 1 case (treated with C3) required a new osteotomy. Skin ulcers were observed in 6 patients 2 weeks after the plaster cast was applied and in 3 patients after the plaster cast removal 6 weeks after surgery.
**Discussion** Plantigrade support is mandatory for a correct gait pattern and the maintenance of the standing position. Pes planus, the most common foot deformity in children affected by CP, alters the distal lever arm moment decreasing the power during the toe-off phase of the gait, making walking and standing more mechanically inefficient.
**Conclusions** The complete restore of the local anatomy and the physiological alignment of the foot pylons are necessary in order to improve the lower limb cinematic and kinetic, especially in patients affected by cerebral palsy.

### Surgical techniques for pes planovalgus deformity in cerebral palsy

P.F. Costicio*, R. Russo, O. Palmacci

Ospedale Pediatrico Bambino Gesù (Rome, IT)

**Introduction** Pes planovalgus deformity in patients with cerebral palsy is very frequent and often due to muscle imbalance, resulting in a predominance of the pronatory versus the supinatory forces.
**Materials and methods** We report a total of 450 surgical procedures. Grice subtalar extra-articular arthrodesis was performed for pes planovalgus deformity on 250 feet of patients with cerebral palsy. Range of age : 8–23 years. Eighty percent of patients were quadriplegic. A fibular graft taken from the third of the fibula was used in all patients without any fixation material. Two hundred feet were treated with the double arthrodesis technique.
**Results** The double arthrodesis can yield a better correction in severe deformities, but cannot be performed under 12 years of age. On the other hand, Grice subtalar extra-articular arthrodesis is suitable for younger subjects, yet offering less long-term maintenance of surgical correction.
**Discussion** A severe foot deformity can reduce the plantigrade support and create secondary knee and hip deformities in patients with cerebral palsy. There is often uncertainty between an early surgical treatment and a later one, to be executed after the growing stop. The authors perform the Grice subtalar extra-articular arthrodesis from the age of 8 and the double arthrodesis technique from the age of 12.
**Conclusions** Pes planovalgus deformity in cerebral palsy is a deformity that produces foot medial overbearing with reduction of support base and balance difficulties. Both surgical techniques are useful to correct pes planovalgus deformity in cerebral palsy, recovering the balance of plantigrade support is important in patients with cerebral palsy. Grice technique is suitable for younger patients, while the double arthrodesis reduces relapse risk in severe structured deformities.

### *C26–PAEDIATRIC ORTHOPAEDICS 2*

### Congenital pseudarthrosis of the clavicle: report of 21 cases

L. Di Gennaro, M. Cravino*, A. Martinelli, C. Racano, O. Donzelli

Istituto Ortopedico Rizzoli (Bologna, IT)

**Introduction** Congenital pseudarthrosis of the clavicle is a rare disease. The lesion is usually unilateral (90 % of cases) and mainly localized in the middle third of the clavicle. The purpose of this paper is to describe our results on 21 consecutive cases.
**Materials and methods** We have re-evaluated the medical records and radiographs on 21 patients, 10 males and 11 females, affected by nonunion of the clavicle. No patient at diagnosis complained functional impairment of the upper limb, or neuro-vascular problems. X-rays were taken in all cases. Seventeen of the 21 patients were treated surgically, in 4 cases surgical treatment was rejected by the parents. In all cases the synthesis of fragments was performed using an intramedullary K wire, in one case a Sherman plate was used. In 4 cases autologous bone grafting from the iliac crest was used, in 1 case we used a fibular allograft from cadaver. All patients were treated postoperatively with immobilization in plaster.
**Results** The mean age of patients at diagnosis was 7 years (range 1–12 years). At follow-up (range 6 months to 19 years) no patient complained of pain, functional limitation or neuro-vascular impairment. The case treated with Sherman plate underwent second surgery due to infection, hardware removal was performed. All cases but one treated with homologous bone grafting, achieved bone union between 2 and 4 months after surgery. The aesthetic result was good in all cases.
**Discussion** The surgical treatment of congenital pseudarthrosis of the clavicle is still a controversial topic. Considering the possible complications (thoracic outlet syndrome, pain and functional impairment in adulthood) we think that surgical treatment should be considered as viable option only when the clinical examination shows an evident bulging.
**Conclusions** Our survey leads us to state that surgical treatment should include a complete excision of the nonunion of the clavicle, intramedullary K-wire fixation and the use of an autologous bone graft to promote healing. In our opinion postoperative immobilization in plaster is mandatory.

### Mesenchymal stromal cells in the treatment of bone nonunions in patients with achondroplasia

A. Memeo, E. Panuccio*, F. Verdoni

Azienda Ospedaliera Gaetano Pini (Milan, IT)

**Introduction** Achondroplasia is the most common genetic form of dwarfism with the appearance of disproportionately short stature. Lower limb lengthening, has become one of the standard modalities of treatment for this body disproportion (Paley 1988, Cai et al. 2004). In recent years, there have been a number of reports about the benefits and complications of this long and arduous process (Paley 1990, Cai et al. 2004, Shyam et al. 2009, Venkatesh et al. 2009). One of the complications which prolongs the time of treatment and determines the need for new surgical treatments is the non-union bone. We tested the hypothesis that achondroplasia may benefit from regenerative strategies based on mesenchymal stromal cells (MSC) as a source of growth factors when we have nonunion bone. The aim of the study was to verify the osteogenic properties of MSC correlated with the clinical and radiological outcome.
**Materials and methods** We performed a retrospective analysis of serial radiographs in fifteen patients in treatment for tibial lengthening (8 patient) and femur lengthening (7 patient) with external fixation. All patient were affected by refractory consolidation, and were been using MSC derived from the iliac crest (IC-MSC). We executed eight infiltration of tibia and seven of femur. MSC was been injected in solution into tibia or femur around the resection line or bone defect. MSC injections were repeated three times at a distance of one month. After the MSC transplantation, the patients were monitored during a 10-month follow-up period.
**Results** Bone consolidation was obtained in all patient without further surgery, after ten month by treatment. We could see no sign of aberrant bone formation or malignant transformation.
**Discussion** MSC promoted osteoinductive activity of bone and improved the overall structure of bone.
**Conclusions** Our data suggest that cell therapy may be a useful tool for the treatment of refractory consolidation because it increases the opportunity to achieve effective bone tissue regeneration, the presence of pro-osteogenic growth factors is an essential requirement for bone healing.

### *C27–PAEDIATRIC ORTHOPAEDICS 3*

### Paediatric radial neck fractures: one step percutaneus reduction and fixation

A.D. Cossio*, C. Cazzaniga, D.E. Gallone[1], G. Gridavilla[1], G. Zatti[1]

AO San Gerardo Monza (Monza, IT)

**Introduction** In paediatric traumatology radial neck fractures of third and fourth degree allowed to Judet classification are rare events, they account for 2–3 % of all paediatric fractures. Their relative low incidence is inversely proportional to the serious morpho-functional alterations which can follow to a wrong treatment. The purpose of this study is evaluate the results we achieved in our hospital, with a percutaneous reduction and fixation technique using 1 or 2 K-wires according to a modified Soo Mi Cha technique to obtain a better compliance and easier conduction.
**Materials and methods** Between 2010 and 2011, we treated 9 paediatric patients, aged from 6 to 11, with radial neck fractures of third and fourth degree allowed to Judet classification. All patients were operated by the same surgeon in a surgery time of about 15 min. The average follow-up was 12 months and periodical X-rays and clinical evaluations were carried out. The results were estimated radiologically (Metaizeau classification) and clinically (Mayo clinic elbow evaluation test).
**Results** X-ray follow-up (according to Metaizeau) were excellent in 8 cases and good in 1 case. Clinical results were excellent in each treated case. The only minor complication was a superficial skin infection cured by oral antibiotic and ended in few day treatment.
**Discussion** Even if recent articles have suggested some low invasive treatment, we think that a single surgery act avoiding further operations is the best option for our little patients.
**Conclusions** In spite of few patients, we are encouraged by the results to go on with this technique.

### School screening of scoliosis: 8837 children

E. Pratelli*, A. Petrocelli, V. Petrai, P. Pasquetti

AOU Careggi (Florence, IT)

**Introduction** Scoliosis is a common disease: almost 5–10 % of the population is affected by this condition, especially the female gender. The literature supports the idea that the screening is justified in order to identify the spine deformity, to set a correct treatment and to reduce the cases which need surgery.
**Materials and methods** From November 2010 to June 2012, was performed a spinal deformities screening in 98 public schools of the District of Florence. The screening was conducted once a week, consisted in two level. During the first level, were investigated: ligamentous laxity (Beighton score), thoracic kyphosys (with inclinometer) and identification of those which could not be corrected, the presence of dimorphisms of the spine in the frontal plane. The etherometry of the lower limbs were reported and corrected before the search for a possible gibbus during the Forward Bending Test tests with measurement of ATR (angle of rotation of the trunk) using the Bunnell scoliometer. Only selected patients had access to the second level of the screening to define the diagnostic-therapeutic course.
**Results** Eight thousand eight hundred and thirty-seven (8837) students were screened of an expected total of 11206. The mean age was 11.15 years (range 9–18). The mean value of Beighton scale for joint laxity was 2,092/9 points (range 0–9). The mean value for thoracic kyphosys was 34.38° (range 9°–60°). Two thousand 2 hundred and eight (2208) students had a thoracic hyperkyphosis (>40°), not reducible in 39 cases. On the total amount of 8837 students screened, 7553 (85.47 %) had no significant spinal deformities on the frontal plane. One thousand two hundred and fifty (1250) children (14.14 %) have been clinical diagnosed for scoliosis and were referred to a follow up evaluation after 6 months; in 200 of these we also required a radiographic exam. 999 subjects had a previous diagnosis of scoliosis, reconfirmed by our screening only in 258 cases (25.82 %). Among the 7838 children without previous scoliosis diagnosis, 992 (12.65 %) new diagnosis have been made.
**Discussion** Our results show a statistically significant correlation between the increasing severity of spinal deformities on the frontal plane and the increasing score of Beighton scale for joint laxity (Pearson's Chi squared $p < 0.0001$). During the screening we found new clinical diagnoses of scoliosis (false negatives) and false positives.
**Conclusions** Based on our experience using a trained staff to conduct the school screening we can significantly reduce the number of false positives and false negatives, thereby reducing healthcare costs.

### Forearm nonunions in children

G. Di Gennaro, A. Martinelli*, M. Magnani, C. Racano, O. Donzelli

Istituto Ortopedico Rizzoli (Bologna, IT)

**Introduction** We report our experience in the treatment of forearm nonunion in children.
**Materials and methods** Sixteen diaphyseal forearm nonunions (6 months after primary treatment) are reported in 16 children under 16 years of age. The average age was 10.8 years. Sites of nonunion were ulna (3), radius (8), and both (5). For each we assessed: type of fracture, form of initial treatment, type of nonunion, incidence of infection. All were post-injury lesions, most were closed fractures. Excluded from the study were congenital pseudarthrosis and nonunions after resections for bone tumours.
**Results** Open reduction and internal fixation with plate, intramedullary pin or a combination of these, contributed to nonunion. Inadequate casting time, premature removal of percutaneous pin fixation without splint protection, and wound infections also contributed to nonunion. Severe infection (osteomyelitis) was found in one 9.8 year old boy, a forearm fracture treated elsewhere with plates. Revision surgery was necessary to remove hard-wear and bone sequestration, and the nonunion healed with conservative methods. Others treatment of the forearm nonunion required excision of the nonunion fibrous tissue, bone grafting, and internal (intramedullary pin 8, plates 4) or external bone stabilization (3). Average follow-up 46 months; in all patients the nonunion healed in an average of 3 months (1.5–6).
**Discussion** In the literature there is little information dealing directly with nonunion in children. This study is an effort to gain some insight into infrequent problems of nonunion of the forearm in children.
**Conclusions** Paediatric nonunion is a rare condition, and it is often but not always due to an error in treatment. Forearm nonunion does occur in children, especially after the age of 7. Treatment must be individualized, but usually requires excision of the fibrous tissue, bone stabilization and grafting.
